# Decoding Synergistic Pathways in Bimetallic MOFs for Advanced Oxidation Processes

**DOI:** 10.1002/adma.74785

**Published:** 2026-08-25

**Authors:** Karim El‐Naggar, Huayang Zhang, Shaobin Wang

**Affiliations:** ^1^ School of Chemical Engineering Adelaide University Adelaide South Australia Australia; ^2^ Department of Chemistry Faculty of Science Ain Shams University Cairo Egypt

**Keywords:** advanced oxidation processes, bimetallic metal–organic frameworks, bimetallic synergy, Fenton, persulfate activation, photocatalysis, water treatment

## Abstract

Bimetallic metal–organic frameworks (BMOFs) offer a versatile platform for advanced oxidation processes (AOPs) because paired metal centers can regulate framework structure, charge distribution, redox cycling, oxidant activation, and reactive‐species generation. This review presents a mechanism‐oriented analysis of BMOF‐based AOPs for wastewater remediation, covering material design, synthesis, purification, activation, structural validation, and catalytic applications. Particular attention is given to validating genuine BMOF formation through phase, compositional, spatial, and local‐structural analyses, and to establishing bimetallic synergy through appropriate controls, leaching tests, spectroscopic evidence, and theoretical calculations. Cooperative pathways are compared across photocatalysis, H_2_O_2_ activation, peroxymonosulfate and peroxydisulfate activation, catalytic ozonation, and peracetic acid activation, with emphasis on how metal chemistry and local coordination regulate electronic structure, oxidant selectivity, reactive‐species generation, and catalytic performance. Cross‐study comparisons, metal leaching, aqueous stability, and performance in real‐water matrices are critically assessed, while catalyst recovery and long‐term operation are identified as key practical gaps. Finally, design principles and future priorities are proposed for developing stable, scalable, and mechanistically validated BMOFs for practical wastewater treatment. By integrating structural validation, mechanistic analysis, design principles, and engineering considerations, this review provides a basis for developing stable and scalable BMOF‐AOP systems for practical wastewater treatment.

## Graphical abstract

This review presents a unified framework linking metal pairing and framework design with electronic structure, redox cycling, oxidant activation, reactive‐species generation, and catalytic performance across photocatalytic and oxidant‐based BMOF‐AOPs. The resulting design principles guide the development of hydrolytically stable and efficient BMOFs for practical wastewater treatment.

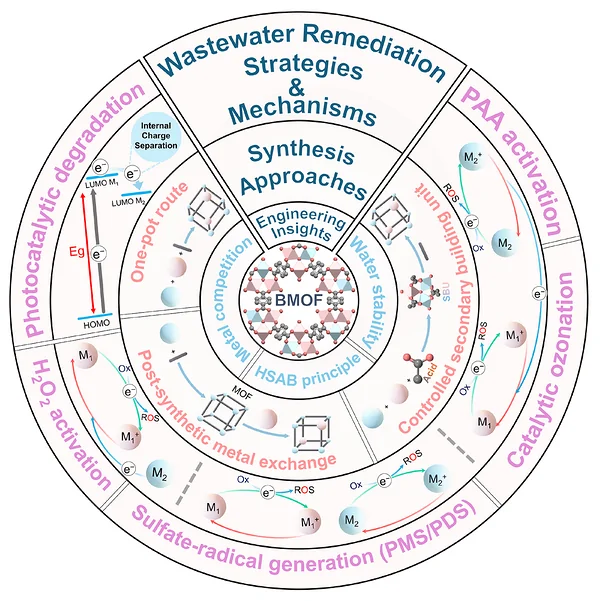

## 1 Introduction

Metal–organic frameworks (MOFs) are a class of porous hybrid materials formed by the self‐assembly of metal ions or clusters (referred to as metal nodes or secondary building units, SBUs) and multi‐chelating organic linkers [1]. This process yields crystalline networks with distinctive porous structures. The first rigid MOF, MOF‐2, was synthesized by reacting Zn(NO_3_)_2_ with 1,4‐benzene dicarboxylic acid (H_2_BDC) [2]. Since then, more than 65 metals and over 10 000 distinct organic linkers (Figure 1) have been employed in MOF synthesis, resulting in approximately 120 000 MOFs documented in the Cambridge Structural Database (CSD) with over 120 topologies, and more than 500 000 additional predicted structures [3, 4].

**FIGURE 1:**
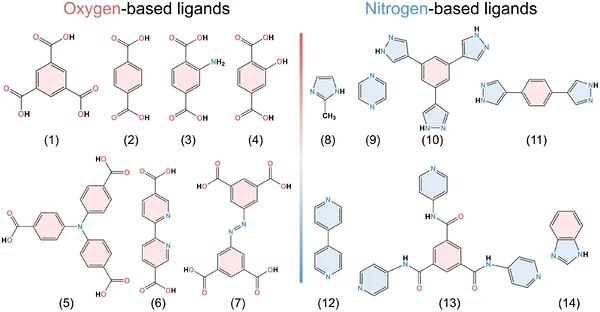
Structure of some oxygen‐ and nitrogen‐based ligands used in synthesizing MOFs. (1: 1,3,5‐benzenetricarboxylic acid (H_3_BTC; trimesic acid), 2: 1,4‐benzenedicarboxylic acid (H_2_BDC), 3: 2‐aminobenzene‐1,4‐dicarboxylic acid (2‐BDC‐NH_2_), 4: 2‐hydroxybenzene‐1,4‐dicarboxylic acid (2‐BDC‐OH), 5: 4,4′,4″‐nitrilotribenzoic acid (H_3_NTB), 6: 2,2′‐bipyridine‐5,5′‐dicarboxylic acid (5,5′‐Bpydc), 7: 5,5′‐(diazene‐1,2‐diyl)diisophthalic acid (H_4_ABTC), 8: 2‐methylimidazole (2‐MeIm), 9: pyrazine (Pyz), 10: 1,3,5‐Tri(4‐pyrazolyl)benzene (H_3_BTP), 11: 1,4‐benzenedipyrazolate (H_2_BDP), 12: 4,4′‐bipyridine (4,4′‐Bpy), 13: 1,3,5‐benzene tricarboxylic acid tris[*N*‐(4‐pyridyl)amide] (4‐Btapa), and 14: benzimidazole (BIm)).

Currently, various routes have been developed for MOF synthesis, including room‐temperature (RT) [5], solvothermal/hydrothermal [6], mechanochemical [7], ultrasonic (US)‐assisted [8], microwave (MW)‐assisted [9], and electrochemical synthesis [10], as well as other strategies [11].

MOFs are among the porous materials with the highest specific surface area (S.A.), reaching 10 000 m^2^ g^−1^ in select cases [1, 12]. Additionally, carefully designed MOFs can exhibit remarkable chemical, thermal, and water stability, along with permanent porosity and facile tunability of their pore size and shape [13]. These attributes have led to diverse applications in gas storage and separation [14], biomedicine [15], hydrogen production [16], water treatment [17], and analytical sensing [18, 19].

Surface modification strategies—including ligand functionalization, metal nanoparticle (MNP) incorporation, and composite formation (Figure 2)—can further enhance MOF performance across various applications [20].

**FIGURE 2:**
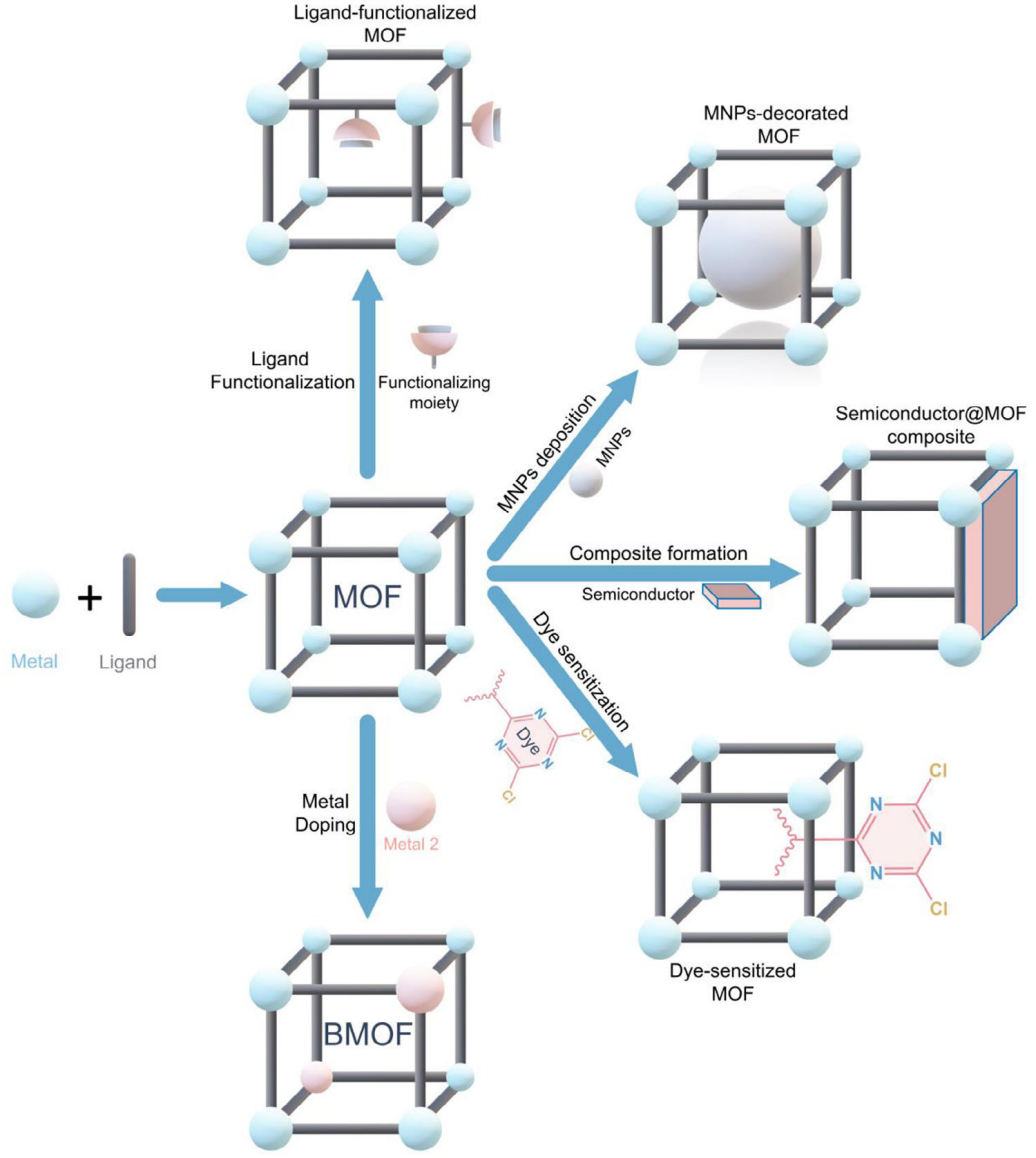
Schematic illustration of the various modification strategies for MOFs, including ligand functionalization, MNP deposition, composite formation, dye sensitization, and metal doping.

Ligand functionalization of MOFs can enhance stability, adsorption capacity, and optical response [21, 22]. For instance, replacing terephthalic acid (H_2_BDC) with 2‐aminoterephthalic acid (2‐NH_2_‐BDC) reduced the bandgap energy (*E*_g_) of Ti‐MOF from 3.67 to 2.65 eV, boosting the photocatalytic reduction of Cr(VI) from 30% to 75% [23].

Weak charge separation is a serious drawback that can greatly hinder the photocatalytic activity of MOFs. This limitation can be minimized by incorporating MNPs onto the MOF surface, a process termed metal nanoparticle deposition [24, 25], or through composite formation with other semiconductors (e.g., ZnO, CdS, Ag_2_O, TiO_2_, and graphene) [26, 27, 28, 29]. Both MNPs and semiconductors can capture electrons from the conduction band (CB) of the MOFs, thereby enhancing charge separation and photocatalytic activity. Additionally, dye‐sensitization can improve photocatalytic activity by enhancing light absorption [30].

Another promising modification involves doping monometallic MOFs with additional metals to create mixed‐metal MOFs (MM‐MOFs). MOFs incorporating two distinct metals within the SBUs are referred to as bimetallic MOFs (BMOFs). Early efforts to synthesize BMOFs, initially termed mixed metal coordination polymers before the widespread use of the term “MOFs”, began in the early 2000s [31, 32, 33]. Metal doping has been employed to enhance adsorption capacity [34, 35, 36], stability [34, 37], photocatalytic activity [38, 39, 40], and electron transfer in catalytic redox cycles [41].

The incorporation of two distinct metal centers into a MOF scaffold can generate bimetallic synergy, defined here as a cooperative effect that produces a catalytic, structural, or electronic response greater than the additive contribution expected from the corresponding monometallic components under matched conditions [5]. Therefore, higher activity than either monometallic counterpart alone is not sufficient to establish synergy and should be supported by appropriate control experiments and mechanistic evidence. In BMOFs, such synergy can be broadly classified into four categories (Figure 3): (i) structural synergy, resulting from changes in pore architecture [42], or the density and accessibility of coordinatively unsaturated metal sites (CUMSs); [43] (ii) electronic synergy, arising from intermetallic charge transfer [44], and electron density redistribution; [45] (iii) redox synergy involving accelerated metal redox cycling [46], or cooperative oxidant activation; [47] and (iv) sorptive (adsorptive–catalytic) synergy, in which pollutant enrichment near the active sites facilitates subsequent oxidative degradation [35, 48]. Combined with the intrinsic structural advantages of MOFs (high S.A., permanent porosity, and tunable pore geometry), these synergistic interactions have enabled BMOF applications across diverse fields, including cancer immunotherapy [49], photochemical reduction [50], hydrogen generation [51, 52], gas adsorption [53], glucose sensing [54]. MW absorption [55], oxygen evolution/reduction [56], supercapacitors [57], water oxidation [58], and wastewater treatment [5, 38, 40].

**FIGURE 3:**
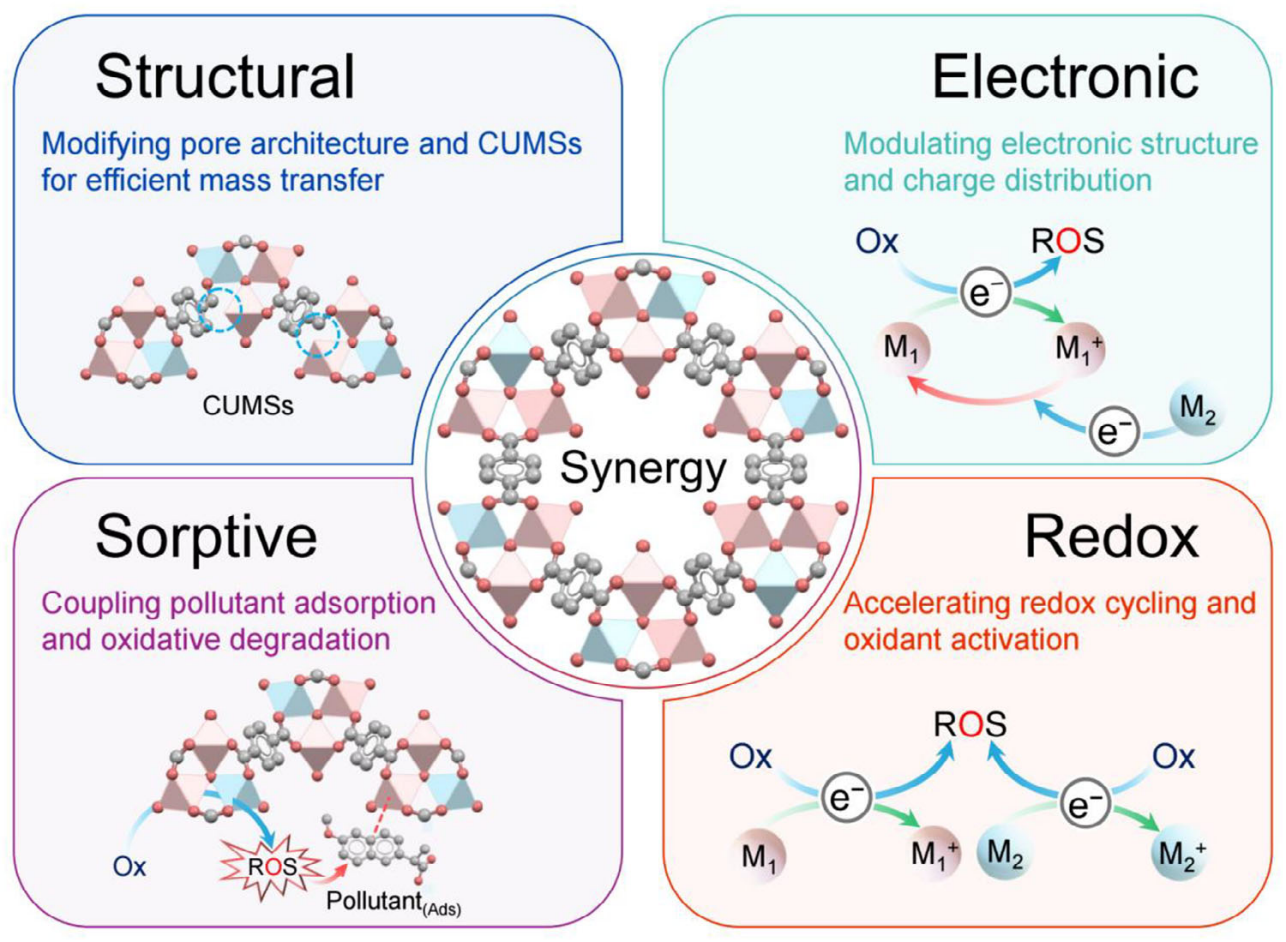
Main forms of bimetallic synergy in BMOFs, including electronic, structural, redox, and adsorptive–catalytic synergy.

Among the various water depollution strategies, such as adsorption and filtration, advanced oxidation processes (AOPs) are widely recognized as among the most effective [59]. AOPs, which encompass photocatalytic and oxidant‐based systems [60], rely on generating highly reactive species, including hydroxyl radicals (^•^OH), superoxide radicals (O_2_^•−^), singlet oxygen (^1^O_2_), and sulfate radicals (SO_4_^•−^), to oxidatively degrade complex organic contaminants [61]. Compared to traditional physical and biological methods, AOPs offer significant advantages in wastewater treatment: they are eco‐friendly, do not yield hazardous sludge, and can completely mineralize organic pollutants into harmless end products [62, 63].

Despite remarkable progress in the synthesis and applications of BMOFs, a dedicated review of their roles in AOP‐driven wastewater treatment remains lacking. Abednatanzi et al. summarized characterization and computational approaches for elucidating the structures and properties of MM‐MOFs [64], whereas Chen et al. reviewed BMOF synthesis and derived materials for energy and sensing applications [65]. Fan et al. discussed BMOFs for H_2_O_2_ and persulfate activation [66]. Kayani et al. reviewed BMOF‐based heavy‐metal adsorption [67]. Zhou et al. discussed BMOFs and MOF‐derived composites for electro‐ and photoelectrocatalysis [68], and Saini et al. summarized their applications in CO_2_ conversion, hydrogen evolution, and photocatalytic pollutant degradation [69]. Although these reviews provide valuable insights into specific aspects of BMOF chemistry and applications, they do not establish a unified mechanistic framework explaining how paired metal centers cooperatively regulate electron transfer, redox cycling, oxidant activation, and reactive species generation across diverse BMOF‐based AOPs (BMOF‐AOPs). Importantly, emerging oxidation systems, including catalytic ozonation (CA‐O_3_) and peracetic acid (PAA) activation, have not yet been comprehensively reviewed in the context of bimetallic cooperation in BMOF‐AOPs. Consequently, a comprehensive, mechanism‐oriented review encompassing photocatalysis and oxidant‐driven systems remains urgently needed.

To address this gap, this review provides a comprehensive and mechanism‐oriented perspective on BMOF‐AOPs for wastewater treatment, highlighting recent developments and the unique advantages of BMOFs for sustainable water purification (Figure 4). It first examines the fundamental aspects of MOFs and their modification techniques, including ligand functionalization, MNP incorporation, composite formation, and metal doping for BMOF construction. The review then addresses the engineering factors governing successful BMOF synthesis, covering fabrication methods such as the one‐pot route (OPR), post‐synthetic metal exchange (PSME), and controlled secondary building unit (CSBU) assembly, along with purification and activation techniques. Emphasis is placed on sustainable and scalable BMOF synthesis, together with a structural, compositional, and electronic validation roadmap for confirming bimetallic incorporation. It subsequently examines BMOF applications in wastewater treatment across major AOPs, including photocatalysis, H_2_O_2_ activation, sulfate‐radical‐based oxidation (via PMS and PDS), and the emerging CA‐O_3_ and PAA activation systems. The discussion provides a systematic comparison of degradation mechanisms, reactive species generation, and the catalytic roles of paired metal centers. Finally, the review explores the existing limitations of BMOF‐AOPs and offers perspectives on future research toward developing efficient, scalable BMOFs for real‐world wastewater treatment.

**FIGURE 4:**
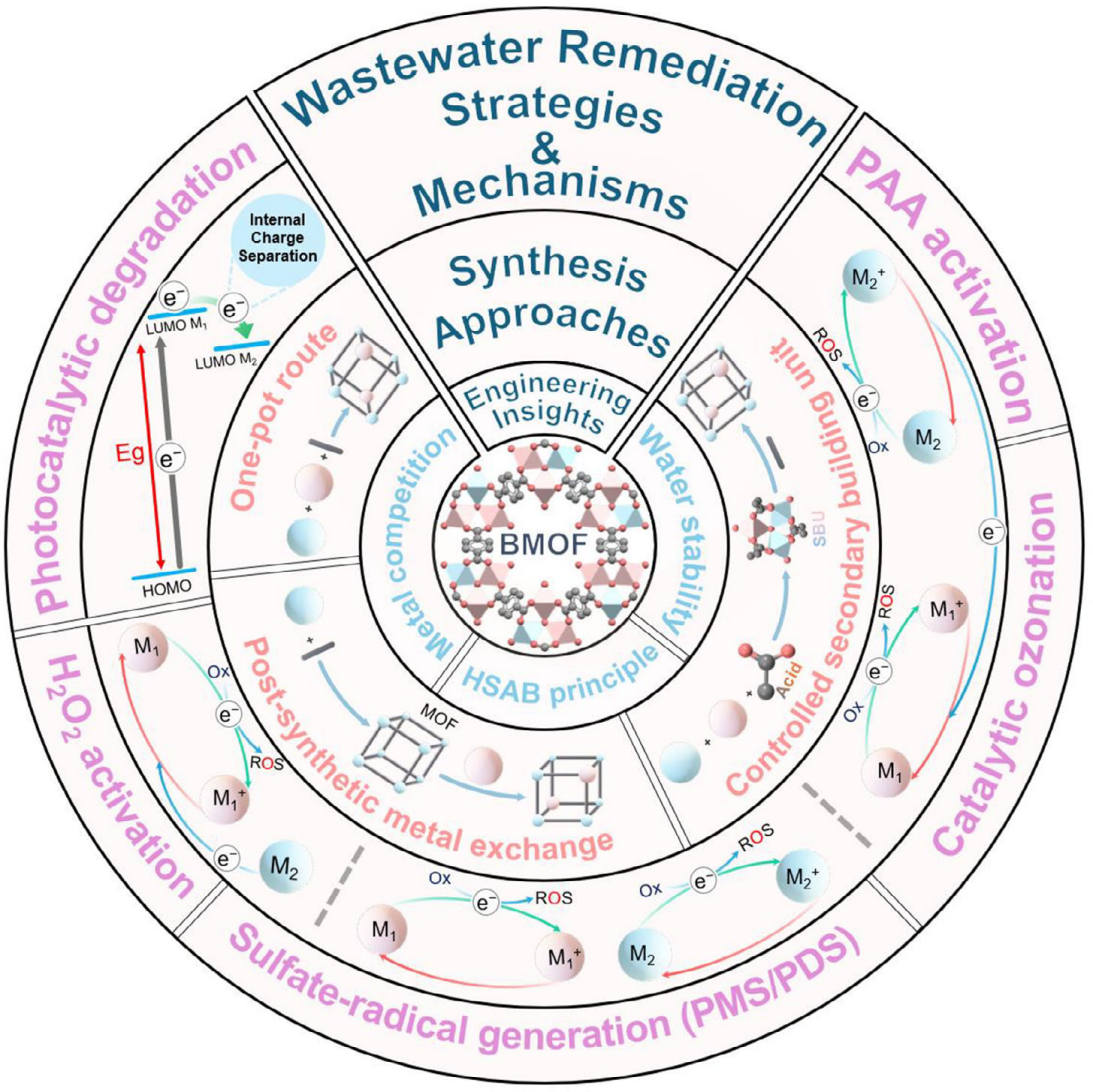
A conceptual framework outlining the key factors involved in the development and application of BMOFs for wastewater remediation. The diagram highlights engineering insights, synthesis strategies, various advanced oxidation processes, and their corresponding mechanisms.

## 2 Synthesis and Activation of BMOFs

As previously established, BMOFs comprise two distinct metal ions distributed throughout the framework. Ensuring a strong affinity of both metals for the organic linker is essential for achieving a stable structure. Furthermore, judicious selection of metals with compatible properties can synergistically enhance catalytic activity, adsorption capacity, and/or electrical properties [70]. This section briefly discusses the engineering factors that impact successful BMOF synthesis, different synthetic approaches, and purification and activation strategies.

### 2.1 Factors Affecting BMOF Fabrication

Metal competition and structural stability are crucial factors that must be evaluated prior to BMOF preparation.

#### 2.1.1 Mutual Competition of Metals

Each metal exhibits a distinct affinity for the organic linker, making it crucial to select metals with comparable binding affinities to ensure successful BMOF formation. In other words, combining incompatible metals may result in the formation of either single or mixed monometallic MOFs, rather than the desired BMOF. For example, Serre et al. reported the synthesis of MIL‐53(Cr/Fe) under hydrothermal conditions [71]. It is noteworthy that Fe(III) exhibits significantly higher reactivity toward terephthalic acid than Cr(III), likely due to its greater effective nuclear charge and crystal field stability [72]; as a result, it reacts with H_2_BDC more rapidly than Cr(III). Thus, the reaction between Fe(III) and Cr(III) with terephthalic acid yielded MIL‐53(Fe) instead of MIL‐53(Fe/Cr). However, using the less reactive metallic iron source (Fe(0)) enabled concomitant reaction with Cr(III) and H_2_BDC, resulting in the successful formation of an Fe/Cr BMOF.

#### 2.1.2 Water Stability of BMOFs

The water stability (or hydrostability) of MOFs refers to their resistance to hydrolysis in aqueous environments [64]. Among the various factors influencing MOF stability—including operating conditions, pore hydrophobicity, and metal–ligand coordination geometry—metal–ligand bond strength and ligand basicity are the most critical [13, 73, 74, 75, 76].

##### 2.1.2.1 Hard–Soft Acid–Base (HSAB) Principle

Bond strength can be described and governed by the HSAB principle, which states that hard acids preferentially bind hard bases, and soft acids bind soft bases [77, 78, 79, 80]. In MOF chemistry, carboxylate‐binding ligands (hard bases) form strong bonds with high‐valent metal ions (hard acids), such as Cr(III), Al(III), Fe(III), Ti(IV), Zr(IV), and Mn(IV). In contrast, nitrogen‐binding ligands (soft bases, e.g., imidazole and pyrazole) form strong bonds with low‐valent metals (e.g., Co(II), Zn(II), Cu(II), Mn(II), and Ni(II)).

##### 2.1.2.2 Metal–Ligand Bond Covalency

Bond strength can also be influenced by the degree of covalency of the metal–ligand bond. As the oxidation state of the metal increases, the degree of covalency with carboxylate‐based ligands also increases, leading to enhanced MOF stability [64]. For instance, Mn(IV) ions form strong bonds with carboxylates, exhibiting 50% covalency, whereas Mn(II) ions exhibit only 10% covalency with the same ligands [64, 73]. Therefore, doping a hard‐acid–hard‐base MOF with a softer acid can reduce its stability.

##### 2.1.2.3 Ligand Basicity

Additionally, the basicity of organic linkers can influence framework stability [74]. As the p*K*_a_ of the ligand increases, its basicity increases, and MOF stability improves [64]. For example, pyrazolate‐based MOFs (p*K*_a_ of pyrazole ≈ 19.8) and imidazolate‐based MOFs (p*K*_a_ of imidazole ≈ 18.6) demonstrated high stability under humid conditions [81, 82, 83, 84].

##### 2.1.2.4 SBU Stability

BMOF stability is also influenced by the stability of their SBUs. For instance, MOF‐5 is highly water‐sensitive, undergoing violent attack by water molecules in aqueous environments or even under atmospheric conditions, due to weak bonds between Zn and the oxygen of the terephthalate ligand [85]. This attack results in phase transformation and framework collapse [86]. Stability has been enhanced through various approaches, such as ligand functionalization with hydrophobic moieties [87], composite formation with carbon nanotubes (CNTs) [88, 89], and post‐synthetic annealing [90]. Ni(II) doping has been shown to significantly improve MOF‐5 stability [91]. Results indicated that pristine MOF‐5 underwent destruction after two days of exposure to 30%–37% humidity, while Ni‐doped MOF‐5 retained its structure even after one week of exposure. This improvement was attributed to the enhanced hydrostability of the Ni_x_Zn_4‐x_O SBU relative to Zn_4_O. Additionally, Ni doping in MIL‐101(Cr) significantly reduced chromium leaching and improved hydrostability across various pH values (2, 7, and 11), owing to the formation of SBUs involving Ni─O bonds, which protect the Cr SBU [37].

### 2.2 Synthesis of BMOFs

BMOFs can be synthesized using three different strategies (Figure 5): one‐pot route, post‐synthetic metal exchange, and controlled secondary building unit assembly.

**FIGURE 5:**
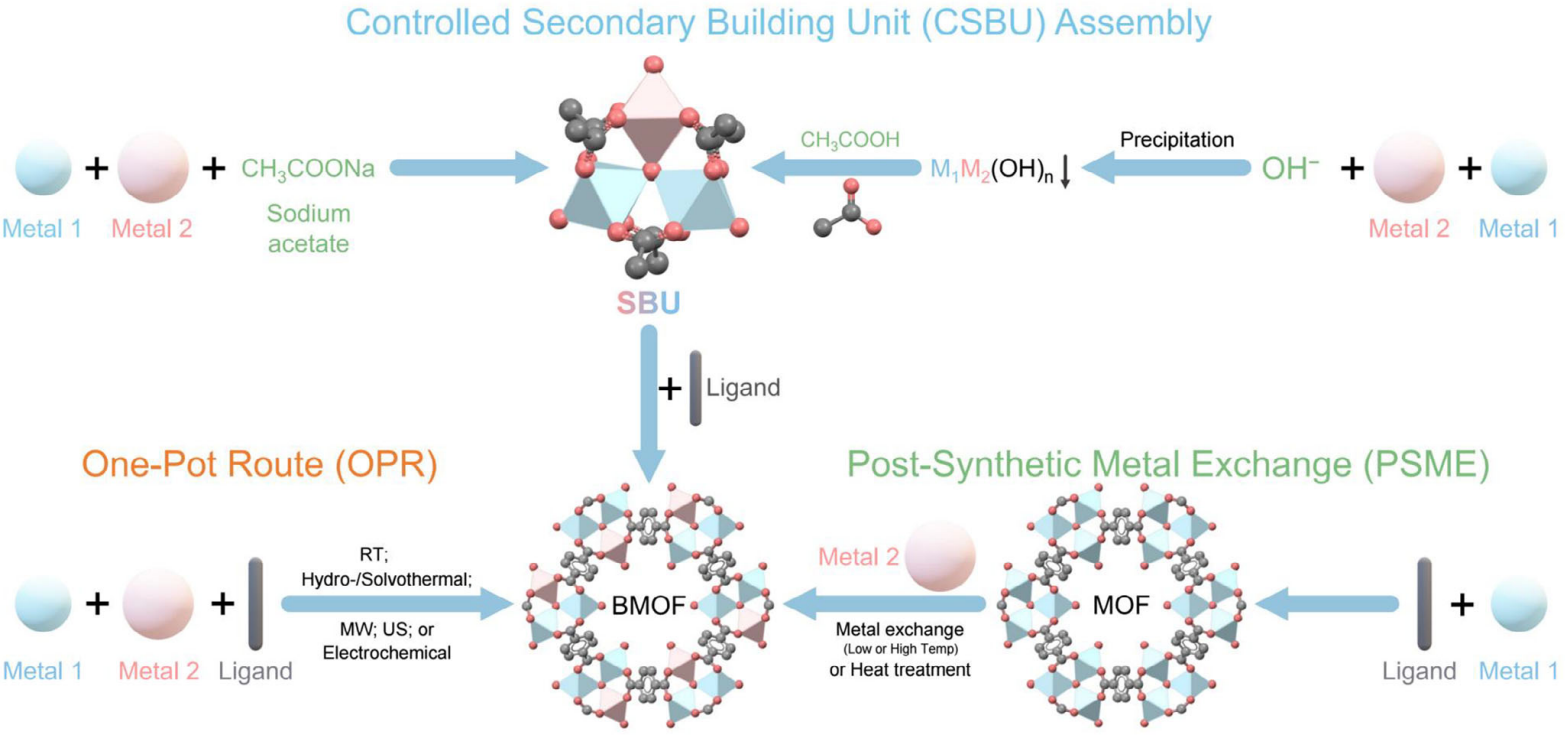
Diverse synthetic approaches of BMOFs, including the one‐pot route, post‐synthetic metal exchange, and controlled secondary building unit approaches.

#### 2.2.1 One‐Pot Route (OPR)

In this process, two metals react simultaneously with an organic linker [64], and the extent of this reaction depends heavily on the activation energy (*E*a) of the metal–linker coordination process [92]. For BMOFs with low *E*a, stirring a mixture of metal salts and ligands at ambient temperature is sufficient for BMOF formation, as demonstrated by Siew et al. in the synthesis of CuFe‐BTC BMOF at room temperature [93].

For BMOFs with moderate *E*a, mild heating may be sufficient. For instance, Yuan et al. synthesized CuCo‐ZIF by heating an ethanolic solution of Co(II), Cu(II), and 2‐methylimidazole (2‐MeIm) at 80°C using a water bath [94]. Additionally, refluxing a mixture of NH_2_‐BDC, Al(III), and Fe(III) at 150°C enabled the formation of NH_2_‐MIL‐101(Fe/Al) [95]. In some cases, extreme conditions of pressure and temperature are imperative for the synthesis of BMOFs with high *E*a. For example, BMOFs of M (Co or Ni) and Zr, referred to as M‐MOF‐801, were synthesized using a one‐step solvothermal treatment [96].

A variety of unconventional methods have been employed for the synthesis of BMOFs, showcasing their versatility. For instance, ultrasound‐assisted synthesis has been utilized to prepare Co/Zn‐imidazolate‐based BMOFs [97], while electrochemical cells have been successfully applied to produce flower‐like Ni/Co‐BTC [98]. Combining two strategies in the synthesis of BMOFs has also been reported. In this context, integrating MW radiation with the solvothermal technique—resulting in MW‐assisted hydro/solvothermal synthesis—can significantly reduce reaction time, enhance morphology, increase reaction yield, and improve catalytic activity [99, 100, 101]. For example, Xiong and coworkers reported the synthesis of MIL‐53(Fe, Cu) using a MW‐assisted solvothermal strategy [99]. PDS catalytic activation was higher than that of the analogous BMOF synthesized via the traditional solvothermal route. Systematic investigation of the activation energies of BMOF crystallization processes—and their dependence on metal combinations, linker chemistry, and solvent systems—represents an important yet largely unexplored research direction that could provide rational guidelines for energy‐efficient and scalable synthesis optimization.

#### 2.2.2 Post‐Synthetic Metal Exchange (PSME)

Numerous attempts to synthesize BMOFs using the direct one‐pot synthetic strategy have been unsuccessful [102, 103], likely due to incompatible metal affinities for the linker [71]. As an alternative, PSME can serve as a viable pathway [104]. PSME, achieved by soaking the MOF in a solution of a new metal, involves bond breakage between the organic linker and the original metal, followed by the formation of new bonds with the foreign metal ion [105]. However, the ease and extent of metal exchange in PSME are influenced by multiple factors, with the stability of the original and doped MOFs being paramount [105, 106].

In some cases, metal exchange can be completed within just a few hours at room temperature. For example, immersing Cd‐MOF in a Cu(II) solution at room temperature yielded CdCu‐BMOF within 4 h [107]. In contrast, substituting Co(II) in the cobalt‐based MOF DUT‐49(Co) with Cd(II) required 72 h, likely due to the bulkier ionic size of cadmium [103]. However, replacing half of the Zr content in NH_2_‐UiO‐66 with Ti required heating at 120°C for four days, owing to the robust Zr─O coordination bonds [108].

A special case of PSME entails redox‐induced metal transformations within frameworks to generate a bimetallic system in situ. For instance, vacuum activation of MIL‐100(Fe) partially reduced Fe(III) to Fe(II), resulting in a mixed‐valence MOF that qualifies as a special case of BMOFs [109].

#### 2.2.3 Controlled Secondary Building Unit (CSBU) Assembly

Although the OPR and PSME are straightforward, achieving precise control over the extent and distribution of each metal within BMOF structures remains challenging. Serre et al. proposed a controlled secondary building unit (CSBU) approach to precisely design hybrid porous Fe‐MOFs [110]. This approach involves preparing trimeric SBUs, also known as trimeric oxo‐clusters, consisting of three metallic octahedra linked by µ_3_‐O (Figure 6a, left). Upon their interaction with the organic linker, a direct exchange occurs between the labile acetates and the linker, resulting in the formation of a 3D MOF (Figure 6a, right). Therefore, these clusters serve as framework‐building units. The use of SBUs preserves the integrity of the metal precursors during the reaction with the linker, ensuring precise structural control.

**FIGURE 6:**
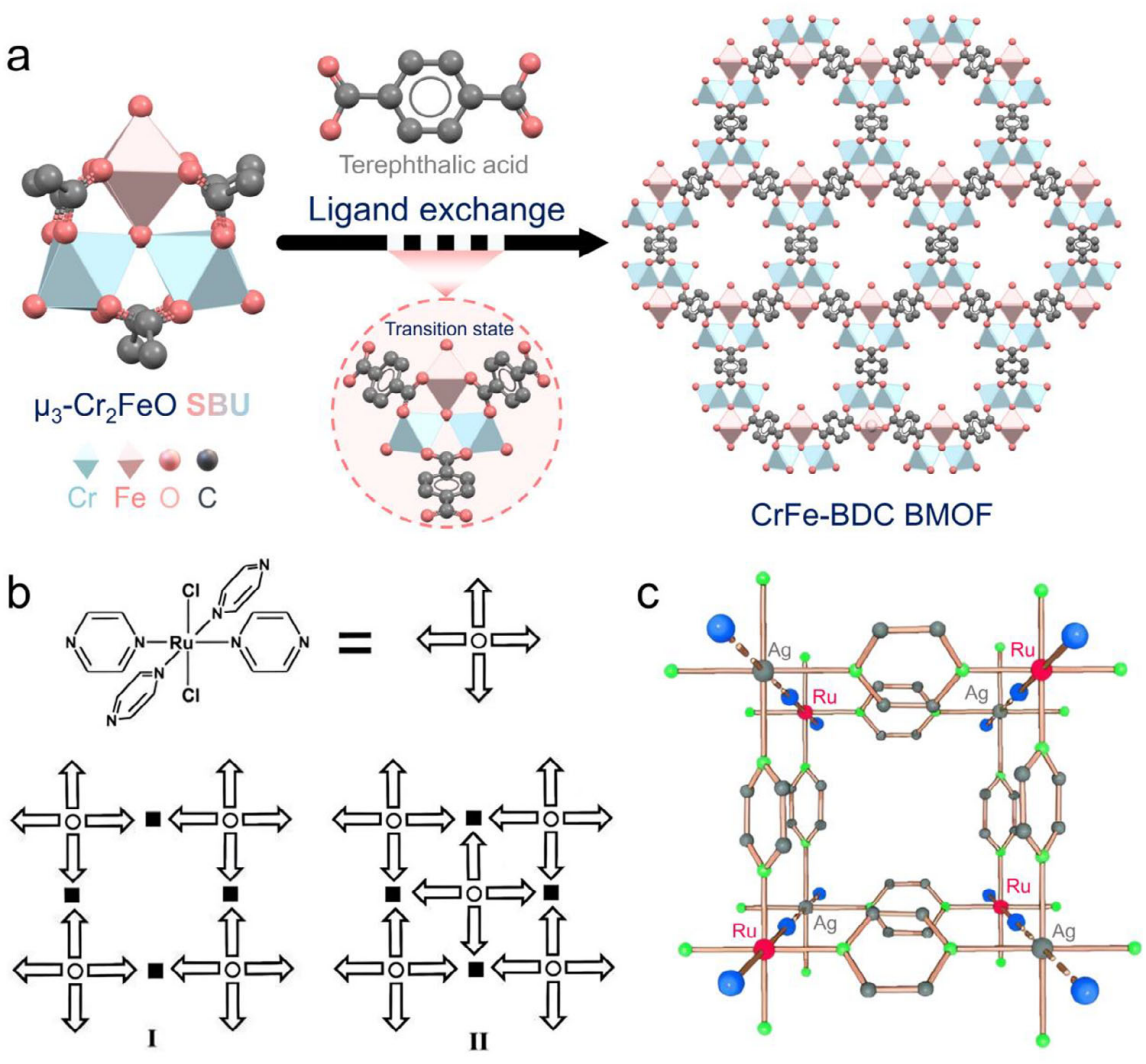
(a) Schematic illustration of CrFe‐BDC BMOF formation via ligand exchange between the (µ_3_‐Oxo)‐hexakis(µ_2_‐acetato)‐triaqua‐di‐chromium(III)‐iron(III) cluster (µ_3_‐Cr_2_FeO SBU) and H_2_BDC; hydrogen atoms and backward‐directed terephthalate ligands are omitted for clarity. (b) Structure of trans‐[RuCl_2_(pyz)_4_] and its possible coordination modes with another metal (o = Ru and ■ = Ag), and (c) building block of the resulting 3D Ru–Ag network; Reproduced with permission [111]. Copyright 2002, John Wiley and Sons.

Various methods have been developed for synthesizing oxo‐clusters. One approach involves precipitating transition metals as metal hydroxides, followed by subsequent reaction with acetic or formic acid to yield trinuclear acetate or formate clusters [112]; this strategy was later refined to achieve high‐yield homometallic and heterometallic clusters [113]. Alternatively, Knowles et al. prepared clusters of [MFe_2_(III)(µ_3_‐O)(µ_2_‐O_2_CCF_3_)_6_(H_2_O)_3_] through a one‐step reaction of metal salts with an acetate salt, where M represents Co(II), Ni(II), Cu(II), and Zn(II) [114].

Heterometallic SBUs are excellent building units for BMOFs. For example, a series of isostructural monometallic and bimetallic MOFs was synthesized through the interaction of SBUs with the corresponding ligands [115]. Similarly, Lionet et al. fabricated MIL‐126(FeM) by reacting µ_3_‐Fe_2_MO clusters (where M = Ni(II), Co(II), Mn(II), and Cr(III)) with 4,4′‐biphenyldicarboxylic acid (bpdc) [116].

A special case of CSBU, termed complex ligand assembly, involves complex formation between a first metal (M_1_) and a bifunctional ligand. The resulting complex features terminal functional groups capable of binding to another metal (M_2_). Thus, the pre‐synthesized complex serves as a ligand for M_2_. This strategy not only enables the design of BMOFs with a defined spatial arrangement of metal ions within the framework but also provides precise control over the metal–metal interactions [32]. For example, reaction of the as‐prepared *trans*‐[RuCl_2_(pyz)_4_] complex (Figure 6b) with Ag ions yielded a 3D Ru–Ag network (Figure 6c) [111]. Similarly, a β‐diketonate beryllium complex was employed to prepare Co/Be and Cu/Be networks upon reaction with Co and Cu salts, respectively [117]. Other BMOFs have been synthesized using a similar approach [31, 32, 33].

The advantages and disadvantages of the various synthetic strategies are summarized in Figure 7. OPR is a simple and scalable method but may result in mixed phases and limited control over metal distribution. PSME, however, allows precise tuning of metal composition but can be time‐consuming and unsuitable for all MOFs, particularly those with low stability. In addition, it requires highly crystalline, pure monometallic MOFs. CSBU also offers high control over metal distribution and enables the synthesis of highly ordered BMOFs; however, it is relatively complex, requiring precise conditions, multiple steps, and highly pure oxo‐clusters. Each method has distinct advantages and limitations, making the choice dependent on the desired application and framework complexity.

**FIGURE 7:**
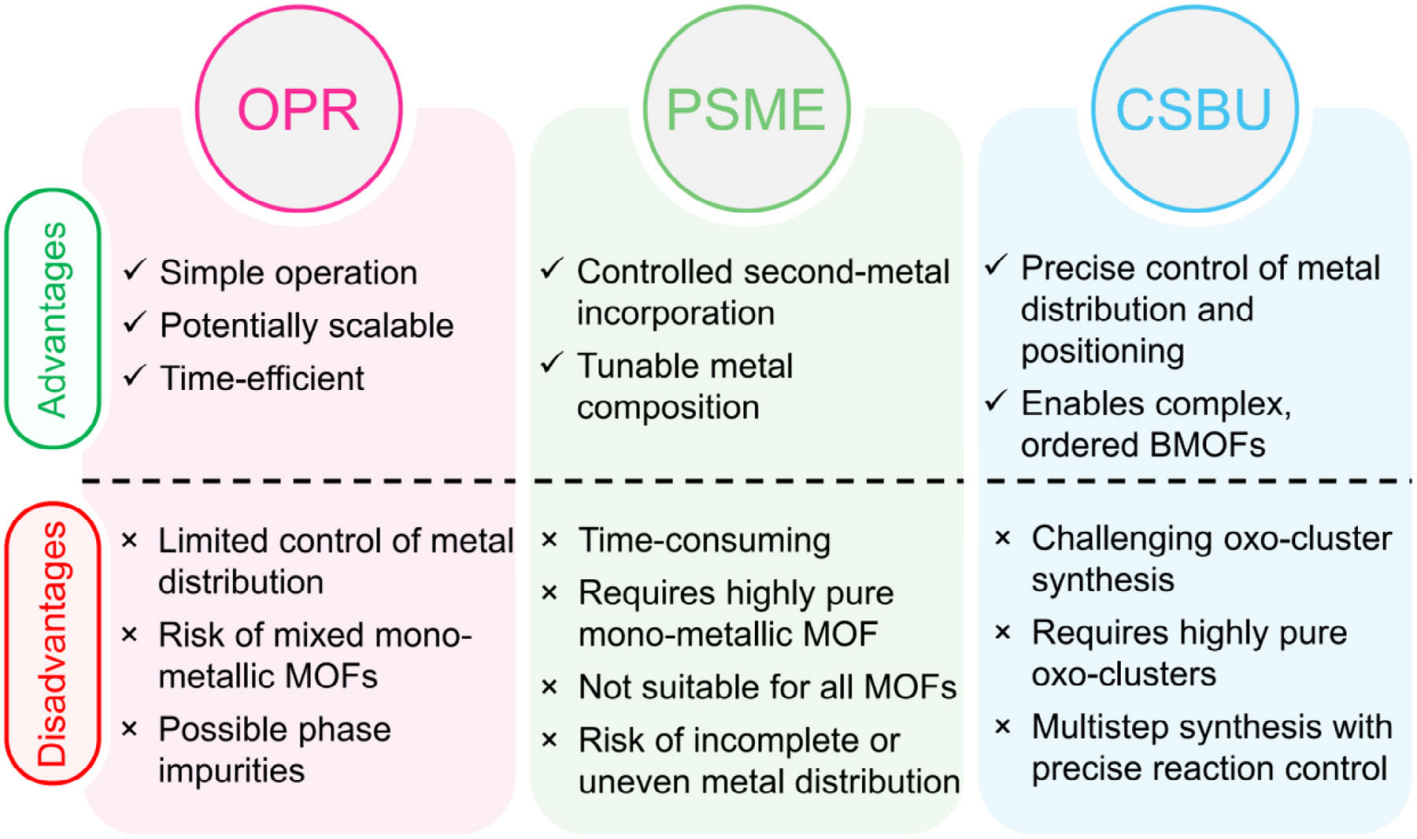
Advantages and disadvantages of OPR, PSME, and CSBU strategies for the preparation of BMOFs.

### 2.3 Sustainability and Scalability of BMOF Synthesis

While the synthetic strategies and purification protocols discussed above enable structurally diverse, compositionally precise BMOFs at the laboratory scale, their translation to real‐world applications demands a critical reassessment of the reagents, solvents, and conditions employed. Bridging this gap requires replacing conventional petrochemical feedstocks with sustainable precursors, and eliminating hazardous solvents, elevated temperatures, prolonged reaction times, and modulators in favor of greener, ambient synthesis conditions that preserve the structural and functional integrity of BMOFs. Three emerging strategies have shown particular promise (Figure 8): (i) linker salts to eliminate organic solvents, (ii) valorization of waste metal streams as sustainable metal sources, and (iii) upcycling of plastic waste as a renewable linker feedstock.

**FIGURE 8:**
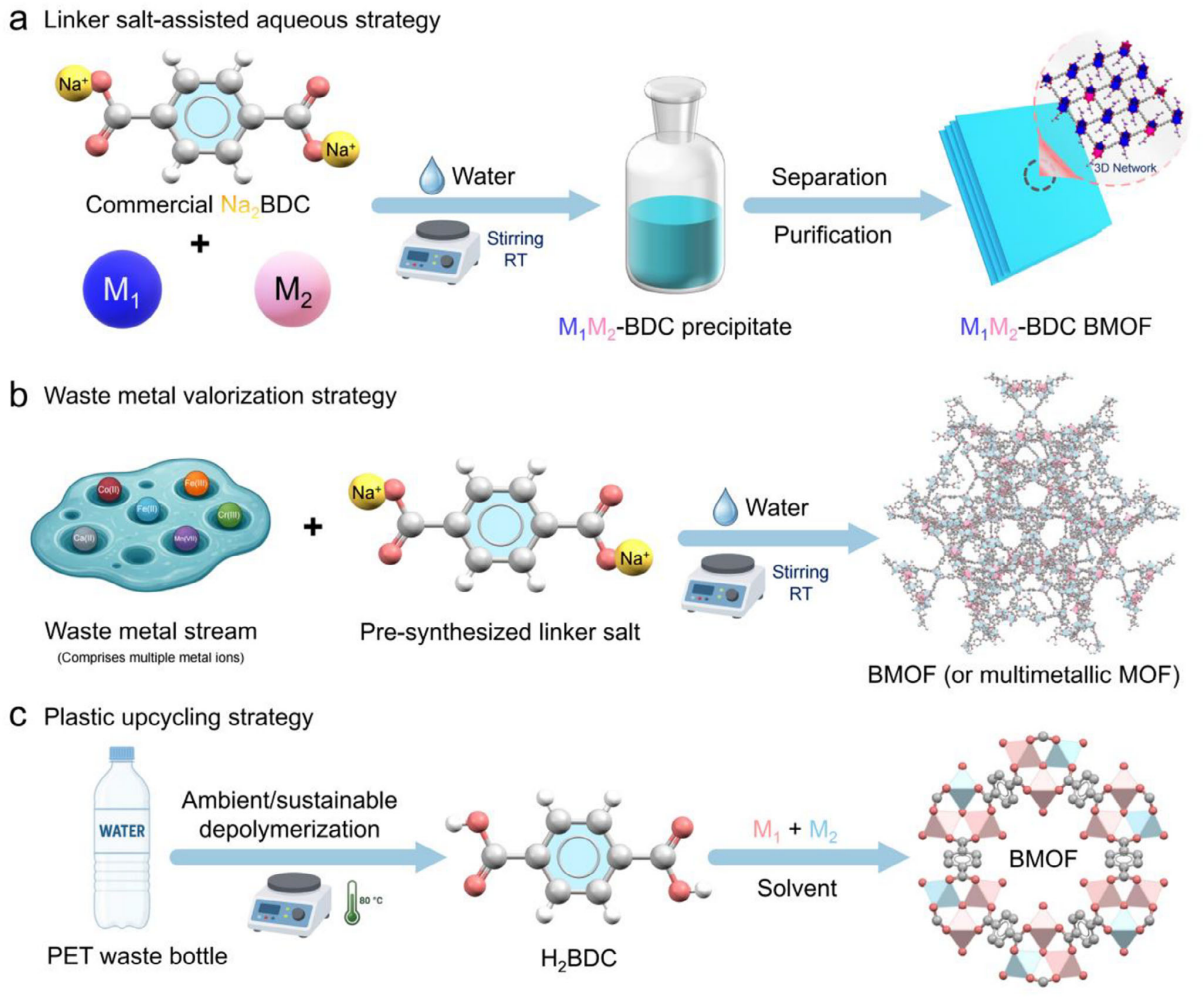
Sustainable strategies for BMOF synthesis: (a) linker salt‐assisted synthesis, (b) waste metal valorization, and (c) plastic upcycling.

Among the growing strategies to eliminate organic solvents is the use of linker salts. Recently, we developed a room‐temperature, aqueous synthesis for a two‐dimensional (2D) CuCo‐BDC BMOF using commercially available disodium terephthalate (Na_2_BDC) as the linker source (Figure 8a) [5]. The higher reactivity of Na_2_BDC relative to H_2_BDC enabled the use of water as the sole solvent, while excess linker could be readily removed after synthesis. Alternatively, pre‐neutralizing H_3_BTC with sodium hydroxide to generate the corresponding trisodium trimesate, prior to addition to a Ni(II)/Co(II) metal salt mixture, yielded Ni/Co‐BTC [118].

Beyond linker precursor engineering, waste metal streams have been employed as sustainable metal sources for mono‐, bi‐, and multimetallic MOFs (Figure 8b) [119]. Recently, Zhang et al. reported a room‐temperature, scalable synthesis of ZnFe‐BMOFs using pretreated galvanizing pickling wastewater as a source of Fe(II) and Zn(II) ions, alongside linker salts [120]. This strategy enabled large‐scale production of three distinct BMOFs (>100 g per batch), with a substantial cost reduction (∼91%‒93%) relative to the conventional solvothermal route. Notably, the composition of industrial wastewater varies with its source and treatment history, which can directly affect framework composition, crystallinity, and catalytic performance, as discussed in Section 3.2.2. The reported waste stream also contained Ca, Mn, Al, S, Cl, and Br at concentrations ranging from 0.049 to 55.43 wt%. Moreover, both the small‐ and large‐scale products showed relatively low crystallinity, together with unassigned diffraction peaks that may indicate impurity phases. Therefore, although waste‐derived metal streams provide a promising route toward lower‐cost BMOF production, the scalable preparation of phase‐pure and highly crystalline materials with reproducible composition remains challenging.

Plastic upcycling offers another route for producing functional MOFs and BMOFs while reducing reliance on petrochemical‐derived linkers (Figure 8c) [121, 122, 123, 124, 125]. Polyethylene terephthalate (PET) and poly(butylene adipate‐co‐terephthalate) collected from waste plastic bags have been used as alternative sources of H_2_BDC for the one‐pot solvothermal synthesis of FeCo‐BDC [126, 127]. However, several reported approaches still require toxic organic solvents [126, 127, 128] and elevated temperatures [126, 127], which reduce their overall environmental benefit and limit practical scale‐up. More sustainable depolymerization methods are therefore needed. For example, the alkaline ethanolic depolymerization process reported by Ügdüler et al., conducted at 50°C−80°C under atmospheric conditions [129], offering a potentially milder route for recovering terephthalate monomers. Its direct integration with BMOF synthesis, however, remains largely unexplored.

While various approaches have been reported for the scalable synthesis of monometallic MOFs—including electrochemical, mechanochemical, continuous flow methods [130],—the large‐scale production of BMOFs remains largely unexplored, representing a critical bottleneck toward practical environmental applications.

### 2.4 Purification and Activation

Synthesis of mono‐ and bimetallic MOFs often results in impure materials, with impurities—such as unreacted or decomposed reactants, metal oxides, or recrystallized linkers—present on the surface or trapped within the pores of the synthesized framework. Consequently, thoroughly removing impurities from BMOFs is essential to optimize their catalytic performance. This can be accomplished through both purification and activation processes. Purification, which involves the removal of surface impurities, is typically carried out using solvent treatment at elevated temperatures [20]. Activation, which removes guest molecules trapped within the pores, often produces pure crystalline frameworks with high S.A. and permanent porosity [20, 131, 132].

Purification and activation of BMOFs often involve one or more steps of solvent washing and exchange, as illustrated in Figure 9 [133, 134]. Heat treatment of MOFs containing high‐boiling‐point solvents with high surface tension (e.g., *N,N′*‐dimethylformamide (DMF)) within their pores may cause the framework to collapse. Therefore, it is preferable to exchange the trapped solvent with one of lower boiling point and surface tension, such as methanol (MeOH) [20]. A solvent exchange using supercritical CO_2_ (ScCO_2_) has demonstrated substantial pore evacuation, achieving up to a 1200% increase in N_2_‐accessible S.A [135].

**FIGURE 9:**
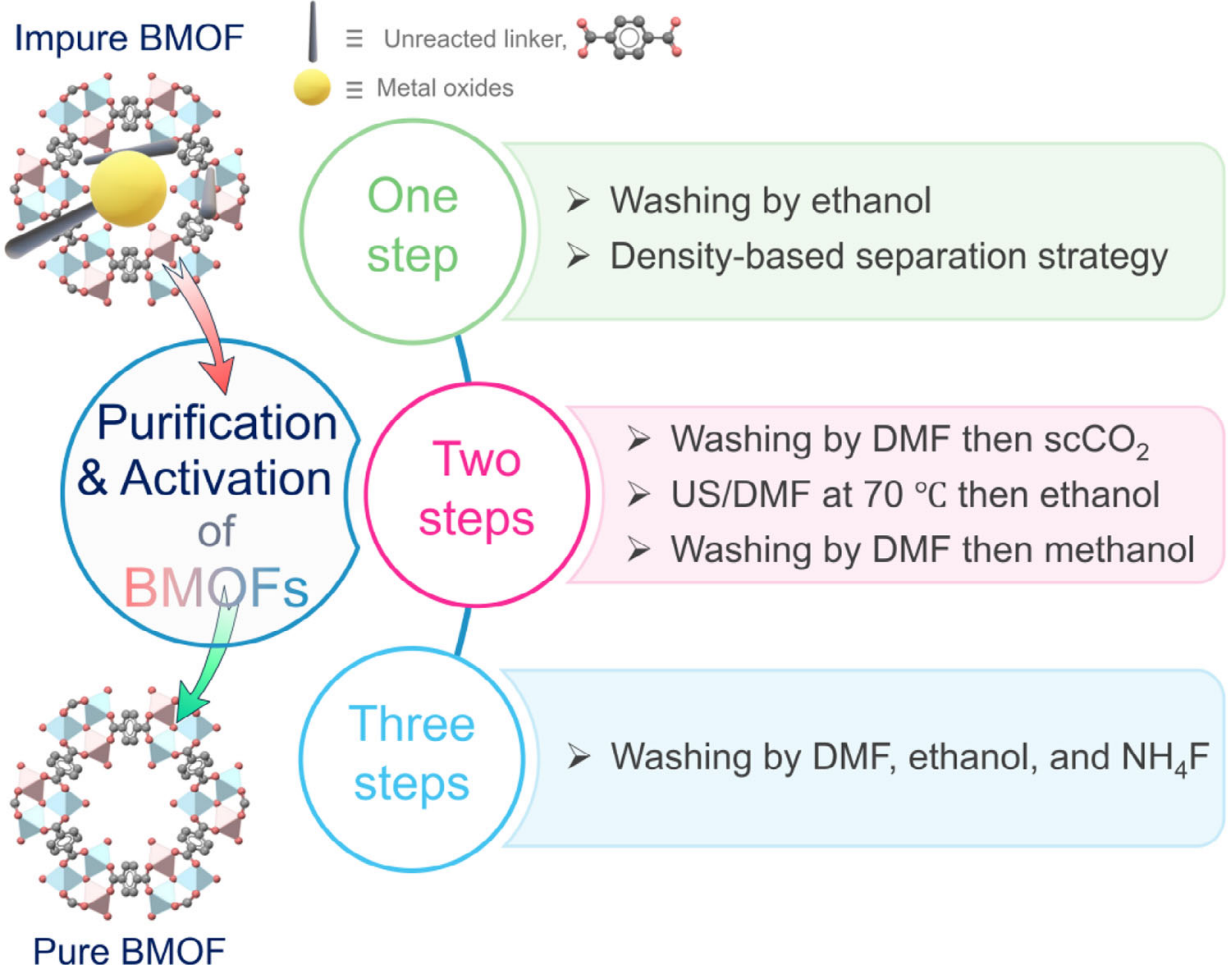
Various purification and activation strategies for BMOFs.

In some cases, simple solvent treatment is insufficient for removing impurities from the pores. Sequential purification steps—washing, solvent exchange, and high‐temperature evacuation—have been used to achieve thorough internal pore evacuation in MIL‐101(Cr, Mg) [136]. A facile purification and activation process for frameworks with low thermal stability (below 100°C) has also been reported, through ultrasonication in different amides at varying temperatures [137].

Farha et al. proposed a density‐based separation strategy, leveraging density differences among MOFs [138]. By utilizing a dense solvent, such as bromochloromethane (CH_2_BrCl, density ≈ 1.93 g cm^−3^) [139]. MOFs float on the solvent's surface. Adding a miscible, lighter solvent (e.g., dimethyl sulfoxide, DMSO, density ≈ 1.1 g cm^−3^) [140] then allows the mixed MOFs to be separated into floating and sinking fractions.

### 2.5 Design Guidelines for BMOF Synthesis

When designing BMOFs for wastewater remediation, hydrolytic stability should be considered first because these materials operate in aqueous, and often strongly oxidative, environments. Carboxylate‐based linkers form stable bonds with high‐valent metals (Al(III), Cr(III), and Fe(III)), yet the catalytically active metal centers in AOPs are predominantly divalent (Co(II), Fe(II), and Cu(II)), resulting in inherently weaker metal–linker bonds and compromised framework stability [77, 78, 79, 80]. For instance, Co‐BDC has been reported to undergo complete dissolution under slightly acidic conditions [5]. One possible strategy to address this challenge is to use bifunctional linkers containing both carboxylate and nitrogen‐donor groups, which may stabilize high‐valent metal centers through carboxylate coordination and divalent metal centers through nitrogen coordination, in line with the HSAB principle. Metals with complementary redox properties are more likely to promote cooperative oxidant activation and redox cycling [141]. Beyond stability, successful OPR requires that both metals exhibit compatible affinities and comparable reactivities toward the linker, ensuring their simultaneous coordination and homogeneous distribution within the resulting framework under the same conditions. When these conditions are not met, PSME and CSBU represent viable alternatives to OPR. Synthetic design should further consider purification and activation. The selected washing, solvent‐exchange, and activation procedures should match the chemical and thermal stability of the as‐synthesized framework to avoid pore collapse or structural degradation (Section 2.4). From an application perspective, scalable and sustainable synthesis is also important. Greener solvents, room‐temperature synthesis, linker salts, and waste‐derived precursors may improve the practical relevance of BMOFs, but these approaches must still deliver adequate crystallinity, phase purity, and stability. Despite these guidelines, MOF/BMOF discovery remains empirical. Emerging data‐driven approaches, including artificial intelligence (AI), machine learning (ML), and large language models (LLMs), may accelerate this process by assisting in the prediction of framework composition, stability, metal combinations, and synthesis conditions [142, 143]. These tools can further assist in optimizing metal precursor ratios and predicting the physicochemical properties of BMOFs prior to synthesis [144]. The proposed design considerations are summarized in Figure 10.

**FIGURE 10:**
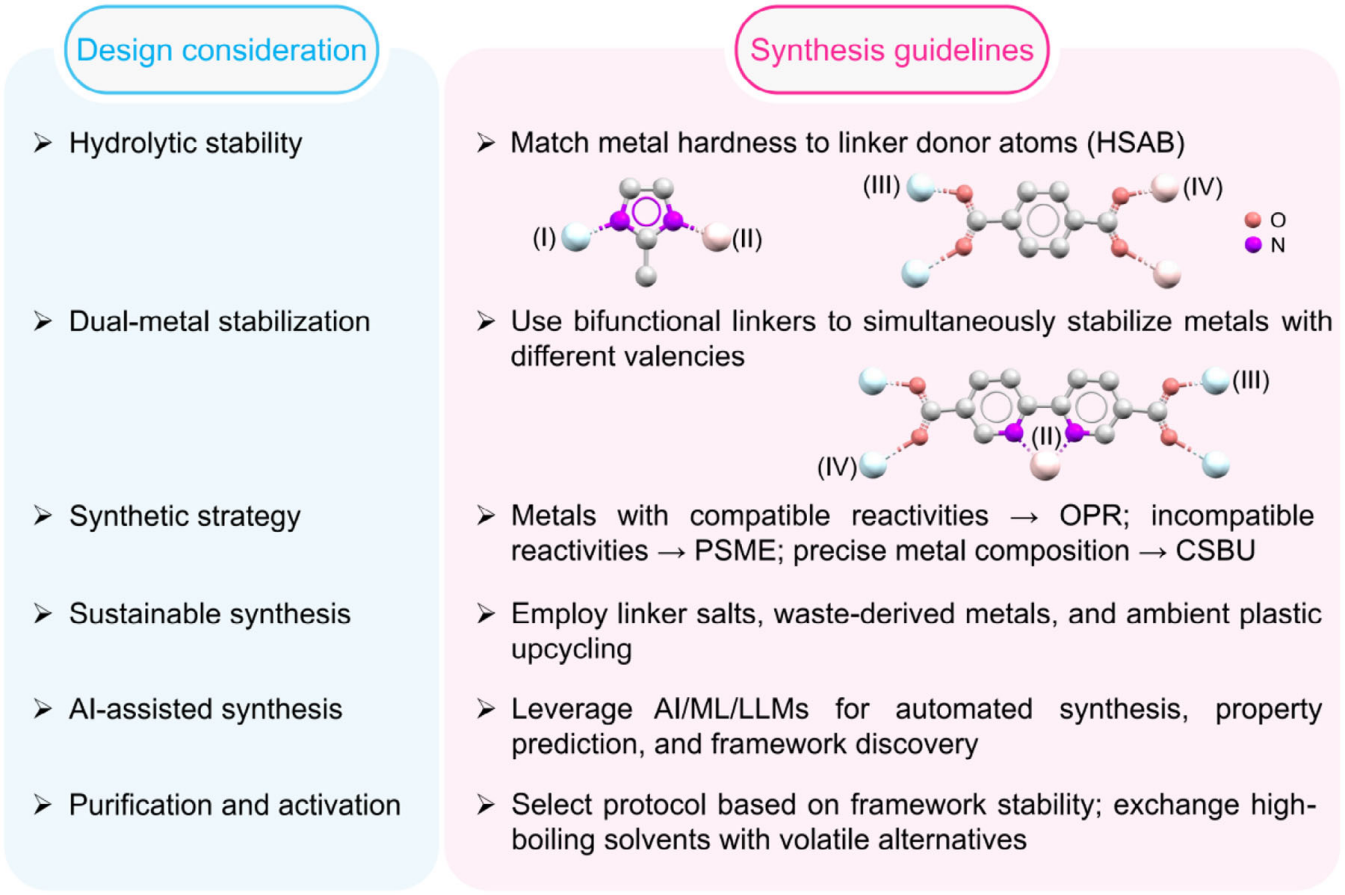
Design guidelines for BMOF synthesis.

### 2.6 Characterization and Validation of BMOF Formation

Given the inherent competition between two metal species for coordination with the organic linker—which may result in phase separation or the formation of mixed monometallic MOFs rather than a true bimetallic framework (Section 2.1.1)—a systematic, multi‐technique characterization and validation workflow is essential to unambiguously validate successful BMOF formation (Figure 11).

**FIGURE 11:**
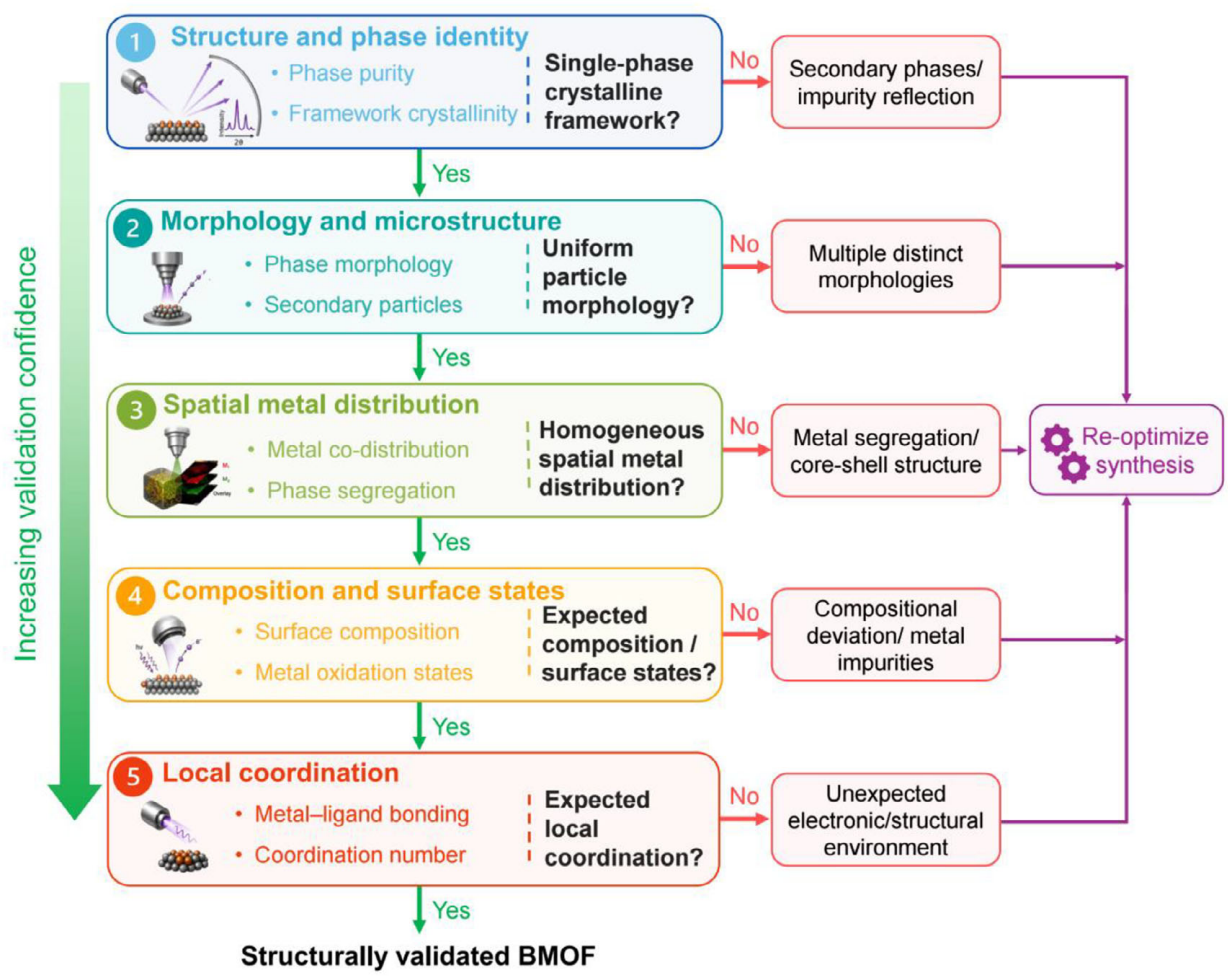
Integrated multi‐technique characterization workflow and decision tree for the systematic structural validation of BMOFs.

Powder X‐ray diffraction (PXRD) is the primary tool for assessing the phase purity of as‐synthesized BMOFs, wherein experimental diffraction patterns are compared against simulated patterns of the parent monometallic frameworks to confirm single‐phase formation and rule out co‐crystallization of competing monometallic phases. Under partial isomorphous substitution, the BMOF pattern may closely resemble that of the parent framework, as in CuCo‐BDC, where Co incorporation preserved the characteristic diffraction peaks of the parent Cu‐BDC [5]. Conversely, the coexistence of peaks characteristic of both parent monometallic frameworks suggests either a physical mixture of monometallic phases or an impure BMOF [145]. At higher doping concentrations, progressive peak broadening and reduced crystallinity may occur [146], underscoring the importance of careful doping optimization. Systematic peak shifts or the emergence of entirely new diffraction patterns may reflect more substantial perturbations to the crystal lattice [147, 148]. In such cases, theoretical calculations and AI‐assisted structure prediction tools can resolve complex crystal structures and interpret experimental diffraction data [143]. The challenge is further compounded when the constituent monometallic MOFs share identical topology, space group, and nearly identical lattice parameters—as in ZIF‐8 and ZIF‐67—rendering the BMOF pattern virtually indistinguishable from a physical mixture of the monometallic constituents [149, 150, 151]. Distinguishing a true BMOF from a physical mixture or core‐shell structure therefore requires additional techniques—such as electron microscopy [152], extended X‐ray absorption fine structure (EXAFS), and neutron powder diffraction [65],—capable of probing the spatial arrangement of metals within the SBUs.

Following PXRD analysis, electron microscopy—encompassing scanning electron microscopy (SEM) and transmission electron microscopy (TEM)—provides complementary morphological evidence of BMOF formation. A phase‐pure BMOF is generally expected to exhibit a uniform, well‐defined single‐particle morphology, whereas the coexistence of multiple distinct morphologies suggests the presence of mixed monometallic phases or an impure BMOF [145, 152]. PXRD should therefore always be interpreted in conjunction with SEM/TEM to reliably validate BMOF phase purity [152].

Energy‐dispersive X‐ray (EDX) elemental mapping provides spatial distribution information for both metal species within the framework, where homogeneous co‐distribution supports true bimetallic integration [149], rather than phase segregation or core‐shell architecture [151]. However, owing to its inherent sensitivity limitations, EDX is unsuitable for precise quantification of metal content, particularly at low doping concentrations.

X‐ray photoelectron spectroscopy (XPS) survey spectra confirm the elemental composition of as‐synthesized BMOFs; however, detection of low‐concentration dopants may be hindered by the inherent detection limit of the technique [153]. Because XPS oxidation‐state assignments often underpin the proposed redox mechanisms in the BMOF–AOP literature, they merit particularly careful interpretation. Assignments benefit from close alignment with established reference positions [154, 155], and from peak fits that track the raw spectra closely [156, 157, 158, 159, 160, 161]. Since the proposed redox mechanism rests on these oxidation states, additional justification would further strengthen the mechanistic interpretation.

X‐ray absorption spectroscopy (XAS) provides deeper insight into the local electronic and structural environment of each metal center: X‐ray absorption near‐edge structure (XANES) enables precise oxidation‐state determination even at very low doping concentrations, while EXAFS reveals the coordination environment, bond lengths, and coordination numbers of each metal within the framework [64].

Finally, inductively coupled plasma (ICP) analysis enables accurate bulk quantification of metal content in the digested framework, providing the actual metal ratios incorporated and confirming the fidelity of the target bimetallic composition.

## 3 BMOF‐AOPs for Wastewater Remediation

BMOFs have emerged as promising materials for advanced water treatment, leveraging the structural versatility of MOFs alongside the synergistic interactions between distinct metals. Careful selection of the two metals allows BMOFs to exhibit exceptional catalytic performance in various water purification strategies. This section explores various BMOF‐AOPs for water decontamination, including photocatalytic degradation, heterogeneous Fenton catalysis (HFC), sulfate radical generation (SRG), catalytic ozonation (CA‐O_3_), and PAA activation.

Demonstrating true bimetallic synergy requires systematic experimental validation rather than relying only on enhanced catalytic activity. A hierarchical approach is therefore proposed (Figure 12): (i) The BMOF should be compared with both monometallic counterparts under identical reaction conditions and equivalent catalyst or active‐metal loadings. Outperforming both monometallic materials provides initial evidence of enhancement but does not, by itself, confirm synergy; (ii) the BMOF should outperform a physical mixture of M_1_‐MOF and M_2_‐MOF, at equivalent molar ratios and under the same conditions [162], confirming cooperative rather than merely additive contributions; (iii) leaching controls and hot filtration tests must rule out homogeneous contributions from dissolved metal ions; (iv) spectroscopic evidence—including XPS oxidation‐state changes confirming redox cycling and electron paramagnetic resonance (EPR) measurements identifying reactive species—provides mechanistic support for the proposed synergistic pathways; and (v) computational investigations, such as density functional theory (DFT) calculations of intermetallic charge transfer and activation energy barriers, provide atomistic insight into the electronic structure and synergistic interactions.

**FIGURE 12:**
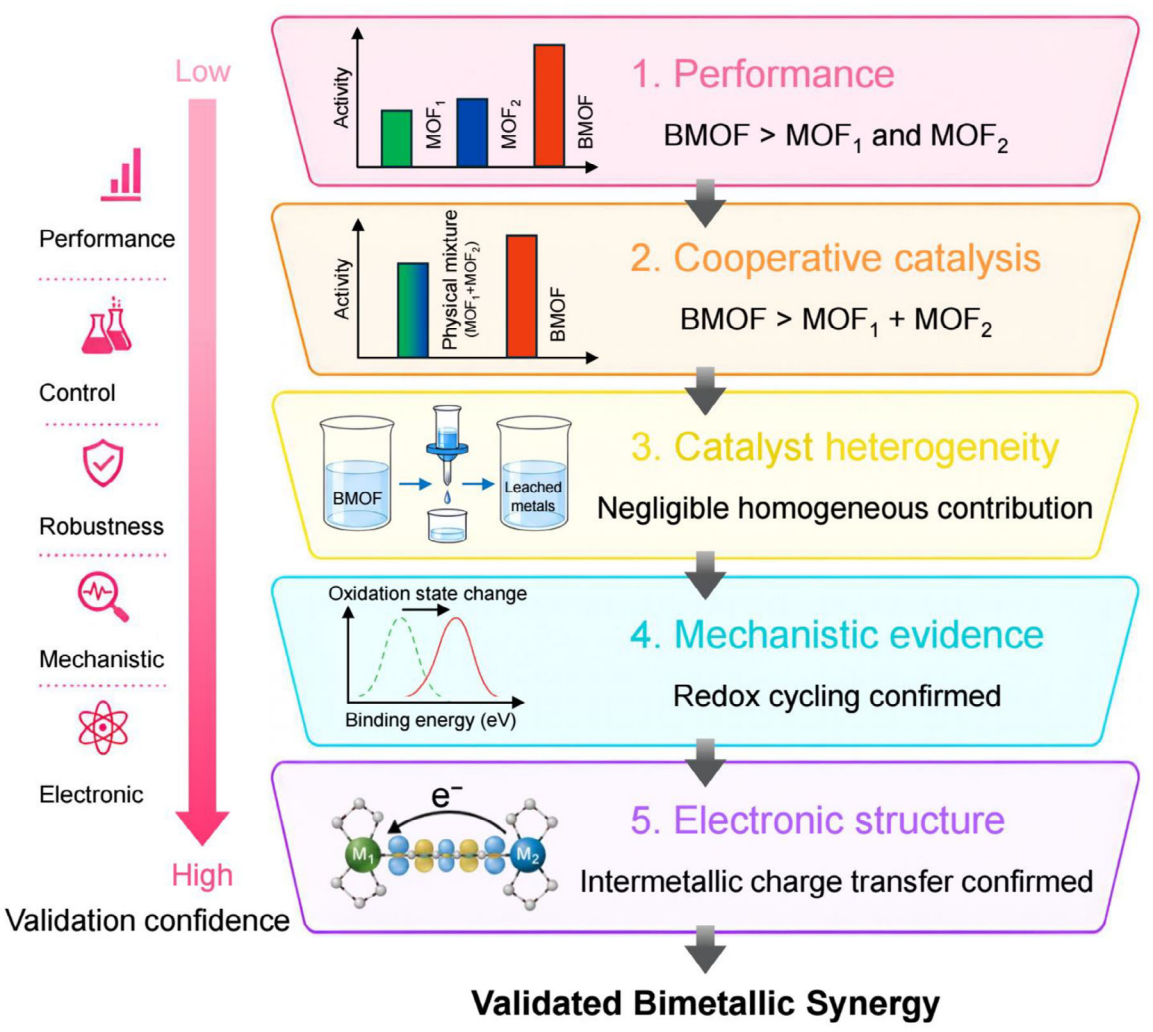
Hierarchical validation framework for true bimetallic synergy in BMOFs.

Unless otherwise stated, quantitative performance comparisons in this review are limited to materials evaluated within the same study under identical experimental conditions. Direct comparison across different studies should be treated with caution because the reported degradation efficiency and apparent rate constant depend strongly on pollutant identity and concentration, catalyst dosage, oxidant concentration, pH, light source and intensity, reaction volume, temperature, water matrix, and reactor configuration. To provide additional context, the available experimental conditions are listed in Tables 1, 2, 3. The mass‐normalized rate constant (*k_nor_*; min^−1^ g^−1^ L) is also reported as a supporting descriptor (Equation 1): [163].

(1) $$k_{\mathrm{nor}}=k_{\mathrm{obs}}/m_{\mathrm{cat}}$$

where *k*_obs_ is the observed pseudo‐first‐order rate constant (min^−1^), and *m*_cat_ is the catalyst dosage (g L^−1^).

**TABLE 1:** Reported BMOFs for photocatalytic wastewater depollution.

Compound	Synthesis conditions	Concept	Application	Targeted pollutants	Reaction conditions	*k*_nor_ ^*^ (min^−1^ g^−1^ L) (MOF; BMOF)	Metal leaching (mg L^−1^)	Mechanism	Refs.
Ti/Zr‐NH_2_‐MIL‐125	Solvothermal (150°C/16 h)	Degradation under simulated solar irradiation.	Degradation	ACE (100%, 3 h)	[Pollutant] ≈ 5 mg L^−1^, dose = 0.25 g L^−1^, irradiatio*n* = 600 W m^−2^ Xe lamp equipped with a 290 nm cutoff filter, *T* = 20°C, and pH = 6.9	(0.0292; 0.0486)	N/A	ACE photodegradation is dominated by O_2_^•−^ generated from photogenerated electrons and dissolved O_2_, with minor contribution from ^•^OH and electrons.	[146]
In/Fe‐NH_2_‐MIL‐68	Solvothermal (125°C/5 h)	Degradation under visible light irradiation	Reduction	Cr(VI) (99.3%, 120 min)	[Pollutant] = 20 mg L^−1^, *V* = 50 mL, dose = 20 mg, irradiation = 300 W Xe lamp equipped with a 420 nm cutoff filter, and pH = 2.	(0.0045; 0.09)	N/A	Upon irradiation, LMCT generates electron‐hole pairs; CB electrons reduce Cr(VI), while VB holes oxidize TC‐HCl to CO_2_ and H_2_O.	[171]
			Oxidation	TC‐HCl (71.5%, 120 min)	[Pollutant] = 20 mg L^−1^, *V* = 50 mL, dose = 0.2 g L^−1^, and pH = 9	(0.0135; 0.045)	N/A		
Co/Fe‐MIL‐53‐NH‐BT, BT: Benzothiadiazole	Solvothermal (120°C/24 h)	Degradation under visible light irradiation	Degradation	BPA (99.9%, 120 min), OFX (99.8%, 120 min), MO (95.4%, 80 min) MB (99%, 80 min)	[Pollutant] = 10 mg L^−1^, *V* = 20 mL, dose = 0.5 g L^−1^, irradiatio*n* = 500 W m^−2^ Xe lamp equipped with a 400 nm cutoff filter, and pH = 5	(0.048; 0.079)	N/A	Upon visible light excitation, charge separation generates ROS, predominantly ^•^OH, O_2_^•−^, and h^+^, which drive oxidative pollutant degradation.	[180]
Fe/Ce‐BDC‐NH_2_	Solvothermal (180°C/24 h)	Solar photocatalytic degradation	Degradation	PCM (94.6%, 270 min)	[Pollutant] = 10 mg L^−1^, *V* = 100 mL, dose = 0.1 g L^−1^, and pH = 5.6	(0.038; 0.138)	N/A	LMCT to Fe and Ce, with Ce accepting electrons from Fe, generates ^•^OH, while the ligand acts as a hole sink; radical and holes drive pollutant degradation.	[182]
Fe/Al‐BDC	Solvothermal (200°C/70 h)	Batch adsorption	Adsorption	F^−^ (95.6%, 45 min)	[Pollutant] = 10 mg L^−1^, *V* = 100 mL, dose = 0.1 g L^−1^, pH = 7, and *T* = 30°C		N/A	Adsorption follows surface boundary‐layer, intraparticle diffusion model, and mass transfer mechanisms.	[188]
		Degradation under sunlight	Degradation	RhB and MB (<90%)	[Pollutant] = 10^−4^ and 10^−5^ mol L^−1^, *V* = 100 mL, dose = 0.1 g L^−1^, pH = 7, and *T* = 30°C	Two‐step kinetic	N/A	Photodegradation is dominated by ^•^OH attack on dye molecules.	
Co/Cu‐CAT‐1	Solvothermal (95°C/18 h)	Photocatalytic‐photothermal effect	Bacterial inactivation	*E. coli*	1 mg of catalyst, 15 mL of 1.5 × 10^8^ cfu mL^−1^ *E. coli* solution, 300 rpm, and 300 W Xe lamp (4.5 kW m^−2^) equipped with a 420 nm cutoff filter	N/A	N/A	Synergistic photocatalytic‐photothermal inactivation arises from thermal effects, intrinsic antibacterial activity of Co and Cu, and oxidative attack by h^+^ and ^•^OH.	[40]
Zr/Ce‐BDC‐NH_2_	Solvothermal (120°C/24 h)	Degradation under visible light irradiation	Degradation	CIP (90.8%, 60 min)	[Pollutant] = 20 mg L^−1^, *V* = 20 mL, irradiatio*n* = 300 W Xe arc lamp equipped with a 420 nm cutoff filter, and dose = 1 g L^−1^	(0.0094; 0.0363)	N/A	CIP adsorbs on BMOF, and upon light irradiation, electron–hole pairs generate ^•^OH and O_2_^•−^, along with h^+^ and electrons, oxidatively mineralize the adsorbed CIP.	[184]
Ti/In‐H_4_TBAPy^*^	Solvothermal (150°C/24 h)	Degradation under visible light irradiation	Degradation	BPA (100%, 20 min)	[Pollutant] = 50 mg L^−1^, *V* = 20 mL, irradiatio*n* = 300 W Xe lamp (520 mW cm^−2^) equipped with a 420 nm cutoff filter, and dose = 0.25 g L^−1^	(0.096; 0.876)	Ti (15.2), In (27.3)	Removal results from synergistic adsorption, which accelerates pollutant access to ROS‐active sites, and ROS‐driven degradation upon light irradiation.	[48]
Ti/Zr‐BDC‐NH_2_	Solvothermal (150°C/15 h)	Degradation under visible light irradiation	Degradation	CBZ (80%, 60 min), BP‐2 (95%, 60 min), BPA (90%, 60 min)	[Pollutant] = 20 µmol L^−1^, irradiation = 300 W Xe lamp (100 mW cm^−2^), and dose = 0.2 g L^−1^	(0.01; 0.0665)	N/A	Removal arises from adsorption and degradation, both enhanced by charge redistribution after Zr doping.	[45]
Fe/Ni‐BDC	Solvothermal (110°C/20 h)	Degradation under natural sunlight	Degradation	RhB (91%, 120 min), CR (89%, 120 min)	[Pollutant] = 10 mg L^−1^, V = 100 mL, irradiation = natural sunlight (94 ± 8 mW cm^−2^), dose = 0.2 g L^−1^, pH = 7	N/A	N/A	Dye molecules preferentially adsorb on the Ni centers, followed by degradation via photogenerated ^•^OH and O_2_^•−^.	[152]

* 4,4′,4″,4″′‐(pyrene‐1,3,6,8‐tetrayl) tetra‐benzoic acid.

Abbreviations: ACE, acetaminophen; BP‐2, tetrehydroxybenzophenone; BPA, bisphenol A; CBZ, carbamazepine; CIP, ciprofloxacin; CR, Congo red; *E. coli*, *Escherichia coli*; *k_nor_*, mass‐normalized rate constant; MB, methylene blue; MO, methyl orange; N/A, not available; OFX, ofloxacin; PCM, paracetamol; RhB, rhodamine B; TH‐HCl, tetracycline hydrochloride.

**TABLE 2:** Reported BMOFs for wastewater treatment via heterogeneous Fenton catalysis.

Compound	Synthesis conditions	Concept	Pollutant	Reaction conditions	k_nor__*_ min^−1^ g^−1^ L (MOF; BMOF)	Metal leaching mg L^−1^	Mechanism	Refs.
Fe(II)‐MIL‐53(Fe)	Solvothermal (150°C/3 h)	H_2_O_2_ activation	4‐NP (95.2%, 120 min)	[Pollutant] = 20 mg L^−1^, *V* = 50 mL, dose = 0.4 g L^−1^, pH = 3–4, and [H_2_O_2_] = 10 mM	(0.0275; 0.0625)	Fe (0.1–∼0.6)	4‐NP adsorbs on BMOF, and Fe(II), CUMSs, and low electron density Fe(III) generate surface ^•^OH driving degradation.	[43]
Cu/Fe‐BDC	Solvothermal (110°C/36 h)	H_2_O_2_ activation	SMX (100%, 120 min)	[Pollutant] = 20 mg L^−1^, V = 80 mL, dose = 0.5 g L^−1^, pH = 5.6, and [H_2_O_2_] = 6 mM	NA	Fe (7.23), Cu (0.11)	SMX adsorbs on BMOF, and metal sites synergistically activate H_2_O_2_ to produce surface‐bound ^•^OH, which oxidizes the adjacent SMX.	[46]
Cu/Fe‐MIL‐101 (CUMSs)	Solvothermal (110°C/20 h)	H_2_O_2_ activation	CIP (100%, 30 min) BPA, phenol, RhB, and MB (80%−100%, 20 min)	[Pollutant] = 20 mg L^−1^, *V* = 100 mL, dose = 0.1 g L^−1^, pH = 7, and [H_2_O_2_] = 3 mM	(0.34; 2)	Fe (0.36), Cu (0.72)	CIP adsorbs on BMOF and electron transfers from CIP to electron‐deficient centers of BMOF, causing self‐oxidative degradation. Additionally, BMOF/H_2_O_2_ then forms ^•^OH, causing oxidative mineralization.	[205]
Cu/Fe‐BUC‐96	MW (400 W) (110°C/1.5 h)	H_2_O_2_ activation	CQ (100%, 5 min)	[Pollutant] = 20 mg L^−1^, *V* = 50 mL, dose = 0.2 g L^−1^, pH = 5.5, and [H_2_O_2_] = 3.9 mM	(0.336; 6.7635)	Fe (1.00), Cu (0.05)	BUC‐96(FeCu)/H_2_O_2_ produces ^•^OH, O_2_^•−^, and ^1^O_2_, which oxidize CQ.	[210]
Cu/Fe‐BDC‐Br	Solvothermal (100°C/12 h)	H_2_O_2_ activation	Phenol (94%, 60 min)	[Pollutant] = 100 mg L^−1^, V = 100 mL, dose = 0.2 g L^−1^, pH = 4, and [H_2_O_2_] = 8 mM	(0.33; 1.06)	Fe (1.78), Cu (0.85)	Cu doping and Br functionalization boost Fe(III)/Fe(II) redox cycling, generating ^•^OH, O_2_^•−^, ^1^O_2_, and electrons for degradation.	[212]
Co/Fe‐MIL‐101	Solvothermal (110°C/20 h)	H_2_O_2_ activation	CIP (97.8%, 30 min)	[Pollutant] = 20 mg L^−1^, V = 100 mL, dose = 0.2 g L^−1^, pH = 5, and [H_2_O_2_] = 5 mM	(0.08; 0.6)	Fe (1.01), Co (0.23)	CIP transfers electrons to BMOF, triggering self‐oxidation, while electron‐rich metal sites activate H_2_O_2_ for further oxidative degradation.	[206]
Co/Fe‐BTC (CUMSs)	Solvothermal (170°C/10 h)	H_2_O_2_ activation	AMX (100%, 30 min)	[Pollutant] = 10 mg L^−1^, V = 50 mL, dose = 0.2 g L^−1^, pH = 7, and [H_2_O_2_] = 3 mM	(4.64; 7.32)	Fe (0.29), Co (0.61)	Electron transfers from CIP to BMOF, and BMOF/H_2_O_2_ generates ^•^OH, causing oxidative mineralization of CIP.	[214]
		Disc diffusion	Antibacterial activity: *E. coli* (I.Z.: 25.21 mm), *S. aureus* (I.Z.: 19.51 mm)	10^6^ bacterial suspension spread			NA	
Co/Fe‐BDC (CUMSs)	MW‐assisted solvothermal, 500 W (140°C/20 min)	H_2_O_2_ activation	OTC (82%, 60 min; 100%, 90 min)	[Pollutant] = 50 mg L^−1^, dose = 0.125 g L^−1^, pH = 8, and [H_2_O_2_] = 20 mM	NA	Fe (1), Co (0.8)	Degradation of OTC is driven primarily by surface‐bound ^•^OH.	[213]
Mn/Fe(II)‐MIL‐101	Room temperature/24h	H_2_O_2_ activation	Phenol (100%, 20 min)	[Pollutant] = 150 mg L^−1^, *V* = 50 mL, dose = 0.1 g L^−1^, pH = 6, and [H_2_O_2_] = 20 mM	(1.21; 3.77)	Fe (4.0), Mn (0.6)	Adsorbed phenol transfers electrons to BMOF; H_2_O_2_ adsorption and activation by electron‐rich sites forms ^•^OH and O_2_^•−^, oxidizing phenol.	[215]
M/Fe‐LC‐MIL‐53, LC: low crystalline, M = Mn(II) or Cu(II)	Solvothermal (150°C/5 h)	Photo‐Fenton degradation	CIP (Mn: 89%, 30 min), (Cu: 61%, 30 min)	[Pollutant] = 20 mg L^−1^, *V* = 100 mL, dose = 0.1 g L^−1^, pH = 7, [H_2_O_2_] = 5 mM, and irradiation = 300 W Xe lamp equipped with a 420 nm cutoff filter	(0.16; 0.763)	Fe (0.15) Mn (undetectable)	H_2_O_2_ adsorbs on CUMSs in LC‐BMOF; light‐induced LMCT to Fe(III) generates Fe(II), activating adsorbed H_2_O_2_ to produce ^•^OH.	[207]
Fe/Ni‐BDC	Solvothermal (150°C/12 h)	Photo‐Fenton degradation	MB (≈100%, 40 min), MO (≈93%, 75 min)	[Pollutant] = 20 mg L^−1^, *V* = 100 mL, dose = 0.1 g L^−1^, pH = 6.5–6.9, [H_2_O_2_] = 10 mL L^−1^, and irradiation = 300 W Xe lamp equipped with a 420 nm cutoff filter	NA	Fe (0.4– ∼0.6)	Dye molecules adsorb on BMOF via π–π interactions, enhanced by the enlarged surface area; light excitation followed by H_2_O_2_ activation and oxidative degradation.	[35]
Fe(II)/Fe(III)‐MIL‐100 (CUMSs)	Solvothermal (150°C/24 h), followed by vacuum activation at 150–250°C for 12 h	Photo‐Fenton degradation	TC‐HCl (≈95%, 80 min)	[Pollutant] = 50 mg L^−1^, *V* = 100 mL, dose = 0.2 g L^−1^, pH = 4, [H_2_O_2_] = 20 mL L^−1^, and irradiation = 300 W Xe lamp equipped with a UV‐cutoff filter	(0.06; 0.19)	Fe (∼0.45)	H_2_O_2_ adsorbs on Lewis‐acid CUMSs generating ^•^OH. In addition, photogenerated electrons reduce O_2_ to O_2_^•−^, oxidizing the pollutant.	[109]
Fe/Zn‐BMOFs (Linker = BDC, BTC, and Fum)	Room temperature/24 h	Photo‐Fenton degradation	TC‐HCl (100%, 40 min)	[Pollutant] = 20 mg L^−1^, *V* = 100 mL, dose = 0.1 g L^−1^, pH = 3, [H_2_O_2_] = 3 mM, and irradiation = 300 W Xe lamp	(0.29; 1.33)	Fe (2), Zn (0.22)	^•^OH, O_2_^•−^, and ^1^O_2_	[120]

Abbreviations: 4‐NP, 4‐nitrophenol; AMX, amoxicillin; CQ, chloroquine phosphate; Fum, fumarate; NA, not available; OTC, oxytetracycline; SMX, sulfamethoxazole.

**TABLE 3:** Reported BMOFs for wastewater treatment via sulfate radical generation, catalytic ozonation, and peracetic acid (PAA) activation.

Compound	Synthesis conditions	Oxidant	Pollutant	Reaction conditions	*k*_nor*_ min^−1^ g^−1^ L (MOF; BMOF)	Metal leaching mg L^−1^	Mechanism and ROS	Refs.
Fe(II)/Co‐BDC	Solvothermal (150°C/24 h)	PMS	Phen (96.5%, 30 min)	[Pollutant] = 1 mg L^−1^, dose = 0.05 g L^−1^, pH = 3.15, and [PMS] = 0.2 mM	(N/A; 2.68)	Co (3.78), Fe (3.19)	SO_4_^•−^, ^•^OH, and O_2_^•−^	[160]
Fe(II)/Co‐BDC‐NH_2_	Solvothermal (100°C/48 h)	PMS	Organic pollutants (100%, 60 min)	[Pollutant] = 0.2 mM, *V* = 99 mL, dose = 0.05 g L^−1^, and [PMS] = 2 mM	(1.008; 1.422)	Co (1.32), Fe (1.42)	SO_4_^•−^, ^•^OH, and ^1^O_2_, with a major contribution of the singlet oxygen. Amino groups accelerate electron transfer from BMOF to PMS.	[47]
Fe/Co‐MIL‐101	Solvothermal (120°C/12 h)	PMS	Organic pollutants (99%, 15 min)	[Pollutant] = 10 mg L^−1^, *V* = 20 mL, dose = 0.2 g L^−1^, pH = 7, and [PMS] = 1.3 mM	(0.0285; 1.003)	Co (0.7), Fe (1.06)	SO_4_^•−^ and ^•^OH. Upon PMS activation, Fe(III) and Co(II) are converted into Fe(II) and Co(III), respectively.	[38]
2D Fe/Co‐BDC	Room temperature (12 h)	PMS	RhB (99%, 5 min)	[Pollutant] = 20 mg L^−1^, V = 100 mL, dose = 0.02 g L^−1^, pH = 4.68, and [PMS] = 0.25 mM	(25.55; 44.2)	N/A	O_2_^•−^ and ^1^O_2_ are the dominant species, while SO_4_^•−^ and ^•^OH contributed minimally.	[232]
Fe/Co‐ZIF	Room temperature (4 h)	PMS	TC (96.7%, 5 min)	[Pollutant] = 30 mg L^−1^, *V* = 100 mL, dose = 0.2 g L^−1^, pH = 4.21, and [PMS] = 1 mM	N/A	N/A	SO_4_^•−^ and ^1^O_2_	[233]
Cu/Co‐DHTA (Cu/Co‐MOF‐74)	Solvothermal (150°C/24 h)	PMS	MB (100%, 30 min)	[Pollutant] = 0.2 mM, *V* = 100 mL, dose = 0.05 g L^−1^, and [PMS] = 2 mM	(N/A; 2.96)	Co (1.3), Cu (1.2)	SO_4_^•−^, ^•^OH, and ^1^O_2_. Cu(II) and Co(II) are converted into Cu(I) and Co(III), respectively.	[239]
Cu/Co‐HHTP	Solvothermal (80°C/12 h)	PMS	NIM (100%, 25 min)	[Pollutant] = 20 mg L^−1^, *V* = 100 mL, dose = 0.2 g L^−1^, pH = 5.4, and [PMS] = 3 mM	(0.3; 1.135)	Co (0.07) Cu (0.13)	Co doping enhances electron transfer from organic linkers to Cu, increasing PMS adsorption and O‐O bond length, efficiently generating O_2_^•−^, ^1^O_2_, SO_4_^•−^, and ^•^ OH.	[240]
2D CuCo‐BDC	Room temperature; 24h	PMS	NPX (100%, 20 min)	[Pollutant] = 20 mg L^−1^, V = 40 mL, dose = 0.25 g L^−1^, pH = 4.1, and [PMS] = 0.25 mM	(0; 1.156)	Co (0.16–0.95)	The 2D morphology, mesoporous architecture, and role of Cu(I) to reduce Co(III) enhance PMS activation, efficiently generating O_2_^•−^, ^1^O_2_, SO_4_^•−^, and ^•^ OH.	[5]
Nb/Co‐BDC‐NH_2_	Solvothermal (180°C/96 h)	PMS	TC (96.9%, 10 min)	[Pollutant] = 20 mg L^−1^, *V* = 100 mL, dose = 0.2 g L^−1^, pH = 6.02, and [PMS] = 1 mM	(4.5 (homogeneous); 1.78)	Co (0.866), Nb (N/A)	SO_4_^•−^, ^•^OH, O_2_^•−^, and ^1^O_2_	[244]
Mn/Co‐CHXhc	Hydrothermal (180°C/24 h)	PMS	RhB (100%, 25 min)	[Pollutant] = 10 mg L^−1^, V = 50 mL, dose = 0.2 g L^−1^, pH = 4.8, and [PMS] = 0.2 mM	(N/A; 1.153)	N/A	SO_4_^•−^, ^•^OH, O_2_^•−^, and ^1^O_2_	[246]
Fe(II)/Cu‐BDC‐NH_2_	Solvothermal (90°C/20 h)	PMS	RhB (81%, 90 min)	[Pollutant] = 10 mg L^−1^, V = 50 mL, dose = 1.2 g L^−1^, and [PMS] = 2.5 mM	N/A	Cu (0.10–0.18), Fe (0.050–0.093)	SO_4_^•−^, ^•^OH, and O_2_^•−^	[247]
Fe(II)/Co‐DHTA	Solvothermal (170°C/24 h)	PMS	Phen (100%, 30 min)	[Pollutant] = 1 m g L^−1^, dose = 0.05 g L^−1^, pH = 3.15, and [PMS] = [PDS] = 0.2 mM	PMS (NA; 2.48)	Fe (3.05), Co (2.1)	PMS activation generates SO_4_^•−^, ^•^OH, and ^1^O_2_ PDS activation generates SO_4_^•−^ and ^•^OH	[238]
		PDS	Phen (34.6%, 30 min)					
Cu/Fe‐MIL‐53	MW‐assisted solvothermal 1000 W (170°C/30 min)	PDS	NOF (95.5%, 60 min)	[Pollutant] = 20 mg L^−1^, *V* = 50 mL, dose = 0.4 g L^−1^, pH = 6.55, and [PDS] = 0.2 g L^−1^	(1: 11.45)^a^	N/A	Fe(II) and Cu(I) activate PDS to form surface‐bound SO_4_^•−^ and ^•^OH, degrading NOF. In addition, Cu(I) reduces Fe(III) to Fe(II).	[254]
Co/Ni‐Fc	Solvothermal (120°C/12 h)	PDS	BPA (98%, 5 min)	[Pollutant] = 10 mg L^−1^, *V* = 50 mL, dose = 0.2 g L^−1^, and [PDS] = 0.2 mM	(3.55: 4.95)	Co (1.9), Ni (1.3), Fe (1.2)	The cooperative activation of PDS by Co(II) and Ni(II) generates an intense flux of O_2_^•−^ and ^1^O_2_	[161]
Co/Fe‐BDC‐NH_2_	Solvothermal (110°C/24 h)	PDS	TC (99%, 150 min)	[Pollutant] = 50 mg L^−1^, *V* = 100 mL, dose = 0.2 g L^−1^, and [PDS] = 5 mM	(0.085; 0.165)	N/A	Both Fe(III) and Co(II) synergistically activate PDS and produce O_2_^•−^ and ^1^O_2_, as major species, with minor SO_4_^•−^ and ^•^OH.	[39]
Cu/Fe‐MIL‐101	Solvothermal (110°C/20 h)	PDS/vis light	TC (90.5%, 120 min)	[Pollutant] = 50 mg L^−1^, dose = 0.05 g L^−1^, pH = 5, [PDS] = 6 mM, and irradiation = 300 W Xe lamp	(N/A; 0.321)	N/A	Both PDS and photogenerated electrons produce SO_4_^•−^, ^•^OH, O_2_^•−^, and h^+^.	[41]
Fe/Ni‐BDC	Solvothermal (150°C/3 h)	PDS/vis light	TC (100%, 60 min)	[Pollutant] = 30 mg L^−1^, *V* = 50 mL, dose = 0.2 g L^−1^, [PDS] = 1.5 mM, and irradiation = 300 W Xe lamp (98 mW cm^−2^) combined with a 420 nm cutoff filter	(0.025; 0.375)	Fe (undetectable), Ni (undetectable)	PDS is activated via Fe(II) electron transfer and photocatalysis, generating SO_4_^•−^ and ^•^OH.	[241]
Fe/Zr‐UiO‐66	Solvothermal (PSME) (100°C/24 h)	PDS/vis light	SMTR (89%, 300 min)	[Pollutant] = 10 mg L^−1^, *V* = 100 mL, dose = 0.2 g L^−1^, pH = 6.3, [PDS] = 2 mM, and irradiation = simulated sunlight (720 W m^−2^; 300–800 nm)	N/A	N/A	Photogenerated electrons generate O_2_^•−^, while ligand‐to‐Fe LMCT drives PDS activation, generating SO_4_^•−^ and ^•^OH,	[258]
Fe/Ti‐BDC‐NH_2_	Solvothermal (150°C/48 h)	PDS/vis light	AO7 (100%, 10 min)	[Pollutant] = 50 mg L^−1^, dose = 0.1 g L^−1^, pH = 5, [PDS] = 14 mM, and irradiation = 300 W Xe lamp combined with a 420 nm cutoff filter	(N/A; 2.3)	N/A	Photoelectrons activate PDS to form SO_4_^•−^, or reduce O_2_ to O_2_^•−^; ligand‐to‐Fe(III) LMCT generates Fe(II), further activating PDS.	[259]
Co/Ni‐PMA	Hydrothermal (110°C/72 h)	O_3_	ATZ (93.9%, 20 min)	[Pollutant] = 10 mg L^−1^, *V* = 200 mL, dose = 0.5 g L^−1^, [O_3_] = 8 mg L^−1^, and flow rate = 1 L min^−1^	(0.266; 0.576)	Co (1.21–1.42), Ni (1.24–1.46)	Ozone activation by surface –OH and CUMSs generates ^•^OH, O_2_^•−^, and ^1^O_2_. Also, H_2_O_2_ formation may improve degradation.	[72]
Fe/Ce‐MIL‐88A	Hydrothermal (70°C/12 h)	O_3_	SA (100%, 30 min)	[Pollutant] = 100 mg L^−1^, *V* = 500 mL, dose = 0.01 g L^−1^, [O_3_] = 30 mg L^−1^, and flow rate = 60 mL min^−1^	(0.666; 1.895)	Fe (0.32), Ce (0.42)	Ce doping increases ligand missing defects and generates LASs, which react with O_3_ to form O_2_^•−^, ^1^O_2_, and ^•^OH_ads_.	[260]
Ce/Fe–ZIF	Room temperature (24 h)	O_3_	TC (93%, 90 min)	[Pollutant] = 150 mg L^−1^, dose = 1 g L^−1^, pH = 4, O_3_ flow rate = 10 mg min^−1^	(N/A; 0.03)	N/A	O_2_^•−^, ^1^O_2,_ and ^•^OH were generated in the reaction medium.	[261]
Ce/Fe‐NH_2_‐MIL‐88B	Solvothermal (100°C/12 h)	O_3_	LEF (84.2%, 40 min)	[Pollutant] = 10 mg L^−1^, *V* = 100 mL, dose = 0.2 g L^−1^, pH = 7, O_3_ flow rate = 200 mL min^−1^	(N/A; 0.205)	N/A	O_2_^•−^, ^1^O_2,_ and ^•^OH were generated in the reaction medium.	[262]
Ce/Fe‐MIL‐53	Solvothermal (150°C/18 h)	O_3_	ATZ (91%, 5 min)	[Pollutant] = 5 mg L^−1^, V = 1 L, dose = 0.05 g L^−1^, pH = 6.5, O_3_ flow rate = 9.2 mg min^−1^	(2.24; 8.42)	Fe (undetectable), Ce (0.786)	OVs and UMCs facilitated the O_3_ activation, generating ^•^OH, ^1^O_2,_ O_2_^•−^ in addition to surface‐adsorbed oxygen species.	[155]
Fe/Mn‐BTC MOGs	Stirring in ethanol for 24 h (*T* = 25°C, 80°C, and 120°C)	PAA	OFX (81.85%, 1 h)	[Pollutant] = 5 mg L^−1^, *V* = 100 mL, dose = 0.1 g L^−1^, pH = 7, and [PAA] = 1.034 mM	(0.0208; 0.0317)^b^	Fe (undetectable), Mn (2.4)	Both Fe and Mn synergistically activate PAA and generate CH_3_C(= O)OO^•^,^1^O_2_, and ^•^OH.	[159]
Fe/Co‐MIL‐100	Hydrothermal (150°C/12 h)	PAA	SMX (86.4%, 30 min)	[Pollutant] = 5 µM, V = 100 mL, dose = 0.1 g L^−1^, pH = 6, and [PAA] = 50 µM	(N/A; 1.1)	Fe (0.006), Co (0.106)	Activation of PAA generates CH_3_C(= O)OO^•^ and CH_3_C(= O)O^•^	[263]
Fe/M‐BDC (M = Co, Mn, Cu, and Ni)	Solvothermal (160°C/24 h)	PAA	BZF (98.1%, 60 min)	[Pollutant] = 1 mg L^−1^, dose = 0.025 g L^−1^, and [PAA] = 10 mg L^−1^	(0.088; 2.72)	Fe (undetectable) Co (0.159)	Activation of PAA generates various reactive species, including R–O^•^, ^1^O_2,_ and O_2_^•−^, in addition to the high‐valent metal‐oxo species such as Fe(IV) and Co(IV).	[145]
Co/Zn‐ZIF	Solvothermal (120°C/4 h)	PAA	SMX (99.3%, 30 min)	[Pollutant] = 10 µM, *V* = 100 mL, dose = 0.1 g L^−1^, pH = 6, and [PAA] = 0.1 mM	(18.31; 16.1)	Co (0.07) Zn (N/A)	Activation of PAA generates CH_3_C(= O)OO^•^ and ^1^O_2_ as the dominant reactive species, alongside minor contribution from ^•^OH, O_2_^•−^, and high‐valent metal‐oxo species.	[149]

^a^ Ratio of MOF to BMOF.

^b^ Calculated from pseudo‐second‐order (min^−1^ mg^−1^ L).

Abbreviations: AO7, orange II; BZF, bezafibrate; CHXhc, cis‐1,2,3,4,5,6‐cyclohexane‐hexacarboxylic acid; DHTA, 2,5‐dihydroxy‐1,4‐benzenedicarboxylate; Fc, ferrocene; HHTP, 2, 3, 6, 7, 10, 11‐hexahydroxytriphenylene; LEF, levofloxacin; MOGs, metal–organic framework gels; NIM, nimesulide; NOF, norfloxacin; Phen, phenanthrene; SMTR, sulfameter

### 3.1 Photocatalytic Degradation

MOFs are crystalline coordination networks constructed from metal ions or clusters and organic linkers, and can be regarded as extended polymeric complexes. Upon light irradiation, the linker serves as a photon antenna to absorb electromagnetic radiation, while the metal nodes behave as discrete quantum dots. Their photoresponse is determined by the relative energies, spatial distribution, and coupling of metal‐ and linker‐derived orbitals [164]. Depending on the specific metal–linker combination, several electronic transitions may contribute to light absorption and charge generation (Figure 13a): [165, 166, 167, 168] (i) ligand‐to‐metal charge transfer (LMCT), promoting an electron from an occupied ligand bonding orbital to an unoccupied metal d‐orbital (σ or π → d or d*; equivalent to *t*_2g_ or *e*_g_ in octahedral (O*_h_*) coordination geometry) [168], typically occurring in MOFs with high‐valent, electrophilic metal centers bearing low‐lying empty d‐orbitals coordinated by electron‐rich ligands; [167] (ii) metal‐centered (MC) or d–d transitions, involving electron promotion between metal d‐orbitals of different energy within the same metal node (d → d*); (iii) metal‐to‐ligand charge transfer (MLCT), promoting an electron from an occupied metal d‐orbital to an unoccupied ligand antibonding orbital (d or d* → π*), favored for low‐valent metals coordinated by a π*‐acceptor linker; [167], and (iv) ligand‐centered (LC) or intraligand transition, involving electron promotion from an occupied ligand bonding orbital to an unoccupied ligand antibonding orbital within the same linker (σ or π → π*) [168].

**FIGURE 13:**
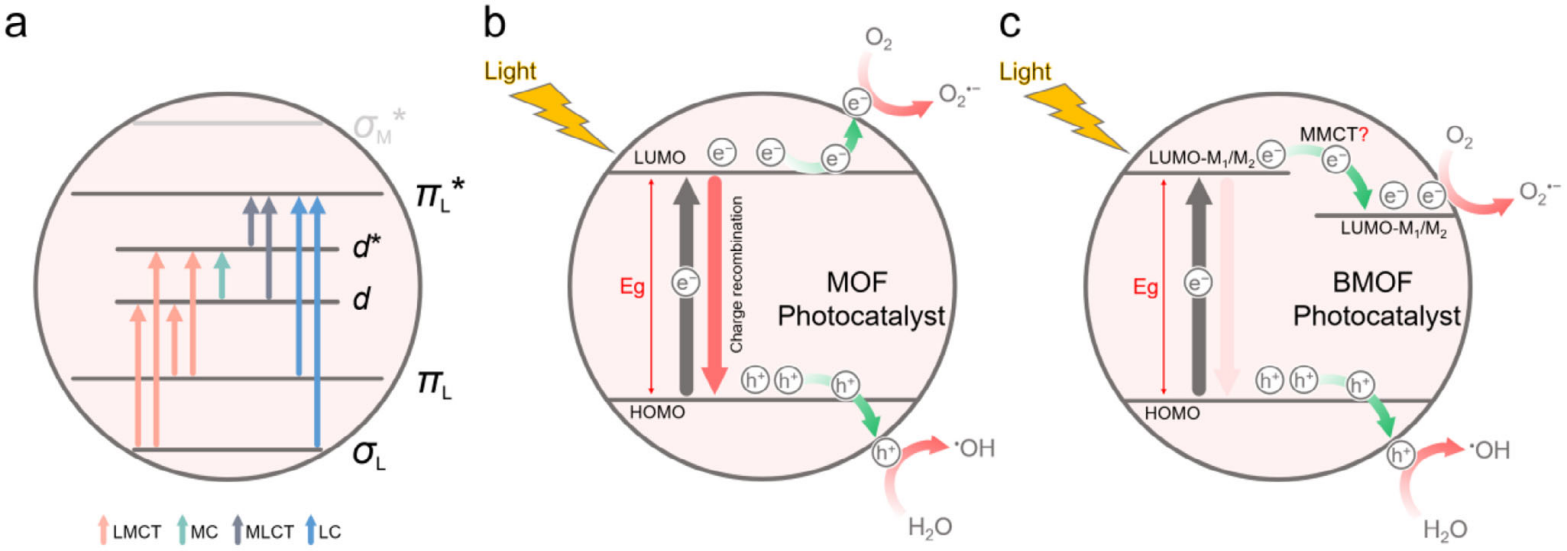
(a) General molecular orbital energy diagram (MOED) for coordination compounds, illustrating the four principal charge‐transfer transitions (LMCT, MC, MLCT, and LC). Schematic illustration of the proposed photocatalytic mechanisms in (b) monometallic MOF and (c) BMOF semiconductors.

These transitions are often described using a simplified semiconductor‐like band model (Figure 13b) [169], in which the valence‐ and conduction‐band edges correspond to occupied and unoccupied states with different metal or ligand contributions. Although useful for discussing charge generation and redox ability, this model can obscure the localized molecular structure of many MOF orbitals. The nature of frontier states therefore should be established before assigning a specific excitation pathway. Upon irradiation, electrons are promoted from the HOMO to the LUMO, generating electrons (e^−^) in the LUMO and holes (h^+^) in the HOMO, which then react with their surroundings to generate reactive oxygen species (ROS) for pollutant degradation.

Many monometallic MOFs show limited visible‐light absorption or rapid excited‐state deactivation, although these properties vary substantially with framework composition [170].

Incorporation of a second metal can modify the orbital energies, electronic coupling, local coordination environment, defect density, and charge‐transport pathways, thereby changing both light absorption and excited‐state dynamics [171]. Upon metal doping, the four transitions above are retained, but their directionality and relative contributions become considerably more complex. First, LMCT has been proposed to occur preferentially at the metal node of higher electronegativity (EN) [45], though this directionality remains experimentally unconfirmed. The second metal may then improve charge separation by capturing photo‐induced electrons from the first metal via metal‐to‐metal charge transfer (MMCT). The combined LMCT–MMCT pathway is termed ligand‐to‐metal–metal charge transfer (LMMCT), in which the electron is promoted from the linker to the first metal and subsequently transferred to the second (Figure 13c) [172]. However, a general molecular orbital energy diagram (MOED) for BMOF systems has yet to be established, leaving the underlying electronic regime unresolved: specifically, (i) the extent of orbital overlap between the two metal nodes [168], with direct implications for interpreting the observed bandgap narrowing and photoluminescence (PL) quenching; and (ii) if discrete levels persist, which metal node is preferentially populated via LMCT and whether it subsequently engages in MMCT.

Photoluminescence quenching upon metal doping is frequently attributed to MMCT‐mediated charge separation; however, this interpretation implicitly assumes the existence of discrete and electronically localized d‐orbitals at each metal center, an assumption that is rarely verified experimentally [173, 174, 175]. Within the localized‐to‐delocalized framework of Robin–Day mixed‐valence theory [173, 176], several electronic regimes may arise depending on the degree of metal–metal orbital mixing and metal–linker coupling (Figure 14). At one extreme, strong orbital overlap between the two metal centers produces delocalized metal‐based molecular orbitals (Figure 14a), making a discrete donor–acceptor MMCT description difficult to apply [173, 176]. In such cases, reduced PL intensity may instead originate from enhanced nonradiative relaxation associated with the resulting lower‐energy delocalized excited states [174, 176]. Conversely, when the metal centers remain electronically localized (Figure 14b), selective LMCT to one metal site, governed by favorable energy alignment and orbital symmetry matching between the ligand and metal orbitals, may enable subsequent MMCT and charge separation [177, 178]. An intermediate regime may also exist in which both metal centers independently participate in LMCT (Figure 14c), with the occurrence of secondary MMCT depending on the energetic alignment and electronic coupling between the two metal sites [175]. Consequently, PL quenching alone cannot be considered definitive evidence of MMCT, and the electronic regime governing a given BMOF must first be established before assigning the origin of the observed emission suppression.

**FIGURE 14:**
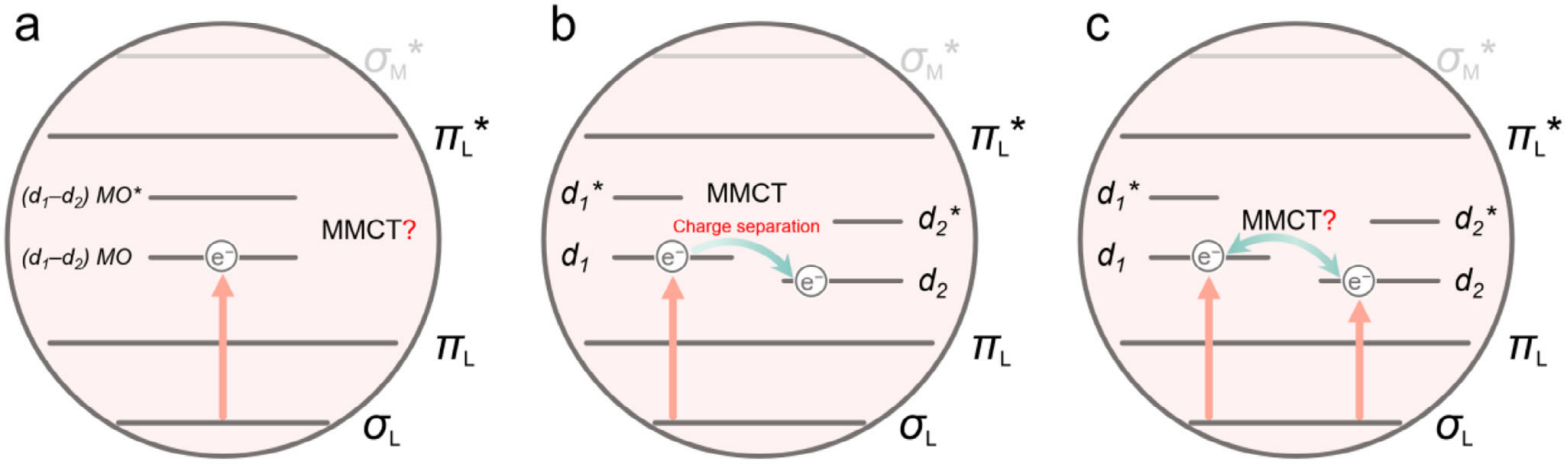
Possible electronic regimes governing MMCT in BMOFs.

Reliable assignment of LMCT, MMCT, or LMMCT requires complementary theoretical and experimental evidence. Orbital‐resolved DFT calculations can identify the metal and ligand contributions to the frontier states and evaluate charge redistribution after excitation [179]. Steady‐state and time‐resolved PL spectroscopy can probe radiative and nonradiative decay [162, 173, 174, 175, 180], while femtosecond transient absorption spectroscopy can resolve short‐lived charge‐transfer intermediates and their kinetics [181]. In situ EPR can detect light‐induced paramagnetic species [182], whereas light‐induced XPS and operando XAS can track changes in oxidation state and local coordination at each metal center [173, 174, 175, 183]. Combining these methods is necessary to distinguish genuine intermetallic charge transfer from alternative excited‐state processes.

Metal doping (Ni, Mn, Co, and Fe) has been shown to substantially alter the electronic transitions of MOF‐5 [164]. In addition to the inherent LLCT of MOF‐5, metal doping induced an LMCT process (Figure 15a), reducing *E*_g_ from 3.94 eV for MOF‐5 to 3.75, 3.68, 3.32, and 2.63 eV for Ni‐, Mn‐, Co‐, and Fe‐doped MOF‐5, respectively. Experimental and theoretical investigations revealed a direct correlation between *E*_g_ and the occupancy of the dopant's d‐orbital, with the fully filled (d10) configuration of Zn(II) (native to MOF‐5) associated with the highest value, whereas Fe(II) (d6), bearing the most accessible empty d‐orbitals, exhibited the lowest *E*_g_ among the dopants, broadly consistent with a higher LMCT probability. These findings highlight the potential of metal doping for the direct, precise tuning of bandgap via modulation of LMCT dynamics.

**FIGURE 15:**
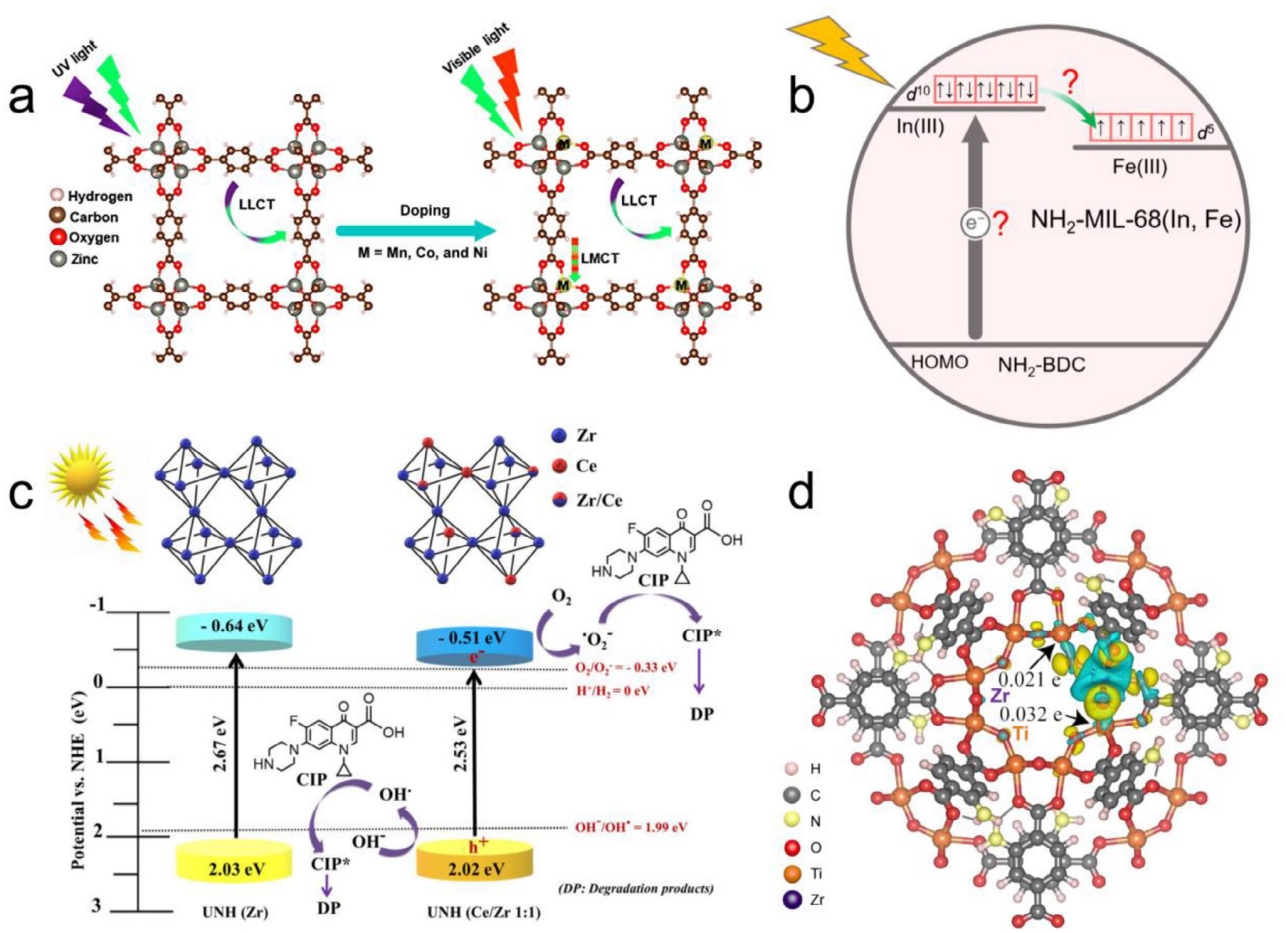
(a) Structure and electronic transitions of M@MOF‐5; Reproduced with permission [164]. Copyright 2022, American Chemical Society. (b) Proposed photocatalytic response of Fe(III)‐doped NH_2_‐MIL‐68(In). (c) Photocatalytic mechanism for the degradation of CIP by UiO‐66(Ce, Zr); Reproduced with permission [184]. Copyright 2022, American Chemical Society. (d) Charge density distribution in Ti/Zr BMOF; Reproduced with permission [45]. Copyright 2023, Elsevier.

Zr doping of NH_2_‐MIL‐125(Ti) increased *k*_obs_ for the photocatalytic degradation of acetaminophen (ACE) from 0.0073 to 0.01214 min^−1^ [146]. This enhancement was attributed to the introduction of new energy levels within the bandgap, which enabled electron trapping from the LUMO, resulting in a substantial decrease in PL intensity. However, the absence of a fully resolved energy level diagram leaves this mechanistic interpretation incompletely substantiated, and future DFT‐based electronic structure calculations would be valuable in confirming the proposed role of Zr in photogenerated electron capture.

Fe(III) incorporation into NH_2_‐MIL‐68(In) narrowed the *E*_g_ from 2.91 to 2.39 eV and substantially altered the framework, reducing particle size, broadening pores, and increasing the S.A., likely owing to the smaller ionic radius of Fe(III) [171]. The *k*_obs_ for photocatalytic degradation of tetracycline hydrochloride (TC‐HCl) and reduction of Cr(VI) were ∼3.4‐ and ∼20‐fold higher, respectively, than those of NH_2_‐MIL‐68(In). Upon illumination, the organic linker absorbed photons and transferred electrons to the In nodes via LMCT, followed by transfer to the Fe centers via MMCT. However, XPS assigned both metals as trivalent centers—In(III) (d10) and Fe(III) (d5)—which leaves the pathway for the proposed LMCT and MMCT transitions open to further clarification (Figure 15b). Holes in the HOMO oxidized TC‐HCl directly, while the photoexcited electrons reduced Cr(VI) to Cr(III) and dissolved O_2_ to O_2_^•−^; the latter drove additional oxidative degradation of the antibiotic.

Charge separation can be further enhanced by grafting an electron‐deficient group onto the BMOF [179]. Introducing 4‐nitro‐2,1,3‐benzothiadiazole (BT) and doping Co into MIL‐53‐NH_2_(Fe) gave Co‐MIL‐53‐NH‐BT with markedly improved photocatalytic properties [180]. Although both BT and Co suppressed charge recombination—with the principal contribution from BT owing to its strong electron‐accepting properties [179]—only Co contributed to bandgap narrowing (*E*g falling from 2.3 to ∼2.0 eV). Consequently, the photocatalytic degradation of ofloxacin (OFX) increased from ∼60% (MIL‐53‐NH_2_) to ∼100% (Co‐MIL‐53‐NH‐BT), with h^+^, ^•^OH, and O_2_^•−^ identified as the active species. However, the mechanism by which Co narrowed the bandgap warrants further investigation.

Ce incorporating into MIL‐53‐NH_2_(Fe) was proposed to generate a low‐energy hybrid orbital through interaction between Ce 4*f* and Fe 3d states, thereby narrowing the bandgap [182]. Combining the two metals reduced the bandgap from 2.54 eV (Ce‐MOF) to 1.18 eV (Ce‐Fe BMOF), with acetaminophen (ACE) degradation rising from 55% to ∼95%. Given the inertness of 4*f* orbitals in chemical bonding, which stems from their highly localized nature and contracted radial distribution [181], direct 3d–4*f* orbital overlap would benefit from computational confirmation of the energy alignment and orbital‐symmetry matching of the two orbitals.

Metal‐catecholates (M‐CATs; M = Co, Ni, Cu) possess both intrinsic antibacterial and photothermal properties [40, 183]. Built from the 2,3,6,7,10,11‐hexahydroxytriphenylene (HHTP) linker, a CoCu‐CAT‐1 BMOF was reported for the synergistic photocatalytic‐photothermal disinfection of *Escherichia coli* [40]. Cobalt incorporation enhanced visible‐light absorption of Cu‐CAT‐1, plausibly because the d^7^ Co(II) centers offer more accessible empty d‐acceptor states for LMCT than the d^9^ Cu(II), analogous to the bandgap–d‐orbital occupancy in MOF‐5 [164]. In addition, the BMOF showed the highest photothermal‐conversion efficiency and the largest temperature rise (Δ*T* ≈ 33°C) among the samples. Consequently, the combined photocatalytic‐photothermal inactivation reached ∼80% compared to ∼40% (thermal) and ∼50% (photocatalytic), attributed to the combined photothermal effect, the intrinsic antibacterial activity of Co and Cu, and photogenerated h^+^ and ^•^OH.

Ce, with its readily accessible Ce(III)/Ce(IV) redox couple, is an effective electron‐trapping center [185]. Doping Ce into the visible‐light‐active UiO‐66‐NH_2_(Zr) slightly narrowed the bandgap (from 2.67 to 2.54 eV) and substantially improved charge separation, as evidenced by PL quenching attributed to electron trapping in the unoccupied 4*f* states of Ce(IV), reportedly yielding long‐lived excited‐state charges [184]. This assumption would benefit from time‐resolved PL measurements. The combined effect of enhanced visible‐light absorption and suppressed charge recombination raised ciprofloxacin (CIP) degradation from ∼40% to ∼91%, with O_2_^•−^ identified as the dominant active species, following the mechanism proposed in (Figure 15c).

Developing multifunctional materials offers opportunities to simultaneously achieve multiple functions or enhance performance through complementary mechanisms. Metal doping provides an effective strategy for designing BMOFs combining the functionalities of their monometallic counterparts. For instance, Al(III)‐MOFs exhibit strong fluoride (F^−^) adsorption capacity [186], whereas Fe(III)‐MOFs show effective photocatalytic degradation performance [187]. Accordingly, an Al‐doped Fe‐BDC was designed to combine F^−^ adsorption and organic pollutant degradation within a single framework [188]. The bandgap narrowed from ∼2.7 eV (Fe‐MOF) to ∼2.0 eV upon Al incorporation, though the origin of this electronic modulation remains unclear, particularly given the wide bandgap of the Al‐BDC counterpart (∼4 eV) [189]. The material achieved ∼97% photodegradation of methylene blue (MB) under solar irradiation, alongside ∼96% F^−^ removal, with fluoride chemisorption following a surface–boundary‐layer mechanism. These results highlight the importance of precise selection of metal combinations to achieve optimal multifunctional performance without compromising the intrinsic activity of either monometallic counterpart.

A chemical, 4,4′,4″,4″′‐(pyrene‐1,3,6,8‐tetrayl) tetra‐benzoic acid (H_4_TBAPy), is an excellent photosensitizer ligand owing to its strong luminescence and electron‐donating properties, arising from the extended π‐electron system, which can efficiently harvest light to metal nodes through LMCT [190]. A Ti/In‐TBAPy BMOF was reported for the adsorption and photocatalytic degradation of bisphenol A (BPA) [48]. The BMOF combined the strong adsorption properties of In‐MOF [191, 192], driven by its large surface area and π–π interactions with phenolic pollutants, alongside the promising photocatalytic activity of Ti‐MOF [193]. Complete BPA removal was achieved through the synergistic effect of adsorption, which shortened the mass‐transfer distance to catalytic active sites, and degradation by ROS generated upon light irradiation. Although all three frameworks achieved 95%–100% BPA removal, each operated through a distinct mechanism—photocatalysis (Ti‐MOF), adsorption (In‐MOF), or synergistic adsorption–photocatalysis (Ti/In‐BMOF)—demonstrating that the removal pathway can be precisely tuned by selecting the metal node composition.

Charge redistribution induced by IVB metals, such as Ti and Zr, can trigger π–π electron‐donor–acceptor (EDA) interactions between the electron‐deficient aromatic rings of the linker and the electron‐rich rings of the pollutant, thereby enhancing adsorption. Accordingly, Zr doping of Ti‐MOF increased the adsorption capacity (*q*_m_) for BPA from ∼311 to ∼3018 µmol g^−1^ [45]. Although the bandgaps remained essentially unchanged after doping, the band structure was rearranged, increasing the degradation efficiency from ∼40% to ∼95%. DFT calculations suggested charge redistribution from Zr to Ti sites (Figure 15d), driven by the higher electronegativity of Ti, resulting in high‐electron‐density reactive centers (HRCs) that promote ROS. The superior removal performance was attributed to the synergy between π–π EDA adsorption and photocatalytic degradation mediated by Ti HRCs.

The photocatalytic performance of FeNi‐BDC BMOF synthesized from an FeNi‐layered double hydroxide (LDH) precursor was compared with that of a material prepared from metal‐nitrate precursors [152]. The LDH‐derived BMOF exhibited superior degradation of organic dyes (89% versus 77% for Congo red), attributed to its higher surface area, improved charge separation, and adsorption capacity; although the relative contribution of each factor was not fully resolved. DFT calculations suggested that Fe and Ni occupy the same SBU and that the dye molecules preferentially adsorb at Ni sites before oxidative degradation by ROS. Although the two materials showed similar XRD patterns, the LDH‐derived sample displayed a uniform two‐dimensional nanosheet morphology, whereas the nitrate‐derived material contained rods and spherical particles associated with separate Fe‐MOF and Ni‐MOF phases. These findings highlight the decisive role of the synthetic strategy in obtaining single‐phase BMOFs and underscore the importance of combining multiple physicochemical techniques to validate phase purity.

In summary, metal incorporation provides a versatile strategy for tuning the electronic structure, charge‐transfer pathways, and functionality of BMOFs for photocatalytic water treatment. Depending on the degree of orbital interaction between the metal centers and the linker, charge transfer may proceed through LMCT, MMCT, or charge redistribution mechanisms, although these processes remain insufficiently understood in many systems. The widespread attribution of PL quenching to MMCT often lacks direct experimental or theoretical evidence, highlighting the need to consider orbital overlap, energy alignment, and electronic localization when interpreting charge‐transfer processes. Beyond electronic modulation, combining metals with complementary properties can introduce additional functionalities, including enhanced adsorption, improved charge separation, and multifunctional pollutant removal. Nevertheless, achieving true synergy requires careful selection of the metal pair, as electronic interactions may either enhance or compromise the intrinsic properties of the individual components. These findings emphasize that the rational design of BMOFs should extend beyond empirical metal doping toward a more fundamental understanding of metal–metal and metal–ligand interactions. Table 1 summarizes the reported BMOFs for photocatalytic wastewater depollution.

### 3.2 Heterogeneous Fenton Catalysis

Fenton catalysis (FC) is widely employed for water depollution [194]. In homogeneous Fenton catalysis, Fe(II) catalyzes the decomposition of hydrogen peroxide (H_2_O_2_; redox potential = +1.78 V versus normal hydrogen electrode, NHE) [195], generating hydroxyl radicals (^•^OH) and Fe(III) (Equation 2) [196]. The ^•^OH radicals (oxidation potential ≈ +1.8–2.7 V versus NHE) [197] are highly reactive species capable of abstracting hydrogen or adding hydroxyl groups, causing oxidative degradation of pollutants (Equations 3 and 4). The produced Fe(III) can be reduced back to Fe(II) by reacting with another H_2_O_2_ molecule (Equation 5) [196].

(2) $$\mathrm{Fe}\left(\mathrm{II}\right)+H_{2}O_{2}\to \mathrm{Fe}\left(\mathrm{III}\right)+HO^{-}{+}^{•}\mathrm{OH}\to \left(k=63-76M^{-1}\;s^{-1}\right)$$

(3)

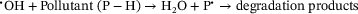



(4)

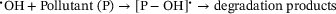

(5) $$\mathrm{Fe}\left(\mathrm{III}\right)+H_{2}O_{2}\to \mathrm{Fe}\left(\mathrm{II}\right)+H{O_{2}}^{•}+H^{+}\to \left(k=0.001-0.01M^{-1}\;s^{-1}\right)$$

Although homogeneous FC exhibits high efficiency in pollutant degradation, it suffers from several drawbacks such as catalyst non‐recovery, strict pH regulation (2.8–3.5), and sludge generation [198, 199], which limit its practical application. These issues can be addressed through heterogeneous Fenton catalysis (HFC), where iron ions (or other transition metal ions, such as Co, Cu, Mn, and Ce) are immobilized, stabilized, or anchored on a solid support [200, 201]. MOFs and BMOFs have shown great potential in HFC [198], owing to their high porosity and crystallinity, satisfactory water stability, ability to chemically bond transition metals within their crystalline structures, and the synergistic effects of different metals. Electron transfer from the catalyst to H_2_O_2_ can occur with or without an external energy source (e.g., light, electricity, and ultrasound) [200].

#### 3.2.1 Ambient HFC

In ambient HFC, electron transfer from BMOFs to H_2_O_2_ occurs without any external energy source. Fe‐MOFs are considered promising candidates for HFC [202]. However, the reaction of Fe(III), the typical metal center in Fe‐MOFs, with H_2_O_2_ is relatively slow [196, 203]. Therefore, increasing the Fe(II) content can improve Fenton catalytic activity by facilitating H_2_O_2_ activation. For instance, doping Fe(II) into MIL‐53(Fe) enhanced the catalytic degradation of 4‐nitrophenol (4‐NP) from ∼68% to ∼95% [43]. Additionally, the density of coordinatively unsaturated metal sites (CUMSs), confirmed to catalyze H_2_O_2_ decomposition [204], significantly increased upon doping, though the origin of these additional CUMSs was not elucidated. Furthermore, the BMOF exhibited good recyclability, with performance decreasing only from 95% to 89% over five consecutive runs, effectively addressing the catalyst recovery limitation of homogeneous FC. Quenching experiments identified surface‐bound hydroxyl radicals (^•^OH_surf_) as the primary reactive species, confirming the heterogeneous catalysis.

The HFC performance of MOFs can be improved by accelerating Fe(III)/Fe(II) redox cycling. Besides its ability to activate H_2_O_2_, copper, with its lower reduction potential (*E*°_Cu(II)/Cu(I)_ = +0.16 V) compared to iron (*E*°_Fe(III)/Fe(II)_ = +0.77 V), is considered a promising candidate for facilitating Fe(III)/Fe(II) cycling, thereby enhancing catalytic activity [205, 208, 209]. Cu(II) doping in Fe(III)‐BDC improved the Fenton degradation of sulfamethoxazole (SMX) from ∼95% (150 min) to complete removal within 120 min, which was attributed to the synergistic activation of H_2_O_2_ by both Fe and Cu and the Cu‐induced acceleration of the Fe(III)/Fe(II) cycle [46].

Another study investigated the catalytic enhancement of MIL‐101(Fe) through combined Cu doping and CUMSs creation [205]. Cu doping induced a partial reduction of Fe(III) to Fe(II), which is expected to enhance Fenton activity. Heat treatment removed water molecules and nonbridging ligands from the framework, generating mesoporosity, surface roughness, and CUMSs (Figure 16a‐I, II), which improved CIP degradation, even in the monometallic Fe‐MOF. In situ DRIFTS indicated a higher density of Lewis‐acidic sites in the BMOF, while time‐dependent in situ Raman spectra showed changes in the H_2_O_2_‐associated signals during reaction, supporting adsorption and activation of H_2_O_2_ at the CUMSs. DFT calculations attributed the improved H_2_O_2_ activation to a decrease in the calculated energy barrier from 0.59 eV for MIL‐101(Fe) to 0.27 eV for CUMSs‐MIL‐101(Fe, Cu), owing to greater accessibility of the unsaturated metal sites and reduced steric restriction around the active centers (Figure 16a‐III, IV). The catalyst also achieved 90%–100% pollutant removal over pH 5–10, extending the operating range beyond that of conventional homogeneous Fenton systems. However, comparison with MIL‐101(Fe, Cu) containing fewer CUMSs would be required to separate the contributions of Cu incorporation from those of the unsaturated coordination sites.

**FIGURE 16:**
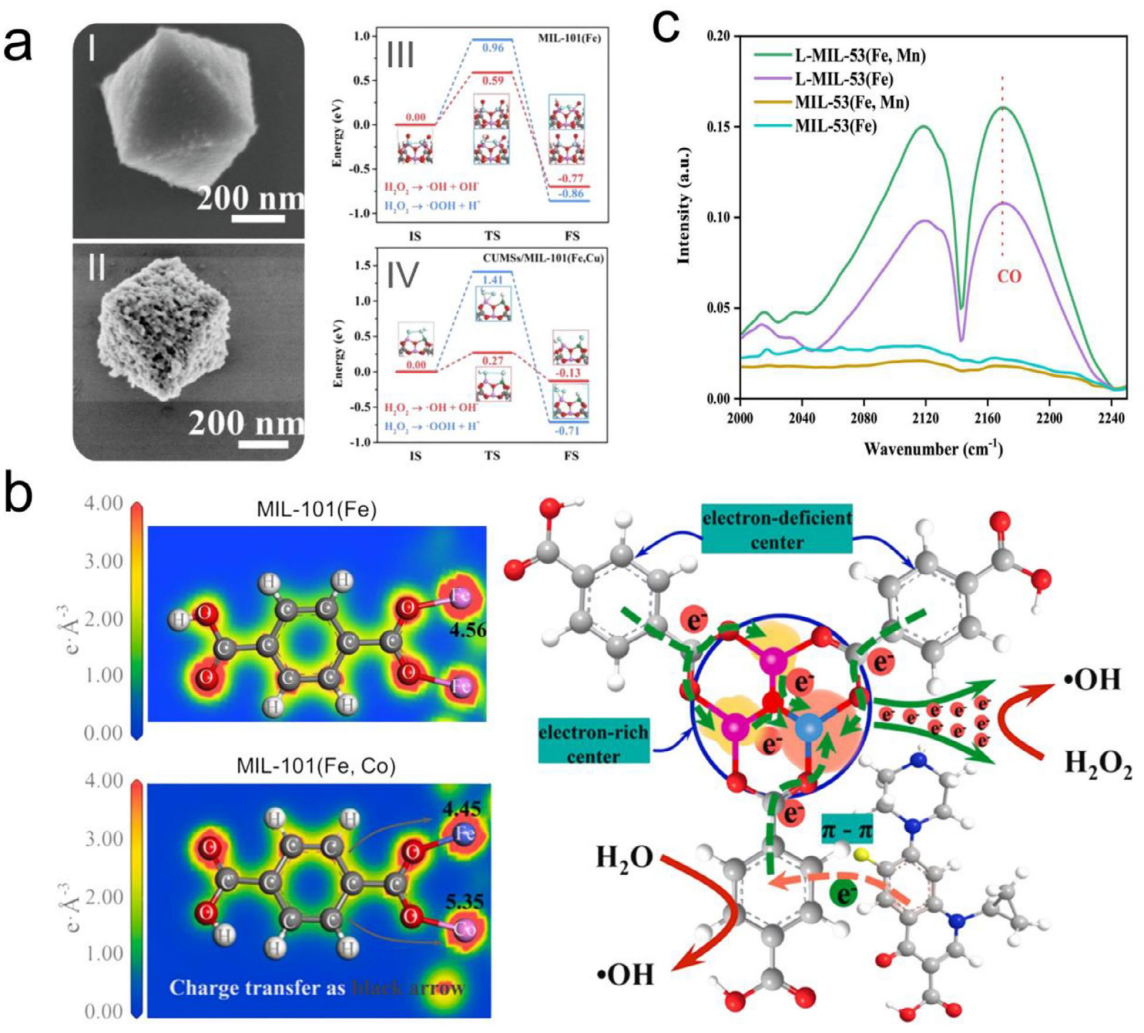
(a) SEM images of (I) MIL‐101(Fe) and (II) CUMSs/MIL‐101(Fe, Cu), and calculated energy profiles for H_2_O_2_ dissociation on (III) MIL‐101(Fe) and (IV) CUMSs/MIL‐101(Fe, Cu) surfaces; Reproduced with permission [205]. Copyright 2021, Elsevier. (b) DFT calculations for the two‐dimensional valence electron density color‐filled maps for MIL‐101(Fe) and MIL‐101(Fe, Co), and the Fenton degradation mechanism of CIP over MIL‐101(Fe, Co); Reproduced with permission [206]. Copyright 2021, Elsevier. (c) In situ CO‐adsorption FTIR spectra of low‐crystalline Mn‐doped MIL‐53(Fe); Reproduced with permission [207]. Copyright 2021, Elsevier.

Fe(II)‐based BMOFs can significantly enhance Fenton catalysis by bypassing the intrinsically slow Fe(III)/H_2_O_2_ reaction. However, Fe(II)–terephthalate coordination bonds are generally expected to exhibit lower stability according to the HSAB principle. BUC‐96(Fe, Cu) was synthesized from Fe(II), Cu(II), and 2,5‐dihydroxyterephthalic acid (H_4_dhtp), employing 4,4′‐bipyridine (bpy) as a pillar linking adjacent 2D sheets [210]. BUC‐96(Fe, Cu) achieved complete degradation of chloroquine phosphate (CQ) within 5 min, compared with 88% removal after 30 min for BUC‐96(Fe). Although bpy was likely introduced to enhance framework stability, its actual role remains unclear, particularly given the measurable iron leaching (∼1 mg L^−1^). Furthermore, the material demonstrated sustained CQ removal in a continuous‐flow reactor, achieving 82.3% removal after 300 min.

Reducing the electron density of Fe(III) centers has been reported to accelerate their activation of H_2_O_2_ [211]. Accordingly, 2‐bromoterephthalic acid (BDC‐Br) was employed to synthesize a bromine‐functionalized FeCu‐BMOF [212]. The degradation of phenol increased from 42% for Fe‐BDC to 76% for Fe‐BDC‐Br and 94% for FeCu‐BDC‐Br, highlighting the promoting effect of both Br incorporation and Cu doping. The high electronegativity and electron‐withdrawing nature of bromine reduced the electron density around the Fe(III) centers, as evidenced by XPS. Further computational evidence is needed to clarify the electronic changes induced by Br incorporation.

As an alternative to Cu, Co incorporation into MIL‐101(Fe) improved CIP degradation from 55% to 98% [206], with in situ DRIFTS and Raman confirming a higher density of CUMSs and sustained H_2_O_2_ activation as in its Cu analogue [205]. However, the structural origin of the CUMSs generated upon Co doping has yet to be established. DFT calculations revealed electron transfer from the linker benzene rings to the transition‐metal sites, driven by the high electronegativity of Co (1.88) and Fe (1.83) (Figure 16b, left). As a result, electron‐deficient linker rings and electron‐rich Fe/Co centers were generated. According to the proposed π–π EDA mechanism, the adsorbed CIP transferred electrons to the electron‐deficient benzene rings, promoting oxidative degradation (Figure 16b, right).

Rapid MW‐assisted synthesis can lead to incomplete coordination of metal species within the framework, thereby generating a high density of CUMSs. FeCo‐BDC synthesized under a short MW reaction time (20 min) exhibited higher OTC removal efficiency, whereas prolonged synthesis reduced catalytic activity, likely due to more complete coordination and a lower density of CUMSs [213]. Similarly, introducing CUMSs into CoFe‐BTC increased amoxicillin (AMX) removal from 46.6% over Co‐MOF to complete degradation over CUMSs‐CoFe‐BTC [214]. However, the absence of a corresponding Fe‐MOF control and a CoFe‐BTC sample without enriched CUMSs makes it difficult to distinguish the separate contributions of Fe incorporation, Co–Fe interactions, and coordinatively unsaturated sites.

Mn‐doped MIL‐101(Fe(II)) was reported for the Fenton degradation of phenol [215]. Driven by the lower electronegativity of Mn (1.55) relative to Fe (1.83), electron density within the SBU accumulated around Fe centers, stabilizing the Fe(II) state, as supported by DFT calculations. Despite the limited activity of Mn‐MOF toward H_2_O_2_ activation, Mn doping improved phenol degradation from 80% to complete removal, suggesting electronic synergy. The catalyst also maintained approximately 85%–100% removal over pH 5–9 and in South Lake and phenol‐containing wastewater, indicating reduced sensitivity to the tested water matrices. Nonetheless, the relatively high iron leaching (4.0 mg L^−1^) suggests limited hydrolytic stability, consistent with the expected weakness of Fe(II)–terephthalate coordination bonds according to the HSAB principle.

#### 3.2.2 Energy‐Assisted Fenton Catalysis

Sometimes, electron transfer from BMOFs to H_2_O_2_ requires external energy input, such as light, electricity, or ultrasound. Low‐crystalline materials have been reported to possess a high density of CUMSs, resulting in enhanced catalytic activity [216, 217]. In this context, low‐crystalline Mn(II)‐ and Cu(II)‐doped MIL‐53(Fe) were synthesized for the photo‐Fenton degradation of CIP [207]. Shortening the solvothermal treatment from 15 to 5 h produced less‐crystalline frameworks with a higher density of CUMSs. Probe‐molecule FTIR using adsorbed CO showed stronger signals associated with Lewis‐acidic unsaturated metal sites in the low‐crystalline materials (Figure 16c). These materials outperformed their more crystalline counterparts, including the corresponding monometallic Fe‐MOF. Although CO‐adsorption FTIR identifies accessible active sites, operando measurements are still needed to determine how these sites evolve during H_2_O_2_ activation. Furthermore, Mn(II) doping reduced *E*_g_ from 2.25 to 1.77 eV, improving photo‐response and photo‐Fenton performance. Notably, although shortened preparation times have been employed with both conventional and MW‐assisted solvothermal syntheses to generate CUMSs [207, 213]. MW heating generally produces crystalline materials, whereas conventional solvothermal synthesis can yield low‐crystalline frameworks.

Designing photo‐Fenton catalysts with high adsorption capacities toward target pollutants can enrich pollutants near the reactive sites, thereby enhancing photo‐Fenton degradation [48]. For example, Ni doping in Fe‐BDC simultaneously improved the adsorption and photo‐Fenton degradation of organic pollutants [35]. The smaller ionic radius of Ni increased the S.A. and mesoporosity [171], resulting in 5.3‐ and 2.6‐fold higher adsorption capacities toward MB and MO, respectively, compared with Fe‐MOF. Consequently, the degradation rates were approximately 1.5–2 times higher than those of the undoped MOF for the same pollutants. Since Ni doping exhibited little influence on the photo‐response of Fe‐MOF, incorporating metals that simultaneously enhance light harvesting and adsorption capacity may provide an effective strategy for designing BMOFs with improved photo‐Fenton performance.

Similar to photocatalytic applications, enhancing light harvesting and narrowing the bandgap can improve photo‐Fenton performance. Vacuum activation of MIL‐100(Fe) partially reduced Fe(III) to Fe(II), generating mixed‐valence CUMSs and decreasing *E*_g_ from 2.73 to 2.41 eV [109]. Consequently, the photo‐Fenton degradation of TC‐HCl improved with increasing activation temperature. The proposed mechanism involved adsorption of H_2_O_2_ (a Lewis base) onto the Lewis acidic CUMSs. Under light irradiation, LMCT reduced Fe(III) to Fe(II), which subsequently transferred electrons to the adsorbed H_2_O_2_, generating ^•^OH. Similarly, Al(III) doping reduced the bandgap of MIL‐88B(Fe) from 2.76 to 2.51 eV, increasing the photo‐Fenton degradation rate constant from 0.012 to 0.028 min^−1^ [218]. However, the origin of the bandgap reduction is yet to be resolved, particularly given the wide bandgap of the Al‐BDC counterpart (∼4 eV) [189].

Three ZnFe‐BMOFs (ZnFe‐BDC, ZnFe‐BTC, and ZnFe‐Fum; Fum = fumarate) were synthesized from industrial galvanizing pickling waste as the Zn and Fe source for the photo‐Fenton degradation of antibiotics [120]. For ZnFe‐BDC, the catalyst/H_2_O_2_ removed 86% of TC‐HCl in the dark, increasing to complete removal under light irradiation, compared with ∼79% removal for Fe‐BDC. The enhanced activity was attributed to improved charge separation induced by Zn doping; however, the origin of this charge separation remains unclear given the closed‐shell d^10^ configuration of Zn(II), which provides limited capability for accepting electrons from Fe centers. Moreover, the addition of excess Zn(II) during synthesis substantially reduced the catalytic performance, highlighting the need for systematic compositional control to obtain frameworks of defined and reproducible compositions. Notably, the pilot‐scale analogue achieved 99% removal under comparable conditions, highlighting practical applicability.

While various energy sources, such as MW [219], ultrasound [220], and electricity [221], have been used in energy‐assisted HFC, only light has been used with BMOFs. Further exploration of other energy sources may provide a good strategy to enhance the catalytic activity of BMOFs.

In summary, MOF‐based heterogeneous Fenton catalysis (HFC) primarily relies on iron‐mediated H_2_O_2_ activation. However, the intrinsically slow reaction of Fe(III), the typical metal center in Fe‐MOFs, with H_2_O_2_, together with the limited availability of Fe(II) species, often restricts the catalytic performance. Consequently, metal doping has emerged as an effective strategy to enhance HFC through several complementary mechanisms (Figure 17). Increasing the concentration of Fe(II) species within the framework, either through Fe(II) incorporation or by introducing metals with lower reduction potentials such as Cu, can promote H_2_O_2_ activation and may facilitate Fe(III)/Fe(II) cycling. Additionally, linkers with electron‐withdrawing moieties can decrease the electron density around Fe(III) centers, thereby enhancing their reactivity toward H_2_O_2_. The generation of coordinatively unsaturated metal sites (CUMSs) through thermal activation, defect engineering, or shortened synthesis times provides additional active sites and lowers the activation barrier for H_2_O_2_ decomposition. Furthermore, metals with substantially lower electronegativities, such as Mn, redistribute electron density within the SBU and stabilize Fe(II) species, while highly electronegative metals such as Co can induce charge redistribution between the linker and metal centers, promoting oxidative degradation pathways. Other strategies include improving pollutant adsorption through metal doping, thereby enriching pollutants near reactive sites and enhancing degradation efficiency. However, these approaches should be balanced against the hydrolytic stability of the framework, as weak metal–ligand coordination bonds, increased defect densities, and highly unsaturated metal centers may promote metal leaching and structural degradation during Fenton operation. For photo‐Fenton degradation using BMOFs, the strategies outlined in Section 3.1 should be considered.

**FIGURE 17:**
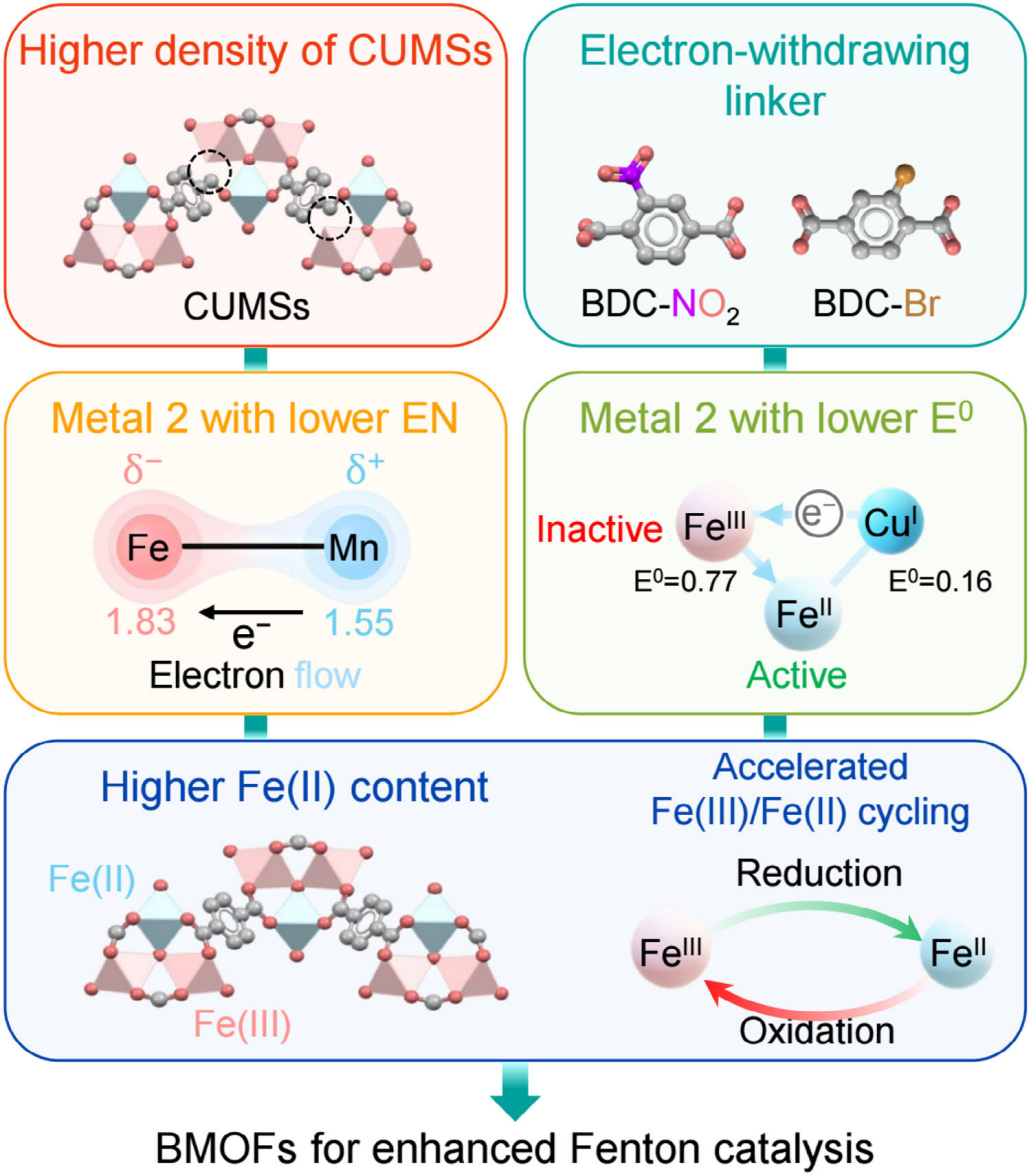
Key design parameters for high‐performance BMOF‐based HFC, including ligand selection, BMOF structure, and doped metal criteria.

Although metals such as Co, Cu, Mn, and Ce have been reported to activate H_2_O_2_ [200, 201], current BMOF research remains largely focused on improving the activation ability of iron‐based MOFs. Expanding the scope of BMOFs for HFC therefore requires exploring non‐iron Fenton‐active MOFs and incorporating low‐toxicity metals with limited amounts of iron to reduce iron consumption while maintaining catalytic performance. Furthermore, most reported BMOFs have been constructed from terephthalate‐based linkers, whereas exploring alternative O‐donor and N‐donor ligands may provide greater opportunities for tuning the electronic structure, improving hydrolytic stability, and enhancing catalytic performance. Table 2 summarizes reported BMOFs for wastewater treatment via heterogeneous Fenton catalysis.

### 3.3 Sulfate Radical Generation (SRG)

Sulfate radical (SO_4_^•−^)‐based AOPs (SR‐AOPs) have been widely applied in wastewater treatment over the last decade [222]. SR‐AOPs involve activating sulfate‐based oxidants, such as peroxymonosulfate (PMS, HSO_5_^−^) or peroxydisulfate (PDS or PS, S_2_O_8_^2−^) (Figure 18a,b), through external energy sources (e.g., UV light, ultrasound, or heat) or catalysts (e.g., transition metal‐based catalysts) to generate highly reactive SO_4_^•−^ that target wastewater pollutants [62].

**FIGURE 18:**
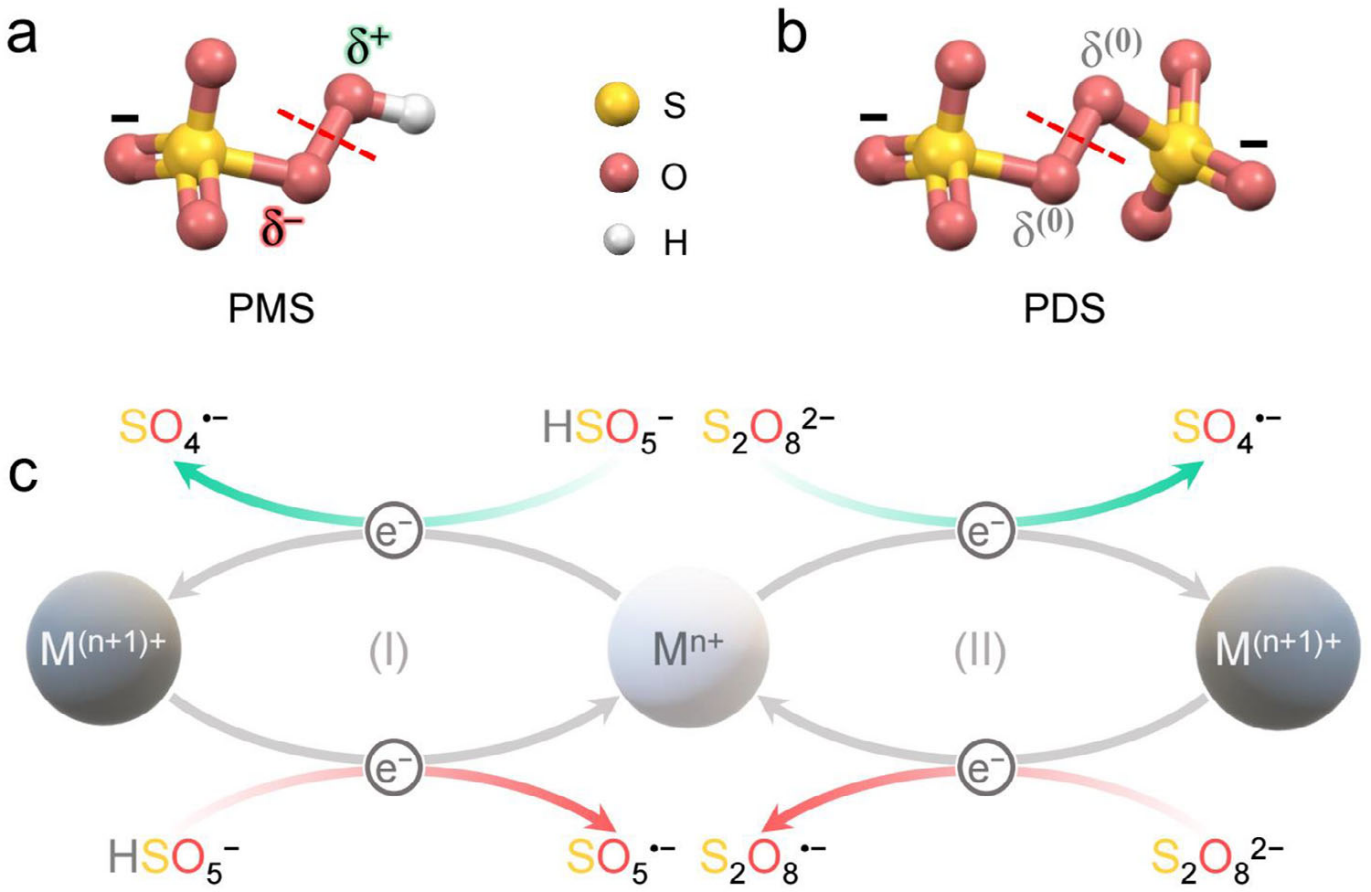
Structures of (a) PMS and (b) PDS, and (c) schematic illustration of the activation of PMS (I) and PDS (II) by transition metal‐based catalysts.

Compared to HFC, SR‐AOPs offer several advantages, including a longer SO_4_^•−^ lifetime (30–40 µs versus 20 ns for ^•^OH), a wider working pH range (2–8), and a higher oxidation potential for SO_4_^•−^ (+2.5–3.1 V, compared to +1.8–2.7 V for ^•^OH) [222]. Additionally, the redox potentials of PMS (+1.81 V vs. NHE) and PDS (+2.08 V vs. NHE) exceed that of H_2_O_2_ (+1.78 V vs. NHE), indicating their superior oxidation power [195]. Furthermore, SO_4_^•−^ reacts more selectively with organic pollutants than ^•^OH, resulting in a higher degree of mineralization [62, 223].

Transition metals, such as Co, Fe, Mn, and Cu, efficiently activate PMS and PDS via an electron transfer mechanism, cleaving O─O bond and generate SO_4_^•−^ (Figure 18c) [222]. Proper metal selection in BMOFs can enhance electron transfer efficiency, boosting activation power [224]. Although PDS has a longer O─O bond and a lower bond dissociation energy (approximately 1.497 Å, 140 kJ mol^−1^, respectively) than PMS (approximately 1.453 Å, 317–377 kJ mol^−1^, respectively) [225, 226], these intrinsic bond descriptors do not directly determine the catalytic activation barrier. The asymmetric structure of PMS produces an uneven electron distribution across its peroxide linkage, giving the terminal peroxide oxygen greater electrophilic character. This polarization promotes selective coordination or interaction with electron‐rich and redox‐active catalyst sites, facilitating interfacial electron transfer and subsequent O─O bond cleavage [226, 227]. In contrast, the two equivalent sulfate groups in PDS produce a more symmetric charge distribution, making its activation more dependent on the adsorption geometry and electronic coupling between PDS and the catalyst. Thus, the easier activation of PMS in many transition‐metal‐catalyzed systems arises primarily from its polarized structure and favorable interfacial reaction kinetics rather than from O─O bond strength alone.

#### 3.3.1 Activation of Peroxymonosulfate (PMS)

##### 3.3.1.1 Co‐Based BMOFs

- Fe/Co‐BMOFs

Given the high PMS activation efficiency of both Co(II) and Fe(II) [228]. FeCo‐BMOFs have garnered significant attention for PMS‐mediated pollutant degradation.

FeCo‐BDC, synthesized from Co(II) and Fe(II), achieved 96.5% phenanthrene (PHEN) degradation within 30 min, with SO_4_^•−^ and ^•^OH identified as the primary reactive species [160]. The weak coordination of Co(II) and Fe(II) with BDC resulted in high Co and Fe leaching (∼3.2–3.8 mg L^−1^)—consistent with the reported dissolution of Co‐BDC under acidic conditions [5], and significant iron release from Fe(II)‐BDC [215]—which points to poor hydrolytic stability of the framework and leaves open the extent of heterogeneous catalysis. This behavior highlights the strong structure–activity relationship in BMOFs and the importance of carefully matching the metal chemistry with the coordination characteristics of the linker (Section 2.1 and 2.5).

Incorporating an electron‐donating moiety, such as an amino (NH_2_) group, into the linker increases the electron density around the SBU, thereby facilitating electron transfer and enhancing PMS activation power [229, 230]. Accordingly, FeCo‐BDC‐NH_2_ was applied to PMS‐mediated degradation of several organic contaminants [47]. Although its Orange G (OG) removal efficiency was similar to that of Co‐BDC‐NH_2_ (98%–100%), Fe incorporation reduced metal leaching from approximately 6.7 mg L^−1^ to 1.3 mg L^−1^, likely because Fe(III)‐carboxylate coordination improved framework stability. Nevertheless, OG degradation continued to near completion after removal of the solid catalyst by hot filtration. This result indicates that dissolved metal species contributed substantially to the observed degradation and that the system cannot be described as exclusively heterogeneous.

Co incorporation into MIL‐101(Fe) decreased the Nyquist semicircle diameter, indicating reduced charge‐transfer resistance, faster interfacial electron transfer, and higher electrical conductivity [38]. Consequently, RhB degradation increased from 38% to 97%, with more than 95% removal across a wide pH range (3–11) and in the presence of Cl^−^ and HCO_3_^−^ ions, while achieving approximately 81% mineralization. Due to the lower reduction potential of iron (*E*°_Fe(III)/Fe(II)_ = +0.77 V vs. NHE) relative to cobalt (*E*°_Co(III)/Co(II)_ = +1.92 V vs. NHE), the Fe‐mediated Co(III)/Co(II) cycling seems thermodynamically favorable.

The catalytic activity of 2D nanosheets reportedly outperforms that of 3D particles [231]. Accordingly, 2D FeCo‐BDC achieved complete RhB degradation within only 5 min. Notably, the BMOF exhibited slightly higher RhB removal, with over 15% higher mineralization, than Co‐BDC, despite containing approximately one‐third less cobalt [232]. The superior RhB degradation was attributed to the selective attack of ^1^O_2_ and O_2_^•−^, as confirmed by EPR, on electron‐donating moieties (─NR_2_). These findings would benefit from theoretical investigations to identify the molecular descriptors governing selective degradation. The BMOF exhibited sustained RhB removal under different reaction conditions and in the presence of several interfering ions.

Fe‐doped Co‐ZIF achieved 97% TC removal within 5 min, compared to 85% for Co‐ZIF [233]. The BMOF maintained high removal efficiencies (∼80%–99%) over a wide pH range (2–12) and in various real water matrices, including tap, river, and medical wastewater. These results indicate broad matrix tolerance under batch conditions. Future studies may also explore the use of Fe(II) precursors, rather than Fe(III), which may potentially favor the formation of more stable Fe‐containing ZIFs.

b. Cu/Co‐BMOFs

In addition to its PMS activation capability and low carcinogenicity [222, 234, 235]. Cu possesses a lower reduction potential (*E*°_Cu(II)/Cu(I)_ = +0.16 V vs. NHE) than Co (*E*°_Co(III)/Co(II)_ = +1.92 V vs. NHE), which may thermodynamically facilitate the reduction of Co(III) to the more active Co(II) species [236]. Moreover, linkers containing terminal hydroxyl groups can increase metal loading of MOFs by coordinating more metal ions, thereby generating additional metal‐based active centers [237, 238]. Based on 2,5‐dihydroxyterephthalic acid (DHTA), CuCo‐MOF‐74 achieved complete MB degradation within 30 min, compared with 74% for the Co‐MOF [239].

A more efficient CuCo‐BMOF was synthesized using hexahydroxytriphenylene (HHTP), an organic linker possessing six hydroxyl groups, for the catalytic degradation of nimesulide (NIM) [240]. Beyond providing abundant active sites through the polyhydroxy ligand, DFT calculations suggested that Co incorporation increased the SBU's electron density, driven by its high EN and strengthened PMS adsorption. Consequently, the BMOF achieved complete NIM degradation within 25 min with approximately 58% TOC removal, outperforming its monometallic counterparts owing to the combined effect of mesoporosity, enhanced PMS adsorption, and bimetallic redox synergy.

Interestingly, although the two frameworks share terminal hydroxyl groups and differ solely in the carboxylate functionality of the MOF‐74 linker, CuCo‐HHTP exhibited substantially lower metal leaching (Co: 0.07 mg L^−1^ and Cu: 0.13 mg L^−1^; pH 5.4) than CuCo‐DHTA (Co: 1.3 mg L^−1^ and Cu: 1.2 mg L^−1^; pH 7) [239], though the differing reaction pH complicates a direct comparison. Further studies under identical conditions are needed to clarify the respective roles of hydroxy and carboxylate groups in governing framework stability.

Furthermore, the degradation of OG dye increased from 94.5% (Co‐BDC‐NH_2_) to 96.4% (CuCo‐BDC‐NH_2_), despite a 50% reduction in cobalt content [236]. Although Cu‐BDC‐NH_2_ exhibited no PMS activation capacity, the improved performance suggests that Cu may facilitate the Co(III) → Co(II) reduction process rather than directly activating PMS.

Recently, we reported the room‐temperature, aqueous synthesis of a 2D CuCo‐BDC BMOF, employing a disodium terephthalate (Na_2_BDC) linker, for the catalytic degradation of naproxen (NPX) [5]. The BMOF achieved complete NPX degradation, compared to less than 5% removal with Cu‐BDC, within 20 min at a PMS concentration as low as 0.25 mM. This superior PMS activation was attributed to the mesoporous channels of the BMOF (Figure 19a), which facilitate the mass transport of both PMS and pollutants [242], together with the Cu(I)‐mediated reduction of the less active Co(III) species to catalytically active Co(II) (Equation 6), as evidenced by Cu L‐edge soft XAS (Figure 19b,c). In situ FTIR further revealed a strong interaction between PMS and the BMOF surface, supporting facilitated PMS activation by the active sites. Cobalt leaching decreased from approximately 60 mg L^−1^ for Co‐BDC, which dissolved completely under the reaction conditions, to 0.16 mg L^−1^ for the BMOF. The catalyst remained active over pH 4‒9 and in the presence of coexisting Cl^−^ and SO_4_^2−^ ions. However, HCO_3_^–^ and CO_3_^2–^ inhibited degradation, showing that resistance to selected ions does not necessarily translate to performance in complex water matrices. Evaluation using real wastewater is therefore needed to determine the practical effect of competing constituents and radical scavengers.

(6) Co(III)+Cu(I)→Co(II)+Cu(II)

**FIGURE 19:**
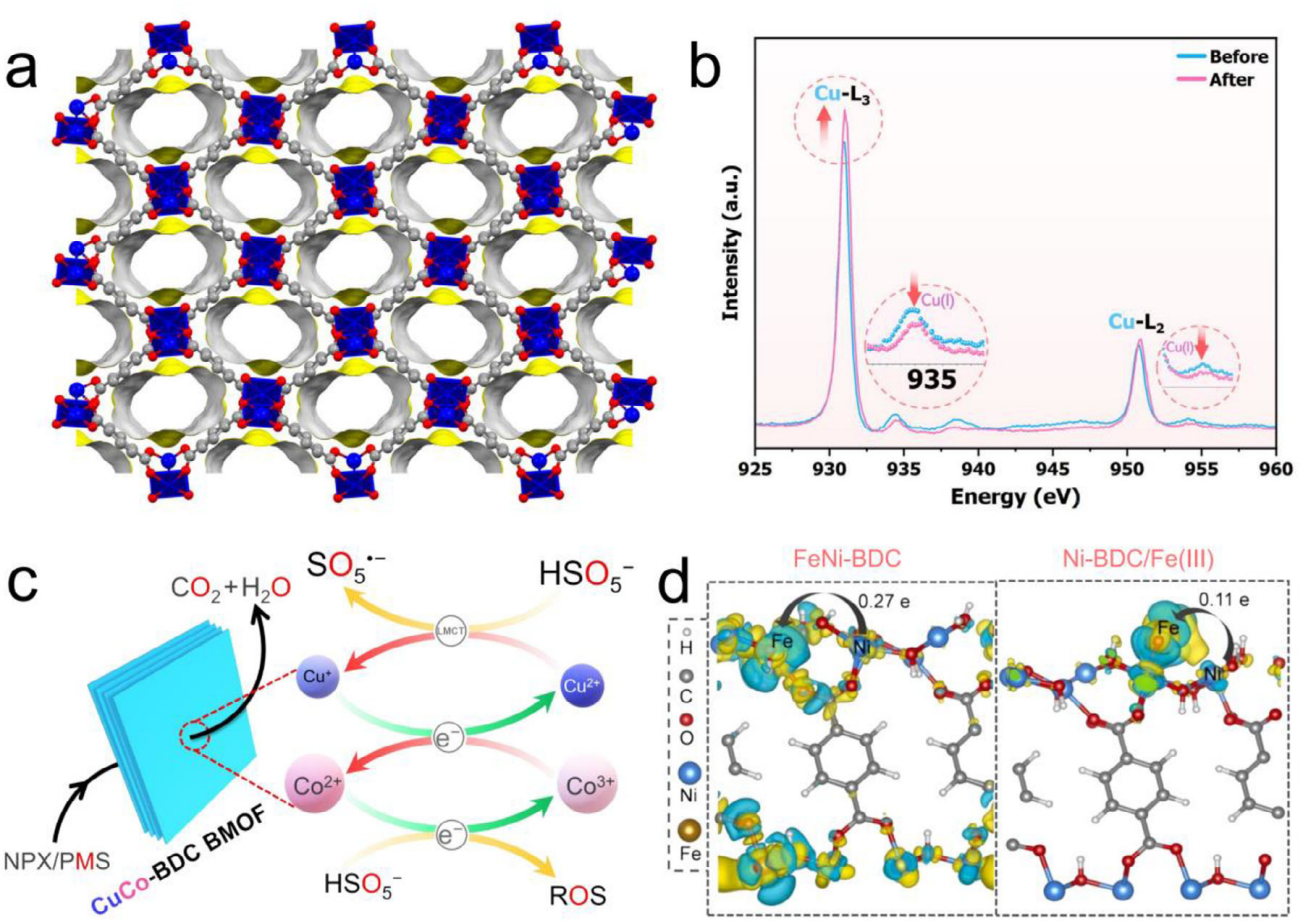
(a) Cross‐sectional view of the empty spaces (voids) within the 2D Cu‐BDC, and (b) sXAS of Cu L‐edges before and after catalytic reactions, and (c) synergistic interaction between Cu(I) and Co(II) during the PMS activation; Reproduced with permission [5]. Copyright 2025, Elsevier. (d) Charge distribution and charge transfer probability in FeNi‐BMOF and Ni‐BDC/Fe(III) physical mixture; Reproduced with permission [241]. Copyright 2023, Elsevier.

All in all, the foreign metal can enhance catalytic activity either by directly activating PMS, increasing reactive species yield, or by accelerating the redox cycles of the primary metal.

c. M/Co‐BMOFs (M = Nb, Mn, Zn, and Al)

Niobium‐based catalysts are known for their low cytotoxicity and high catalytic activity [243]. NbCo‐BDC‐NH_2_ was reported for the catalytic degradation of TC [244]. The BMOF achieved nearly complete degradation within 30 min even after three consecutive cycles and maintained over 90% removal across a wide pH range (3–9) and in various real water matrices (tap, lake, and sea water), highlighting its high stability, efficiency, and potential practical applicability. XPS analysis revealed simultaneous Co(II) → Co(III) and Nb(IV) → Nb(V) transformations during the reaction, suggesting the participation of Nb(IV) either through direct PMS activation or by facilitating the regeneration of Co(II), as proposed in (Equations 7 and 8). However, thermodynamic data on the Nb(V)/Nb(IV) redox couple are currently lacking; further experimental and theoretical validation would clarify the role of Nb in reducing Co(III).

(7) $$\text{Nb}\left(\text{IV}\right)+{\text{HSO}}_{5}^{-}\to \text{Nb}\left(V\right)+{\text{SO}}_{4}^{•-}+{\text{OH}}^{-}$$

(8) Nb(IV)+Co(III)→Co(II)+Nb(V)

Given the PMS activation capability of Mn(II) [245], a MnCo‐BMOF, denoted BUC‐101, was synthesized using *cis*‐1,2,3,4,5,6‐cyclohexane‐hexacarboxylic acid (CHXhc) as the linker [246]. The BMOF achieved complete RhB degradation within 25 min at a PMS concentration as low as 0.2 mM. The enhanced activity was attributed to the simultaneous activation of PMS by both Co(II) and Mn(II), while the relatively low reduction potential of Mn (*E* ^0^_Mn(III)/Mn(II)_ = +1.51 V vs. NHE) [246] may additionally facilitate the Co(III) reduction.

Other catalysts, such as Co‐ZIF‐8(Zn) [97] and Co‐MIL‐53(Al) [153], have also shown promise for PMS‐mediated pollutant degradation; however, the specific roles of the secondary metals and the underlying synergistic mechanisms remain largely unexplored.

##### 3.3.1.2 Fe‐Based BMOFs

Several FeCu‐BMOFs have been reported for efficient PMS‐mediated pollutant degradation [247, 248, 249], generally exhibiting superior performance compared with their monometallic counterparts. This enhancement has been attributed to the complementary roles of Fe and Cu, which can both activate PMS and possess favorable reduction potentials that may facilitate their redox cycling [222, 250]. However, the respective roles of Fe and Cu in oxidant activation and their proposed redox synergy remain insufficiently understood, warranting systematic mechanistic investigations.

#### 3.3.2 Activation of Peroxydisulfate (PDS or PS)

##### 3.3.2.1 Ambient‐PDS Activation

Fe‐MOFs have been employed for PDS‐mediated pollutant degradation [251, 252], but their reactivity is insufficient [253]. The catalytic degradation of PHEN using the Co‐doped Fe(II)‐MOF‐74/PDS system increased from approximately 15% with Co‐MOF and 17% with Fe‐MOF to 35% with FeCo‐BMOF [238]. Despite the relatively low efficiency, the BMOF outperformed its monometallic counterparts, suggesting either a cooperative (redox) or an additive synergy. In contrast, the BMOF/PMS system achieved complete PHEN removal within 30 min. However, the considerable Fe (∼3.0 mg L^−1^) and Co (∼2.1 mg L^−1^) leaching and the pronounced decline in activity to ∼60% after the fourth cycle indicate the limited hydrolytic stability of the framework, which may be associated with the relatively weak coordination of carboxylate linkers to both Fe(II) and Co(II), similar to that reported for CuCo‐MOF‐74 [239].

Cu‐doped MIL‐53(Fe) was synthesized via a MW‐assisted route, denoted MW‐53(Fe, Cu), for PDS‐mediated degradation of norfloxacin (NOF) [254]. Notably, the MW‐synthesized materials exhibited higher activity than their solvothermal counterparts, even for the undoped Fe‐MOF, while the *k_obs_* of MW‐53(Fe, Cu) was nearly 12 times that of the undoped MOF. This improvement was mainly associated with the larger population of well‐defined crystallites and the additional mesoporosity generated during microwave‐assisted synthesis. The resulting pore structure improves access to the metal sites and facilitates the transport of both PDS and NOF through the framework [5, 242], whereas the solvothermal analogue remained predominantly microporous. Cu incorporation may further modify the local redox environment and promote Fe(III)/Fe(II) cycling, although the specific electronic effect of microwave irradiation itself has not been directly established. These results demonstrate that the synthesis method can strongly influence catalyst morphology, pore accessibility, and redox performance. The material retained approximately 84% NOF removal after five cycles and maintained 93%–97% degradation in three real water matrices. These results indicate short‐term reusability and matrix tolerance, although five batch cycles remain insufficient to establish long‐term operational stability.

A ferrocene‐based CoNi‐BMOF containing 1,1′‐ferrocenedicarboxylic acid (Fc) as the linker was reported for PDS‐mediated degradation of emerging contaminants [161]. Because the Fe center is located within the Fc linker rather than serving as a framework node, the material is more accurately described as a CoNi‐BMOF. The CoNi‐Fc BMOF achieved almost complete BPA removal within 5 min, compared with approximately 80% removal for its monometallic counterparts. Notably, the material exhibited sustained removal efficiencies (80%−100%) across various coexisting species (e.g., Cl^–^, H_2_PO_4_^–^, HCO_3_^–^, and HA), a broad pH range (3−9), and real water matrices (tap and lake water sources). The seven‐line (1:2:1:2:1:2:1) DMPOX signal (5,5‐dimethyl‐1‐pyrrolidone‐*N*‐oxyl) suggests the over‐oxidation of the DMPO by reactive species in a strongly oxidative environment, indicative of an intense oxidative flux [255]. However, the assignment of DMPOX formation exclusively to ^1^O_2_ may require further verification, particularly because N_2_ bubbling exerted a negligible effect on degradation, which appears less consistent with a mechanism dominated by O_2_‐derived species such as ^1^O_2_ and O_2_^•−^.

Furthermore, incorporating both Co and amino groups into Fe‐MOF enhanced TC degradation from 77.6% (Fe‐MOF) to 85.3% (Fe/N‐MOF) and 98.6% (FeCo/N‐MOF) [39]. Beyond the synergistic role of Co, the amino group improved PDS activation by increasing the electron density over the SBU. Moreover, nitrogen‐rich regions have been reported to promote ^1^O_2_ generation [230], potentially contributing to the enhanced reactive species production and catalytic performance.

##### 3.3.2.2 Energy‐Assisted PDS Activation

Due to the lower reactivity of PDS compared to PMS, as discussed previously, external energy sources can facilitate electron transfer from BMOFs to PDS, thereby enhancing its activation. MIL‐101(Fe)/PDS was reported for the degradation of organophosphorus esters under light irradiation [256]. Despite achieving complete degradation within 180 min, the study employed a relatively low pollutant concentration. However, Cu doping into MIL‐101(Fe) improved TC degradation in the PDS/vis system from 52% to ∼91%, accompanied by a fourfold increase in *k*_obs_ [41]. Cu doping doubled the Fe(II) proportion, the primary PDS activator, and reduced the PDS adsorption energy according to DFT calculations (*E*_ads_ = −5.92 versus −3.46 eV), indicating a stronger PDS adsorption. Upon light irradiation and charge separation by Cu centers, as evidenced by the PL quenching, PDS trapped photoinduced electrons, leading to its activation and generation of SO_4_^•−^ and ^•^OH. Therefore, both Cu and PDS acted as mediators for the photo‐induced electrons. However, the origin of Fe(II) doubling, along with the mechanism of Cu‐induced charge separation, remains to be clarified.

Embedding iron into appropriately designed MOFs can result in robust BMOFs with precise electron transfer and reduced toxicity. Fe(III) incorporation into Ni‐BDC yielded FeNi‐BDC, which achieved complete TC degradation under visible‐light‐assisted PDS activation, surpassing both Ni‐MOF (15%) and Fe‐MOF (31%) controls, suggesting a cooperative synergy [241]. The homogeneous Fe(III)/PDS and the physically mixed Ni‐BDC/Fe(III)/PDS systems achieved ∼20% and 88% removal, respectively. This behavior was attributed to the enhanced charge transfer within FeNi‐BDC (0.27 e^–^) compared with Ni‐BDC/Fe(III) (0.11 e^–^), as revealed by DFT calculations (Figure 19d), underscoring the importance of local coordination environment in facilitating efficient and precise electron transfer between heterometallic pairs. In situ XPS under light irradiation revealed new Fe(II) and Ni(III) signals, indicative of the simultaneous participation of both metal centers in the catalytic process. However, given the higher estimated redox potential of Ni (E^0^_Ni(III)/Ni(II)_ = +2.3 V vs. NHE) [257], relative to Fe, the thermodynamic feasibility of the proposed Ni‐induced Fe(III)/Fe(II) cycling merits further analysis. Standard aqueous redox potentials cannot be directly transferred to framework‐bound metal centers because coordination geometry, ligand field, pH, and local electronic structure can substantially alter their effective redox behavior. Operando oxidation‐state measurements would therefore be valuable for confirming the direction of electron transfer.

UiO‐66 is a highly stable MOF with a relatively wide bandgap of approximately 3.82 eV [30]. Fe doping into UiO‐66(Zr) reduced *E*_g_ to approximately 2.91 eV and improved charge transfer efficiency, exhibiting ultracapacitor‐like behavior, which significantly improved the photocatalytic PDS‐mediated sulfameter degradation from ∼35% to ∼90% [258]. The BMOF maintained approximately 90% degradation efficiency after five consecutive cycles, indicating good stability. Analogously, Fe(III) doping of NH_2_‐MIL‐125(Ti) reduced its *E*_g_ from 2.69 to 1.74 eV [259], achieving complete OG degradation within 10 min, compared to only 10% with NH_2_‐MIL‐125(Ti). The degradation mechanism involves PDS activation by capturing the photoinduced electrons, generating SO_4_^•−^ or O_2_^•−^, in addition to direct photocatalytic degradation.

#### 3.3.3 Various Mechanisms of SRG by BMOFs

Based on reported studies of PMS and PDS activation by BMOFs, four mechanisms have been proposed to elucidate the interaction between the two metals and the sulfate‐based oxidant, using PMS as a model system. These mechanisms, outlined in Figure 20, were postulated based on the role of the doped metal (M_2_) in enhancing the SRG, considering the primary metal (M_1_) as a highly efficient PMS activator, such as Co. The reaction mechanism depends on two factors: a) the activation capacity of M_2_—within the framework—for the oxidant, and b) its relative reduction potential compared to the primary metal.

- In the type I mechanism, only the primary metal (M1) activates PMS, generating sulfate radicals and undergoing oxidation. The oxidized M1 species is subsequently reduced by accepting electrons from PMS. This mechanism predominantly occurs in inert MOFs doped with a highly active PMS activator, such as Co. The secondary metal (M2) plays a negligible role in PMS activation and cannot facilitate the reduction of oxidized M1, likely due to its incompatible reduction potential with that of M1, leading to minimal catalytic improvement. However, doping with the primary metal can mitigate its leaching.
- In the type II mechanism, both metals independently activate PMS. The reduction potential of M2, however, is insufficient to reduce oxidized M1. Consequently, the observed enhancement primarily arises from the synergistic activation of PMS by both metals.
- In the type III mechanism, only the primary metal (M1) activates PMS, similar to type I. However, the low reduction potential of M2 aids in reducing oxidized M1, in conjunction with PMS. This acceleration of M1's reduction step enhances catalytic performance.
- In the type IV mechanism, both metals can independently activate PMS. Also, the low reduction potential of M2 facilitates the reduction of oxidized M1. Synergistic PMS activation and electron transfer between the two metals enhance overall catalytic activity.

**FIGURE 20:**
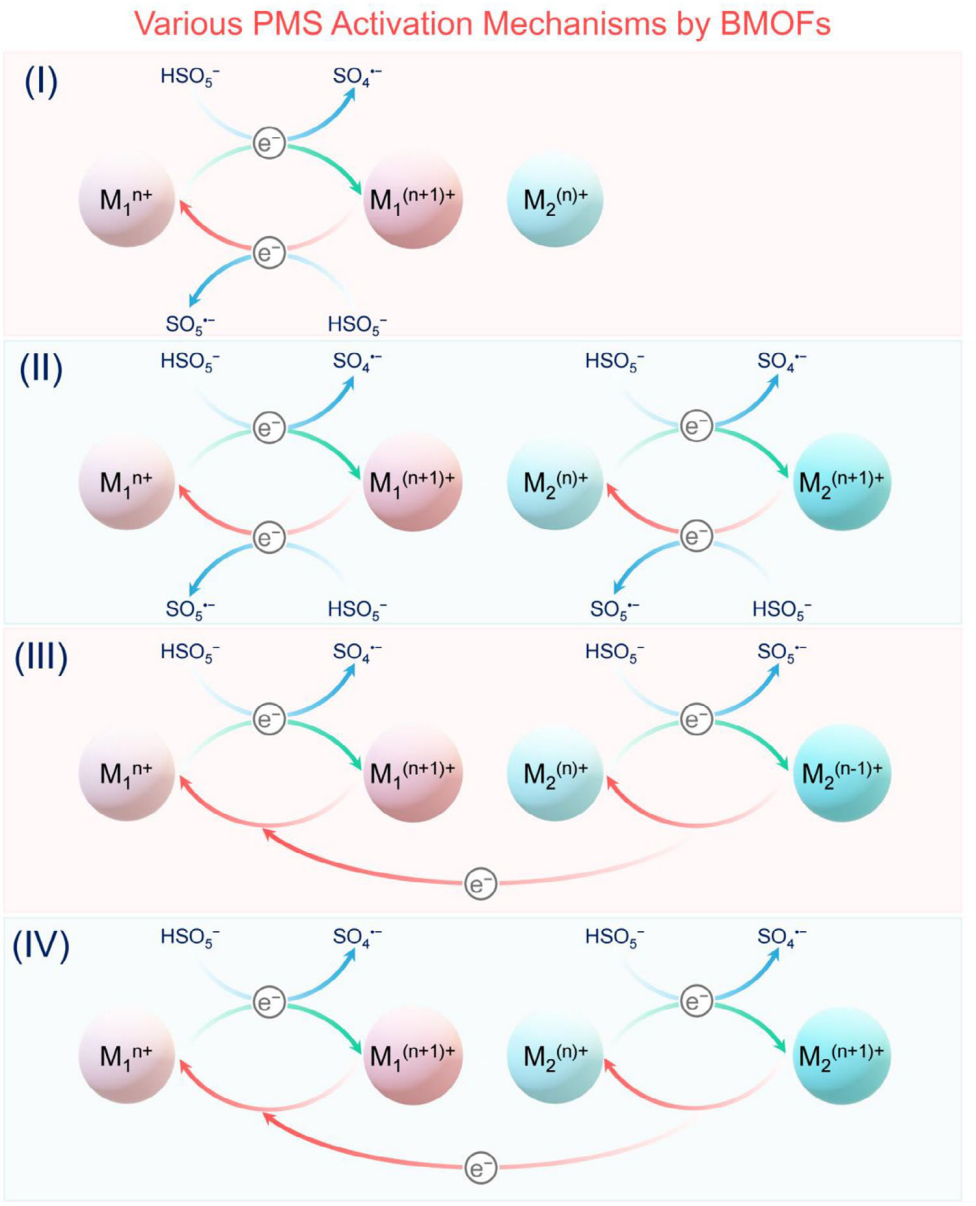
Various mechanisms for PMS activation by BMOFs. These four synergistic mechanisms are generally applicable to other oxidant‐based AOPs.

In conclusion, BMOFs have demonstrated potential for SR‐AOPs, sustaining pollutants under various reaction conditions, encompassing a wide pH range, in the presence of coexisting species, and in real water matrices. The incorporation of a second metal can enhance catalytic performance through several complementary mechanisms, including direct PMS/PDS activation, acceleration of the redox cycling of the primary metal, modulation of the electronic structure of the SBU, and improved oxidant adsorption. Under photo‐assisted conditions, similar to traditional photocatalysis, heterometallic incorporations may additionally reduce the bandgap and promote charge separation, facilitating simultaneous oxidant activation and photocatalysis. Translating these advantages into effective catalysts depends on several design considerations:

- Incorporating cobalt, the best PMS activator, and Fe, the best PDS activator, into MOFs can enhance their activation capacity and catalytic activity. However, the second metal should possess:
- A good activation capacity.
- A lower reduction potential than the primary metal to accelerate its reductive regeneration.
- Employing hydroxy‐terminated ligands can enhance catalytic activity by increasing metal loading within the BMOF.
- Incorporating electron‐donating groups into the organic linker can enhance electron density within the metal cluster, thereby promoting oxidant activation.
- Utilizing stable frameworks (such as ZIFs incorporating low‐valent metals and MILs featuring high‐valent metals) can enhance catalytic performance across a wide pH range while minimizing metal leaching, thereby improving catalyst recyclability.
- 2D BMOFs have exhibited superior catalytic activity compared to other structural configurations in SR‐AOPs.

Despite encouraging activities, several recurring issues temper the reported performance and point to clear priorities for future work:

- Hydrolytic instability and leaching remain the primary challenge. Many systems release several mg L^−1^ of metal and lose activity upon cycling, while hot‐filtration frequently reveals underreported homogeneous contributions. The development of highly stable BMOFs and standardized stability reporting, including leaching quantification and hot filtration, is essential to reduce leaching while distinguishing genuine heterogeneous catalysis from homogeneous artifacts.
- Synergistic effects are frequently inferred from broad and overlapping XPS signals, with misassignment cases, necessitating advanced operando techniques and theoretical calculations to validate the proposed redox interactions.
- The lack of appropriate monometallic controls frequently obscures the actual contribution of the secondary metal, emphasizing the need for more rigorous experimental design.
- The widespread inhibition by HCO^3−^ and CO3^2−^ highlights the importance of systematic evaluation in real water matrices.

Despite being recognized as PMS activators, some metals, such as Cu, appear to lose their intrinsic PMS/PDS activation capability upon incorporation into MOFs [236]. This behavior highlights the critical role of the local coordination environment and warrants further investigation to understand the factors governing oxidant activation in MOFs. Table 3 summarizes the reported BMOFs for wastewater treatment through sulfate radical generation.

### 3.4 Catalytic Ozonation

Catalytic ozonation (CA‐O_3_) is an effective AOP for wastewater decontamination [264, 265]. CA‐O_3_ catalytically decomposes ozone (O_3_) into ROS such as ^•^OH, O_2_^•−^, and ^1^O_2_, employing catalysts like metal oxides, metal‐free catalysts, and immobilized noble metal catalysts [266, 267]. CA‐O_3_ outperforms other AOPs with its ease of handling, eco‐friendliness, and the broad‐spectrum disinfection capability of O_3_ [264, 265]. In addition, the high redox potential of O_3_ (+2.07 V vs. NHE), exceeding that of H_2_O_2_ (+1.78 V vs. NHE) and PMS (+1.81 V vs. NHE) and comparable to PDS (+2.08 V vs. NHE), reflects its electron‐accepting capability [195, 268]. Moreover, CA‐O_3_ can achieve a high degree of mineralization [266, 269]. Various metals, such as Fe, Co, Ni, Zn, Mn, Ce, Ag, and Cr, have demonstrated superior performance in ozone activation [270].

CoNi‐PMA (PMA: pyromellitic acid; 1,2,4,5‐benzenetetracarboxylic acid) was the first BMOF reported for CA‐O_3_ [72]. The atrazine (ATZ) degradation efficiency improved from 67% with Co‐MOF to 94% with CoNi‐BMOF within 20 min, accompanied by a near‐doubling of the mineralization efficiency, via the ROS ^•^OH, O_2_^•−^, and ^1^O_2_. Additionally, H_2_O_2_ was generated during the catalytic process, according to Equation (9), with CoNi‐BMOF achieving the highest yield compared to its monometallic counterparts (Figure 21a). H_2_O_2_ was proposed to contribute to additional ROS generation through direct reactions with Co(II), Ni(II), and O_3_, while also accelerating the redox cycles of Co(II)/Co(III) and Ni(II)/Ni(III), following Equations (10)–(16). Additional control experiments on ATZ degradation by catalysts/H_2_O_2_ would help to isolate the H_2_O_2_ contribution to the overall removal. The presence of a DMPOX signal in the EPR measurements may indicate the strong oxidizing capability of the system [255]. Notably, the BMOF retained ∼93% removal over four consecutive cycles, confirming good reusability; however, the leaching of both metals (∼1.2–1.5 mg L^−1^) points to limited robustness of the metal–ligand coordination.

(9) $$2O_{2}^{•-}+2H^{+}\to H_{2}O_{2}+O_{2}$$

(10) $$H_{2}O_{2}+O_{3}\to 2{\text{OH}}^{•}+3O_{2}$$

(11) $$\mathrm{Co}\left(\mathrm{II}\right)+H_{2}O_{2}\to \mathrm{Co}\left(\mathrm{III}\right)+OH^{•}+OH^{-}$$

(12) $$\mathrm{Ni}\left(\mathrm{II}\right)+H_{2}O_{2}\to \mathrm{Ni}\left(\mathrm{III}\right)+OH^{•}+OH^{-}$$

(13) $$\text{Co}\left(\text{III}\right)+H_{2}O_{2}\to \text{Co}\left(\text{II}\right)+{\text{HO}}_{2}^{•}+H^{+}$$

(14) $$\text{Co}\left(\text{III}\right)+{\text{HO}}_{2}^{•}\to \text{Co}\left(\text{II}\right)+O_{2}+H^{+}$$

(15) $$\text{Ni}\left(\text{III}\right)+H_{2}O_{2}\to \text{Ni}\left(\text{II}\right)+{\text{HO}}_{2}^{•}+H^{+}$$

(16) $$\text{Ni}\left(\text{III}\right)+{\text{HO}}_{2}^{•}\to \text{Ni}\left(\text{II}\right)+O_{2}+H^{+}$$

**FIGURE 21:**
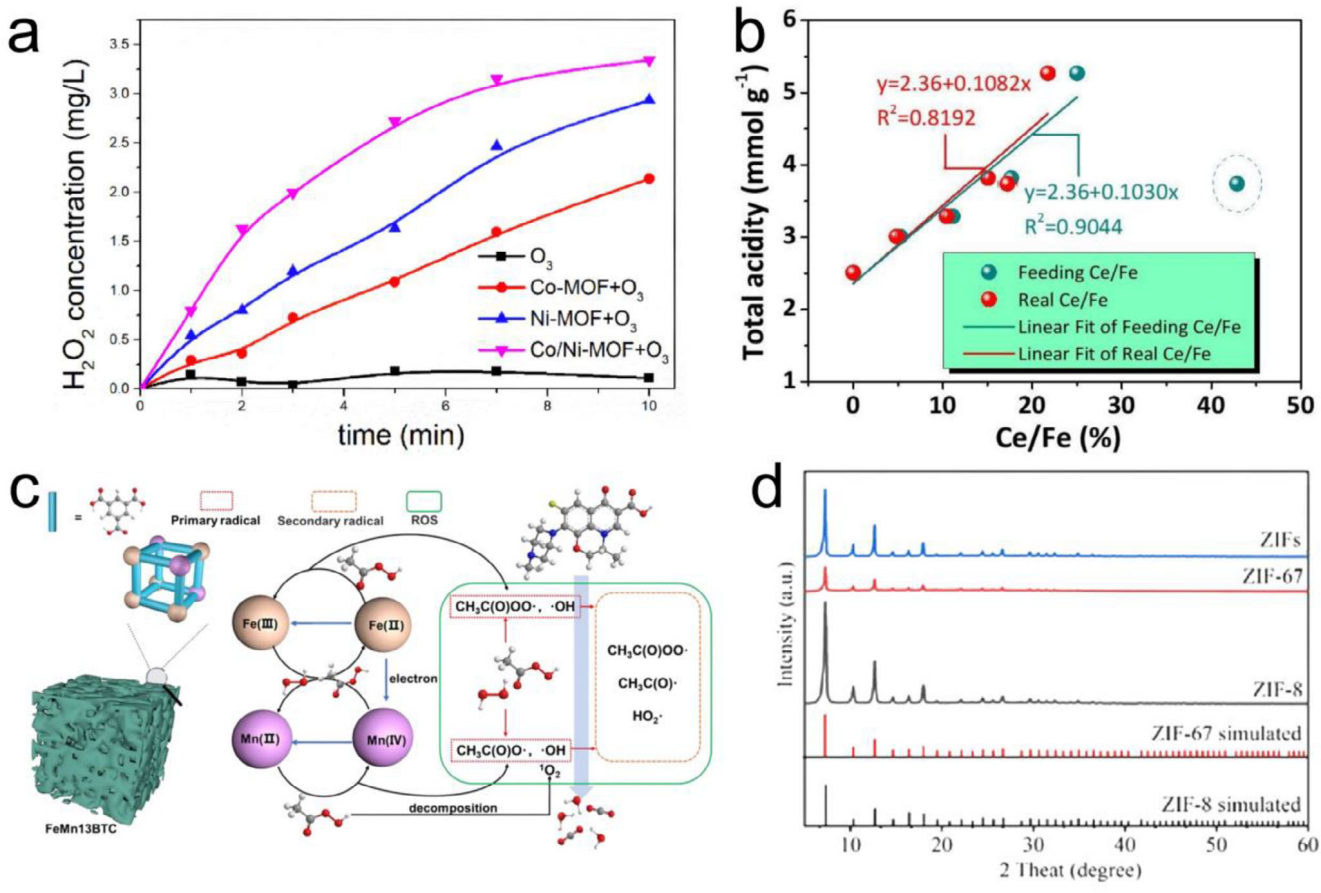
(a) Concentration of H_2_O_2_ formed in different MOF and BMOF/O_3_ systems; Reproduced with permission [72]. Copyright 2020, Elsevier. (b) Effect of Ce doping on the total acidity of MIL‐88A(Fe); Reproduced with permission [260]. Copyright 2021, Elsevier. (c) Presumed mechanism of the synergistic PAA activation by both Fe and Mn species; Reproduced with permission [159]. Copyright 2023, American Chemical Society. (d) XRD patterns of ZIF‐8, ZIF‐67, and CoZn‐ZIF; Reproduced with permission [149]. Copyright 2025, Elsevier.

Iron‐based MOFs have demonstrated potential in CA‐O_3_ for pollutant removal [271]. Similar to HFC, increasing CUMSs by creating ligand‐missing defects can enhance CA‐O_3_ performance. As discussed previously, metal doping can increase the density of CUMSs without requiring heat treatment [43]. Ce doping of MIL‐88A(Fe) created ligand‐missing defects and increased the Lewis acidity (Figure 21b) [260], likely arising from the difference in the coordination numbers of metals [272, 273]. Notably, Ce doping progressively shifted the framework toward a more mesoporous character, which may facilitate the mass transport of pollutants and oxidants to the active sites [5, 242]. Consequently, salicylic acid (SA) degradation increased from 88% (MIL‐88A(Fe)) to complete removal (MIL‐88A(Fe, Ce)), maintaining this performance across a wide pH range (∼3.2–9.6). Adding phosphate, a strong Lewis‐acid blocker, suppressed the catalytic enhancement almost entirely—degradation matched ozonation—confirming that surface Lewis acid sites are the predominant active sites. Notably, the low metal leaching (∼0.35–0.45 mg L^−1^), relative to the Co/Ni‐PMA system [72], may reflect the robust coordination of Ce/Fe with the carboxylate linker.

CeFe‐2‐MeIm was synthesized via simple coprecipitation at room temperature and reported for O_3_‐mediated TC degradation [261]. Although the XRD pattern of CeFe‐2‐MeIm closely resembled that of monometallic Ce‐2‐MeIm, the crystallographic structure of the parent Ce‐2‐MeIm remains unreported, leaving the structural identity of the proposed BMOF unconfirmed. Moreover, the chemical oxygen demand (COD) removal efficiencies of Ce‐2‐MeIm/O_3_, Fe‐2‐MeIm/O_3_, and CeFe‐2‐MeIm/O_3_ were 79%, 91%, and 93%, respectively. The small difference between the Fe‐containing monometallic and bimetallic systems provides limited evidence of cooperative bimetallic synergy according to the validation framework presented in Section 3 and Figure 12. The decrease in COD removal from 93% to approximately 78% over four cycles further suggests limited stability. This may be related to the coordination mismatch between high‐valent Ce or Fe centers and imidazolate donors [64], particularly because Ce commonly adopts coordination numbers higher than those associated with tetrahedral ZIF‐like nodes [142, 274]. Replacing imidazolate with carboxylate linkers may improve the structural integrity and long‐term recyclability of the framework.

The catalytic degradation of levofloxacin (LEF) by Ce‐doped NH_2_‐MIL‐88B(Fe) (84.2%), exceeding that achieved by O_3_ (57.3%), Fe‐MOF/O_3_ (47.2%), and Ce‐MOF/O_3_ (14.7%), suggests cooperative rather than additive synergy [262]. Linker functionalization significantly modulated the framework's catalytic activity, with the unmodified, hydroxy‐, and amino‐functionalized materials achieving 34.3%, 66.6%, and 84.2%, respectively. This trend suggests that linker substituents regulate ozone adsorption, local electron density, and access to the metal centers. The system showed resilience across coexisting constituents (Cl^−^, HCO_3_^−^, HPO_4_^2−^, and HA) and in complex matrices (municipal tap water, Yellow River water, and artificial lake water), collectively supporting its real‐world applicability. The proposed mechanism invoked Ce‐mediated Fe(III)/Fe(II) cycling; however, evaluating the redox potential of the Ce(IV)/Ce(III) couple under the reaction conditions would help confirm the proposed redox synergy.

Ce incorporation into MIL‐53(Fe) introduced oxygen vacancies (OVs) and CUMSs [155]. DFT calculations indicated stronger O_3_ adsorption over OVs (−2.57 eV) than over the metal centers (−2.09 eV) in the BMOF, which is expected to facilitate subsequent ozone activation. Accordingly, atrazine (ATZ) removal reached 57.9%, 63.3%, and 91% for Fe‐MOF/O_3_, Ce‐MOF/O_3_, and CeFe‐BMOF/O_3_, respectively, within 5 min. However, comparison with a physical mixture of the two monometallic MOFs would be needed to distinguish cooperative effects from additive contributions. In situ Raman spectroscopy revealed the formation of surface‐adsorbed oxygen species (*O_2ads_ and *O_ads_) upon O_3_ exposure, both proposed to exhibit high oxidation potentials capable of oxidizing ATZ. The Ce 3d XPS spectrum was assigned almost entirely to Ce(III); however, peaks near ∼882 and ∼901 eV are often associated with Ce(IV) [275, 276, 277], leaving some ambiguity in the assigned oxidation state and the subsequent mechanistic interpretation. Despite these uncertainties, the BMOF retained substantial ATZ removal over four cycles, with undetectable iron leaching and ∼0.79 mg L^−1^ cerium leaching, indicating good structural stability and practical potential.

Although various metals, including Co, Ni, Fe, Mn, and Ce [278], have demonstrated promising catalytic activity toward ozonation, the application of BMOFs remains constrained by limited experimental verification of true bimetallic synergy, insufficient mechanistic understanding, ambiguous structural assignment in some systems, and concerns regarding hydrolytic stability. Future studies should combine rigorous control experiments, advanced structural characterization, and rational selection of complementary metal pairs and linker chemistries to establish reliable structure–activity relationships, broaden the range of effective BMOFs, and develop durable catalysts for practical water treatment. Table 3 summarizes the reported BMOFs for wastewater treatment via catalytic ozonation.

### 3.5 Peracetic Acid (PAA) Activation

Peracetic acid (CH_3_C(=O)OOH) is a powerful oxidant known for its superior disinfection capability [279], offering high efficiency while producing less toxic byproducts than chlorine gas [280]. The lower O─O bond energy of PAA (159 kJ mol^−1^), relative to H_2_O_2_ (213 kJ mol^−1^) and PMS (317 kJ mol^−1^) [225], together with its asymmetric structure, facilitates more facile homolytic cleavage and thus more efficient activation. PAA activation generates hydroxy radicals (^•^OH) and various carbon‐centered radicals (RO^•^), including acetoxyl (CH_3_C(═O)O^•^) and acetylperoxy (CH_3_C(═O)OO^•^) radicals [281, 282]. Various metal ions, such as Cu, Fe, Co, Mo, and Mn, as well as zerovalent Co and Cu, have demonstrated remarkable activity toward PAA activation [279].

PAA is prepared through the equilibrium reaction between acetic acid (AA) and H_2_O_2_ over approximately 24 h; therefore, their individual roles in the catalytic oxidation process should be evaluated independently [159]. Additionally, the molar ratio of AA to H_2_O_2_ should be carefully optimized, as residual H_2_O_2_ can scavenge reactive species and consequently inhibit the overall catalytic efficiency [145].

FeMn‐BTC metal–organic framework gel (MOG) was reported for PAA activation toward ofloxacin (OFX) degradation [159]. MOGs, colloidal networks of discrete crystalline nanoparticles interconnected through weak noncovalent interactions, exhibit structural characteristics of both MOFs and gels [42]. Increasing the Mn content progressively transformed the pore structure from predominantly microporous to mesoporous, which may facilitate the mass transfer of both pollutants and PAA to the active sites [242]. Consequently, the degradation efficiency increased from 57% for Fe‐BTC to 82% for FeMn‐BTC, with CH_3_C(═O)OO^•^ identified as the dominant reactive species. In addition to the individual activation of PAA by Fe(III) and Mn(II) (Figure 21c), synergistic interactions between the metal centers and residual H_2_O_2_ accelerated the redox cycles, thereby enhancing PAA activation.

Isomorphous substitution of Fe in MIL‐100(Fe) with Co significantly enhanced SMX removal from negligible levels to 86% within 30 min [263]. EPR measurements revealed a seven‐line DMPOX signal, suggesting a highly oxidative reaction environment [255]. However, the leached Co ions (2.8 µM, ∼0.16 mg L^−1^), which accounted for ∼55.6% of SMX degradation, suggest a dominant homogeneous contribution to the overall degradation process. These findings point to limited stability of the BMOF under the reaction conditions. More hydrolytically robust BMOFs, following the design principles outlined in Section 2.5, may help address this limitation in future studies.

A series of FeM‐BDC BMOFs (M = Co, Mn, Cu, and Ni) was evaluated for PAA‐mediated degradation of the pharmaceutical bezafibrate (BZF) [145]. Among the FeM‐BDC series, catalytic activity strongly depended on the secondary metal, with FeCo‐BDC showing the highest BZF removal (98.1%), clearly outperforming Co‐BDC (83.7%) and the remaining Fe‐based analogues (12.8%–30.1%). The enhanced performance of FeCo‐BDC was attributed to Fe–Co redox interactions (E°_Fe(III)/Fe(II)_ = +0.77 V and E°_Co(III)/Co(II)_ = +1.92 V vs. NHE) [242], supported by changes in the Fe and Co oxidation states after reaction. However, the coexistence of diffraction peaks and morphological features associated with both Fe‐BDC and Co‐BDC suggests that residual monometallic phases may be present. Nevertheless, FeCo‐BDC maintained >90% BZF removal over eight consecutive cycles and in the presence of various coexisting ions (SO_4_^2−^, NO_3_^−^, Ca(II), Mg(II), Cu(II), and Zn(II)), underscoring its structural integrity and practical potential.

A magnetic Fe_3_O_4_@Co/Zn‐2‐MeIM composite, in which Fe_3_O_4_ serves primarily to enable magnetic separation, was developed for PAA activation toward SMX degradation [149]. Zn‐2‐MeIM (ZIF‐8)/PAA showed limited activity, whereas Co‐2‐MeIM (ZIF‐67)/PAA and CoZn‐2‐MeIM (CoZn‐ZIF)/PAA achieved 96.7% and 98.8% removal within 30 min, respectively. These results identify Co as the main PAA activation center and indicate that Zn incorporation provides only a small increase in catalytic activity. Its more important contribution was the reduction of Co leaching from approximately 0.5 to 0.07 mg L^−1^, suggesting improved framework stability after Zn incorporation [283]. Because ZIF‐8 and ZIF‐67 share the same sodalite topology and closely related diffraction patterns (Figure 21d), PXRD alone cannot distinguish a phase‐pure CoZn‐BMOF from a physical mixture or spatially segregated structure, as discussed in Section 2.6. The uniform particle morphology and homogeneous Co/Zn distribution observed by EDX support mixed‐metal incorporation but do not conclusively establish the local arrangement of Co and Zn within the same framework. Local‐structure techniques such as EXAFS would provide stronger confirmation. As evidenced by XPS analysis, the Fe(II) centers from the Fe_3_O_4_ core facilitated the reduction of Co(III) to Co(II) through interfacial electron transfer. Future studies could examine whether controlled incorporation of Fe into the ZIF framework, rather than using an external Fe_3_O_4_ core, improves redox coupling while retaining low metal leaching and magnetic recoverability.

Although the application of BMOFs in PAA activation remains in its infancy, the reported systems demonstrate the potential of rational bimetallic design to enhance catalytic activity and stability. Future studies should prioritize rigorous verification of true BMOF formation, mechanistic elucidation of metal–metal redox synergy, and the development of more hydrolytically robust frameworks to suppress metal leaching and facilitate practical water treatment applications. Table 3 summarizes the reported BMOFs for wastewater treatment via peracetic acid activation.

### 3.6 Integrative Framework for BMOF‐AOPs

The BMOF‐AOP systems discussed in this review comprise photocatalysis together with five oxidant‐based systems (H_2_O_2_ activation in heterogeneous Fenton catalysis, PMS activation, PDS activation, catalytic ozonation, and PAA activation). Although these systems involve different oxidants and reaction pathways, their catalytic behavior can be described through a common sequence: metal‐pair chemistry regulates the electronic structure and local coordination environment; these properties determine the activation pathway; the activation pathway controls reactive‐species generation; and the resulting species govern catalytic activity, selectivity, and stability (Figure 22).

**FIGURE 22:**
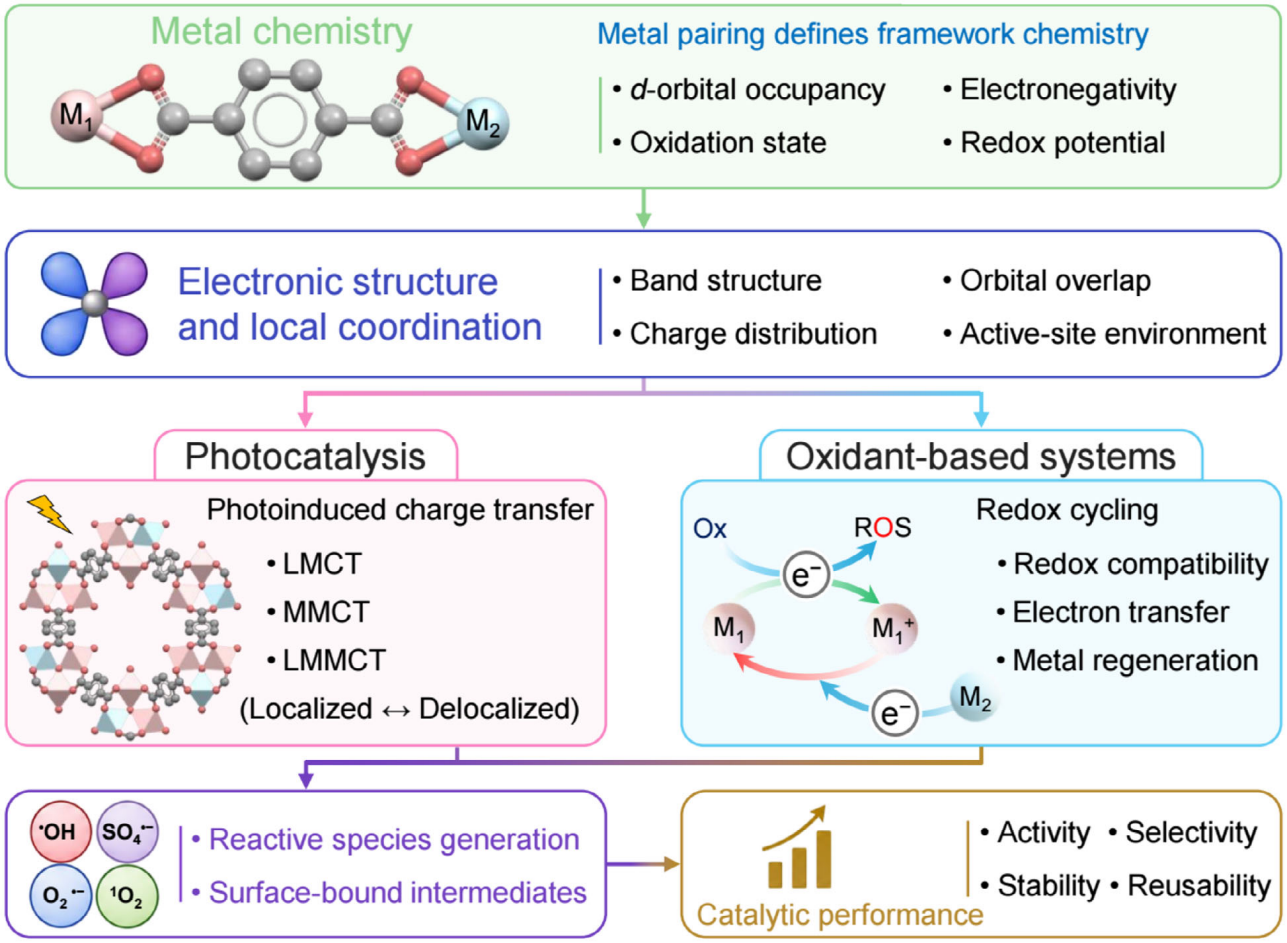
Integrative framework linking metal chemistry, electronic structure, activation pathways, reactive‐species generation, and catalytic performance in BMOF‐AOPs.

#### 3.6.1 Metal Chemistry and Electronic Structure

Metal pairing modulates the framework's electronic structure through differences in oxidation state, electronegativity, and d‐orbital occupancy. In photocatalytic BMOFs, this modulation is represented explicitly through the LMCT, MMCT, and combined LMMCT pathways (Section 3.1 and Figure 13), where the extent of orbital overlap and electronic localization between the two metal centers determines whether charge separation proceeds via a discrete donor‐acceptor mechanism or a delocalized excited state (Figure 14). In oxidant‐based systems, the electron‐transfer processes underlying redox cycling are generally facilitated by redox‐potential compatibility between the two metal species. For instance, owing to the lower reduction potential of Cu (*E*°_Cu(II)/Cu(I)_ = +0.16 V vs. NHE) relative to Fe (*E*°_Fe(III)/Fe(II)_ = +0.77 V vs. NHE) and Co (*E*°_Co(III)/Co(II)_ = +1.92 V vs. NHE), Cu(I) accelerates Fe(III)/Fe(II) and Co(III)/Co(II) cycling in heterogeneous Fenton catalysis and sulfate radical‐based systems, respectively [5, 205, 208, 234]. Similarly, the compatible Fe(III)/Fe(II) and Co(III)/Co(II) redox potentials in PAA activation were proposed to explain the markedly stronger performance of FeCo‐BDC relative to other FeM‐BDC pairs [145]. Exploring additional metals with redox potentials compatible with the primary metal activator may result in more effective couples for oxidant‐based systems.

#### 3.6.2 Selective Oxidant Activation

The electronic and coordination properties of each metal influence its role in oxidant adsorption, electron transfer, and bond cleavage. In many BMOFs, one metal acts mainly as the direct oxidant‐activation site, while the second promotes regeneration of the active oxidation state, modifies the electron density, or changes oxidant adsorption. Co‐containing frameworks are frequently effective for PMS and PAA activation, whereas Fe‐based centers are commonly used for H_2_O_2_ and PDS activation (Section 3.2 and Figure 17). Ce(III)/Ce(IV), through its accessible redox couple and role in generating oxygen vacancies and Lewis‐acid sites, accelerates O_3_ activation through the formation of structural and surface intermediates [260]. These trends are not universal. The same metal may act as a direct activation center in one framework but mainly as an electronic regulator in another because linker chemistry, coordination geometry, metal distribution, and pore accessibility alter its catalytic role. For example, Cu can directly participate in PMS activation in some BMOFs but contribute mainly to redox regeneration in others [236]. The functional role of each metal should therefore be established using appropriate monometallic controls, physical‐mixture comparisons, oxidation‐state measurements, and electronic‐structure calculations (Figure 20).

#### 3.6.3 Reactive‐Species Generation

The activation pathway determines the types and relative contributions of the reactive species produced. In photocatalytic systems, excited electrons may reduce dissolved O_2_ to O_2_^•−^, while photogenerated holes can directly oxidize pollutants or contribute to ^•^OH formation. In heterogeneous Fenton systems, Fe‐mediated H_2_O_2_ activation is commonly associated with ^•^OH generation. PMS‐ and PDS‐based systems may produce SO_4_^•−^, ^•^OH, ^1^O_2_, or surface‐bound reactive complexes, depending on the metal pair and reaction conditions. Catalytic ozonation can involve •OH, surface oxygen intermediates, or direct ozone‐mediated oxidation, whereas PAA activation may generate organic radicals, •OH, ^1^O_2_, or high‐valent metal species

The data summarized in Tables 1, 2, 3 indicate broad relationships between metal composition and reactive‐species generation, but these should not be interpreted as fixed metal‐to‐species rules. The dominant pathway can change with pH, oxidant dosage, linker functionalization, defect density, and water‐matrix composition. Moreover, scavenger tests and individual EPR signals are rarely sufficient to establish a complete mechanism. For example, DMPOX indicates a strongly oxidative environment but is not specific to a single reactive species. Reliable assignments therefore require complementary evidence from selective probes, isotope experiments, kinetic analysis, in situ spectroscopy, and computational studies.

Overall, the sequence of metal chemistry → electronic structure → activation pathway → reactive species → catalytic performance provides a unified basis for comparing photocatalytic and oxidant‐based BMOF systems. This framework also shows that bimetallic cooperation cannot be predicted from metal identity alone. The local coordination environment, framework structure, metal distribution, and reaction conditions must be considered together when designing BMOFs for selective and stable wastewater treatment.

## 4 Conclusions and Outlook

BMOFs have emerged as a promising platform for AOPs by combining the intrinsic structural advantages of MOFs, including high surface area, permanent porosity, and tunable pore geometry, with the synergistic effects of multiple metal centers. The introduction of a second metal can generate complementary redox, electronic, structural, and adsorption‐catalytic synergies. Consequently, heterometallic pairs can deliver performance that frequently exceeds their monometallic counterparts. This review decodes the synergistic pathways governing BMOF performance across five AOP systems and establishes the relationships among metal chemistry, linker coordination, framework structure, and catalytic activity that underpin rational BMOF design.

The successful synthesis of BMOFs requires careful balancing of metal competition, HSAB compatibility, and hydrolytic stability. Although PSME and CSBU approaches offer more precise metal incorporation, OPR remains predominant and frequently yields mixed‐phase products, reinforcing the need for rigorous multi‐technique phase validation. Progress toward sustainable and scalable synthesis—through linker salts, metal waste streams, and plastic upcycling—has enabled solvent‐free, gram‐scale production; however, large‐scale products often exhibit reduced crystallinity, and plastic upcycling routes still rely on harsh conditions. Integrating ambient depolymerization strategies into BMOF formation represents a promising path toward genuinely green synthesis. A unified design framework and a systematic characterization approach for unambiguous phase validation were proposed to guide future BMOF development.

Across all AOP systems, metal doping enhances catalytic performance through several recurring mechanisms, including charge separation, electron‐density modulation, bandgap narrowing, redox‐cycle promotion, simultaneous oxidant activation, and Lewis acidity engineering. The proposed oxidant‐activation pathways provide an initial framework for relating the roles of the two metal centers to catalyst design. However, a general electronic‐structure model for BMOFs has not yet been established, and the extent to which the metal‐derived states remain localized, become electronically coupled, or form delocalized orbitals remains poorly understood. Future studies should combine orbital‐resolved calculations with time‐resolved and operando spectroscopy to determine how metal identity, local coordination, oxidation state, and metal–ligand coupling regulate charge transfer and oxidant activation at the atomic level. BMOF research in SR‐AOPs remains heavily focused on Co‐based systems. Expanding toward lower‐toxicity metal pairs and integrating alternative energy sources beyond visible light represent promising directions for future research.

Mechanistic assignments in BMOF‐AOP systems still rely heavily on ex situ characterization and indirect scavenging experiments. Future studies should combine time‐resolved and operando methods with simultaneous monitoring of catalytic performance. Operando XAS, Raman, infrared spectroscopy, and EPR can track changes in metal oxidation state, coordination environment, oxidant adsorption, and reactive intermediates, while transient absorption spectroscopy can resolve short‐lived charge‐transfer processes in photocatalytic systems. Light‐induced or ambient‐pressure XPS may provide complementary surface information where experimentally feasible. Correlating these measurements with online analysis of oxidant consumption and pollutant transformation will help identify the active catalytic state and establish more reliable structure–activity relationships.

A recurring limitation of BMOFs in AOPs is their structural instability under aqueous and oxidative conditions. Hydrolysis of metal–linker bonds is often the main cause, particularly in frameworks containing weakly coordinated divalent metals such as Co(II), Fe(II), and Cu(II), although ligand protonation, oxidative linker degradation, defect accumulation, and redox‐induced restructuring may also contribute. These processes can cause metal leaching, loss of crystallinity, pore collapse, and homogeneous catalytic pathways that complicate the interpretation of bimetallic synergy. Addressing this challenge requires the hydrolytic‐stability design principles outlined in Section 2.5 (Figure 10), including HSAB‐guided metal–linker selection, bifunctional carboxylate/N‐donor linkers capable of simultaneously stabilizing high‐ and low‐valent metal centers, and the use of intrinsically robust frameworks such as MILs and ZIFs with their compatible metals.

Although BMOFs have shown promising activity in real water matrices and in the presence of coexisting species, most studies remain limited to short batch tests. Future work should focus on genuinely contaminated effluents, scalable catalyst recovery, metal leaching, regeneration, and extended continuous‐flow operation to establish long‐term stability and practical reliability.

Overall, BMOFs provide a versatile platform for next‐generation AOPs by integrating structural tunability with cooperative catalytic mechanisms. Continued progress will depend on combining rational framework design, rigorous mechanistic validation, hydrolytically robust architectures, and scalable synthesis to translate laboratory discoveries into practical wastewater treatment technologies.

## Funding

K. El‐Naggar is funded by a full Ph.D. scholarship from the Ministry of Higher Education and Scientific Research of the Arab Republic of Egypt. This work was funded by the Australian Laureate Fellowships (FL230100178) and Discovery Early Career Researcher Award (DE240100660) from the Australian Research Council.

## Conflicts of Interest

The authors declare no conflicts of interest.

## Data Availability

The data that support the findings of this study are available from the corresponding author upon reasonable request.
